# Metal-Free, PPA-Mediated Fisher Indole Synthesis via Tandem Hydroamination–Cyclization Reaction between Simple Alkynes and Arylhydrazines

**DOI:** 10.3390/ijms25168750

**Published:** 2024-08-11

**Authors:** Alexander V. Aksenov, Dinara C. Makieva, Rodion A. Arestov, Nikolai A. Arutiunov, Dmitrii A. Aksenov, Nicolai A. Aksenov, Alexander V. Leontiev, Inna V. Aksenova

**Affiliations:** Department of Chemistry, North Caucasus Federal University, 1a Pushkin St., 355017 Stavropol, Russianaarutiunov@ncfu.ru (N.A.A.); daksenov@ncfu.ru (D.A.A.);

**Keywords:** fisher indoles, cascade transformations, alkynes hydroamination, cyclization, metal-free, alkyne hydrolysis, arylhydrazines

## Abstract

A new variant of Fisher indole synthesis involving Bronsted acid-catalyzed hydrohydrazination of unactivated terminal and internal acetylenes with arylhydrazines is reported. The use of polyphosphoric acid alone either as the reaction medium or in the presence of a co-solvent appears to provide the required balance for activating the C–C triple bond towards the nucleophilic attack of the hydrazine moiety without unrepairable reactivity loss of the latter due to competing amino group protonation. Additionally, the formal hydration of acetylenes to the corresponding ketones occurs under the same conditions, making it an alternative approach for generating carbonyl groups from alkynes.

## 1. Introduction

**Scheme 1:**
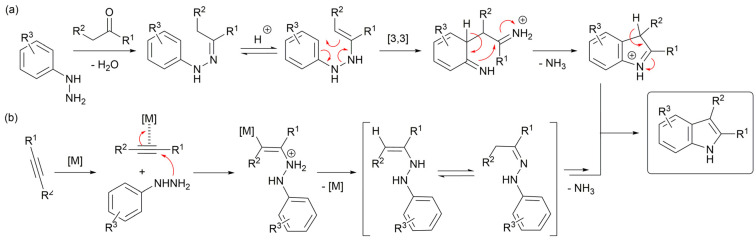
Classical Fischer indole synthesis (**a**) vs. mechanistically related metal-catalyzed alkyne hydroamination-based (**b**) indole synthesis.

**Scheme 2:**
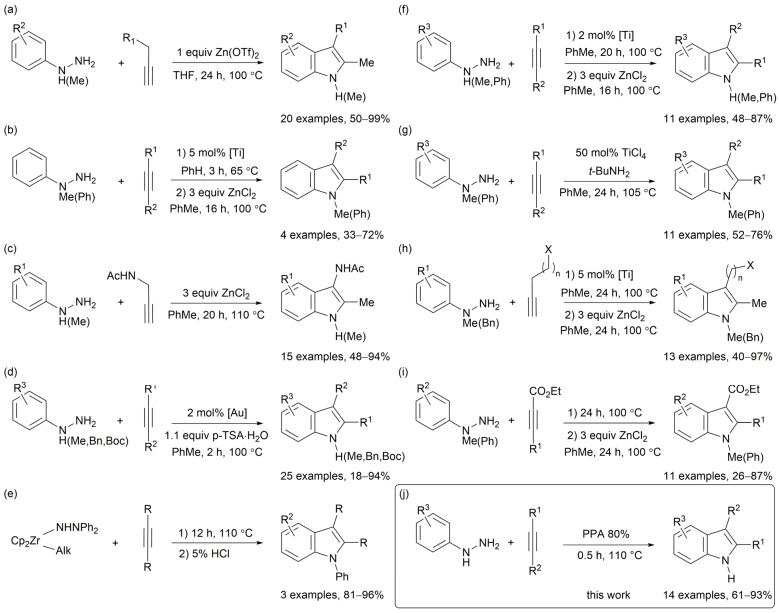
Previous ((**a**) [20], (**b**) [21], (**c**) [22,23,24], (**d**) [25], (**e**) [26], (**f**) [27,28], (**g**) [29], (**h**) [30,31,32], (**i**) [33]) and current (**j**) works on synthesizing Fisher-type indoles via domino, hydrohydrazination-cyclization reactions starting from alkynes and arylhydrazines.

The vast number of interesting biologically active indole-containing systems in both natural products and pharmaceutically relevant compounds [1,2,3] is the main reason for the ongoing attention to developing de novo synthesis of these molecules [4]. Among the constellation of methodologies known today [5,6,7,8,9], the classic Fischer indole synthesis (Scheme 1a), as well as its modern variants [4,10,11,12], continue to represent valuable and effective approaches for accessing a wide range of these heterocyclic scaffolds [13,14]. One subclass among this type of reaction is based on the transition metal-catalyzed hydroamination [15,16] of alkynes with various nitrogen sources [17,18,19], including arylhydrazines. Although in the latter case (Scheme 1b), it does not eliminate the primary difficulty associated with the Fischer indole synthesis such as the limited commercial availability of the hydrazine substrates, alkynes themselves are generally readily accessible and inexpensive starting materials. Thereby, their use imposes an additional level of synthetic practicality to those Fisher-type reactions and constitutes a valuable alternative route to the indole scaffold. In turn, we would like to report here the first, to our knowledge, example of a metal-free, Bronsted acid-mediated hydroamination of alkynes with arylhydrazines, which, when followed by a one-pot Fisher-type cyclization, results in a variety of indoles in good to excellent yields (Scheme 2j).

## 2. Results and Discussion

Above is a summary of known synthetic approaches toward indoles via the metal-mediated domino hydrohydrazination–Fischer indolization reaction between arylhydrazines and alkynes (Scheme 2a–i) [20,21,22,23,24,25,26,27,28,29,30,31,32,33]. Beginning more than 30 years ago as a mere organometallic curiosity [26]—the hydrolytic decomposition of a product of an alkyne capture by a hydrazidozirconocene complex (Scheme 2e)—the underlying methodology has evolved significantly in recent years, overgrowing many of its initial drawbacks. Thus, at first, these were step-wise, not very practical protocols [21,29,30,31,32], using titanium chelates, which were generally moisture-sensitive, compatible only with protected arylhydrazine, and exhibited low functional groups tolerance (Scheme 2b,g,h). Further developments in this area have led to a new generation of Ti catalysts that work well with both alkyl- and aryl- monosubstituted hydrazines, thereby allowing the preparation of NH–indoles as well as some other nitrogen heterocycles (Scheme 2f). Also, it was shown that the one-pot synthesis of substituted indoles does not require titanium catalysts at all. Apparently, Zn salts alone are excellent at promoting both the hydrohydrazination of alkynes and the following Fischer indole cyclization (Scheme 2a) [20]. In turn, the binary system of Ph_3_PAuNTf_2_ and *p*-toluenesulphonic acid monohydrate (*p*-TSA·H_2_O) proved to be very effective in a single-stage synthesis of 2,3-disubstituted indoles from alkynes and arylhydrazines (Scheme 2d) [25]. Meanwhile, it should be noted that the latter can also react via non-Fisher-type pathways [34,35,36,37] (i.e., without the formation of key enehydrazine intermediates) which may provide access to a variety of substituted indoles with a regioselectivity different from that of the Fischer synthesis.

While any of the above methods are quite functional, almost all of them require up to 3 equiv of zinc halides to furnish the second stage—Fisher indolization. Although Zn salts are generally cheap and readily available, this excessive stoichiometry largely negates the benefits of the catalytic nature of the first step—alkyne hydrohydrazination—which occurs in the presence of only 2–5 mol% of titanium complexes.

Given our extensive experience in the chemistry of heterocycles, including indoles one [38,39], we became interested in the possibility of carrying out this kind of conversion (alkyne hydrohydrazination–Fisher cyclization) metal-free, by using Bronsted acid alone, preferably in an eco-friendly manner [40]. To do this, obviously, a delicate balance, mainly by choosing an acid of certain strength, must be achieved to, from the one side, ensure the activation of the C–C triple bond and, on the other, maintain the reactivity of the amino group. The literature review revealed that there are many examples of transition metal-catalyzed [15,16,41,42,43,44,45], base-assisted [46], and organohalide-activated [47] hydroamination of alkynes, while the Bronsted acid-promoted addition of amines to non-activated alkynes, unlike olefines [48,49,50,51], are practically unknown [52]. We speculated that polyphosphoric acid (PPA) might be a suitable candidate for this role, since in a number of our previous works, it has been found to be quite compatible with a wide range of *O*- and *N*-nucleophiles, such as anilines [53], phenols [54], aliphatic amines [55], or acylhydrazides [56]. In addition, PPA being a moderately strong, non-oxidizing acid, capable of dissolving organic compounds and having powerful dehydrating properties, is well known as an effective reagent, catalyst, or reaction medium for numerous synthetic applications [57,58]. Being both commercially available and readily prepared [58,59] *in-house* (by dissolving calculated amounts of P_2_O_5_ in 85% orthophosphoric acid), PPA is generally used either as an emulsion in inert high-boiling solvents (xylenes, toluene, etc.) [40,60], silica gel-supported [61] reagent, or as a reaction medium [58], typically in a 10–50-fold excess (by weight).

Therefore, we decided to use the reaction of phenylhydrazine **1a** with phenylacetylene **2a** in the presence of PPA as a model one. The results of these screening experiments are given in Table 1.

As can be seen from Table 1, as expected, the use of a 10-fold excess of 80% PPA (as both catalyst and solvent), allowed the reaction to be completed within 30 min, with almost quantitative yields (entries 1–3). At the same time, a decrease below this threshold slightly reduces the latter (entry 4). It should be noted that although the water balance does not change during the process, as in the case of the classical Fisher reaction (Scheme 1a), ammonia as a by-product is still released, deactivating the acid and, thereby, preventing its use in catalytic quantities. From this point of view, the conditions found in entry 3 appear to be optimal, since doubling the amount of 80% PPA (entry 5) or utilizing 100% H_3_PO_4_ or 87% PPA instead (entries 6 and 7, respectively) either did not lead to an overall improvement (entry 5) or gave disappointing results (entry 6,7). In addition, the use of 100% phosphoric (entry 8) or 80% polyphosphoric (entry 9) acids in the presence of co-solvents (ethanol and toluene, respectively) is also possible; although, if in the case of the former (entry 8), a noticeable deterioration of the reaction mixture and significant drop in yield (33%) were observed.

It should be noted that the main difference between 80 and 87% PPA is its composition [57]. The former consists mainly of ortho-, pyro-, tri-, and tetraphosphoric acids (*n* = 1–4), while “strong” PPA (above 86 w.t. % of P_2_O_5_) is composed largely of high molecular weight, linear as well as cyclic, polymeric (n > 7) species. In our experience, it is hard to say at the outset which type of PPA will perform better in a particular reaction, so as a rule, it is necessary to test both options. And since the precise composition of polyphosphoric acid and the detailed mechanism of transformation catalyzed by PPA are usually unknown, only speculative assumptions can be made about the role of PPA concentration in any given reaction.

However, an intriguing aspect was the possibility of running this reaction as an emulsion in toluene (entry 9). The latter means that the indole synthesis described herein can be conducted without the need for aqueous post-treatment and be easily scaled up. Thus, according to the leading reported work [40], using toluene as a cosolvent and only a 3-fold excess (not 10!) of PPA (*w*/*w*), up to 3 kg of indole derivatives were prepared in one batch via the classical Fisher reaction between ketones and arylhydrazines. The key factor here is the precipitation of PPA at the bottom of the reactor after the reaction is complete. The authors then simply separate the upper toluene layer and remove solvent under reduced pressure, obtaining the target products in high yields (up to 99%) and purity (>95%). We believe that the same methodology can be applied to our conceptually very similar PPA-assisted reaction between alkynes and arylhydrazines.

**Scheme 3:**
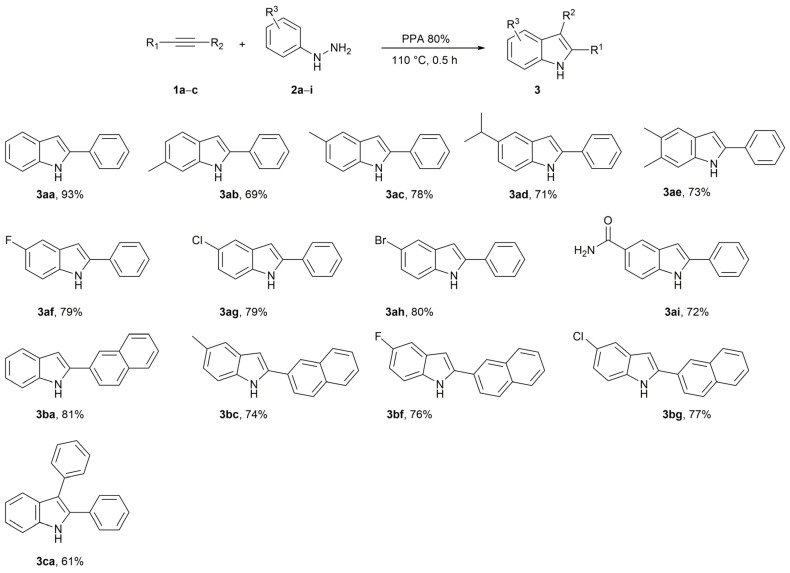
A set of indoles **3** prepared by the reaction between acetylenes **1** and arylhydrazines **2**.

At this point, with the working conditions in hand, we were ready to assemble a small library of indoles **3** (Scheme 3). In terms of scope and general applicability, the results obtained are rather similar to those we observed previously in the PPA-assisted Fisher indole synthesis by the reaction of arylhydrazines with acetophenones [62]. The benchmark, unsubstituted phenylhydrazine **2a**, gives the target indole **3aa** in an excellent yield of 93%. In turn, the introduction of donor alkyl substituents as in **2b**–**e** or the use of arylhydrazines **2f**–**i** bearing the electron-withdrawing groups did not have a significant effect on the overall outcome of the process, providing yields of 69–81%. Regarding acetylenes, the terminal (2-naphthyl)acetylene **1b** gave the corresponding indole **3ba** in a respectable 81% yield, while the internal alkyne **1c** performed a bit worse (61% of **3ca**). However, such inner alkynes are much more accessible [63] than the corresponding arylbenzyl ketones, which is a significant advantage.

Also, in our experience the substrate/PPA ratio of 1 to 10 by weight is not something immutable, and sometimes the amount of polyphosphoric acid could be significantly reduced (up to 3 times, for example, as in the case of 2-naphthylindole **3ba**), as long as the viscosity of the reaction mixture both at the beginning and at the end of the reaction, when ammonium phosphates accumulate, allows for proper stirring.

**Scheme 4:**
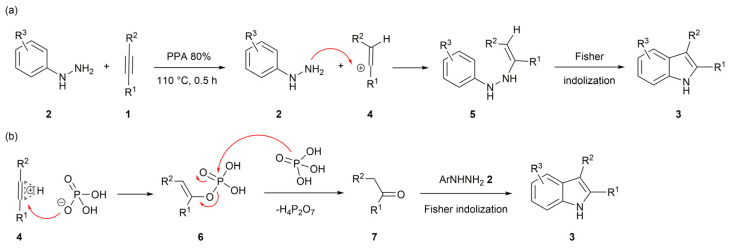
Plausible mechanisms for the formation of indoles **3**.

Plausible mechanisms for this cascade transformation are presented in Scheme 4. The first possible pathway is associated with the direct nucleophilic attack of hydrazine **2** on vinyl cation **4** generated from acetylenes **1** in PPA (Scheme 4a). The resulting enhydrazine **5** obviously undergoes the classical Fischer reaction under the reaction conditions.

Another feasible route (Scheme 4b) suggests that ketone **7** could be formed directly under anhydrous conditions by acidolysis of vinyl phosphate intermediate **6** in a manner similar to how carboxylic acids and vinyl acetates react in the presence of an acid catalyst to form mixed anhydrides and ketones [64,65]. However, no special attempts have been made to distinguish between these two possible routes or to isolate or observe the intermediate vinyl phosphates.

To further evaluate the possible mechanism of the discussed PPA-assisted tandem synthesis of indoles, blank experiments were carried out with phenylacetylene **1a** alone as a model substrate under the same conditions (Table 2).

The obtained results correlate well with those of the indole synthesis (Table 1). In each case, after aqueous workup, the corresponding acetophenone **7a** was usually formed in good to excellent yields. Although polyphosphoric acid (PPA 80%) was still found to be the most suitable in terms of yields (entries 1–5), this time we did not observe any changes at different acid-to-substrate ratios. This is most likely due to the absence of ammonia, which is released during the Fisher synthesis and acts as an acid trap. Finally, the rate of hydrolysis we observed was significantly lower than the rate of the tandem hydroamination-indolization reaction (1.5 vs. 0.5 h) discussed above, suggesting that the latter occurs presumably through the direct attack of hydrazine on the acetylene moiety (Scheme 4a) rather than the formation of ketone product **7** first (Scheme 4b).

**Scheme 5:**
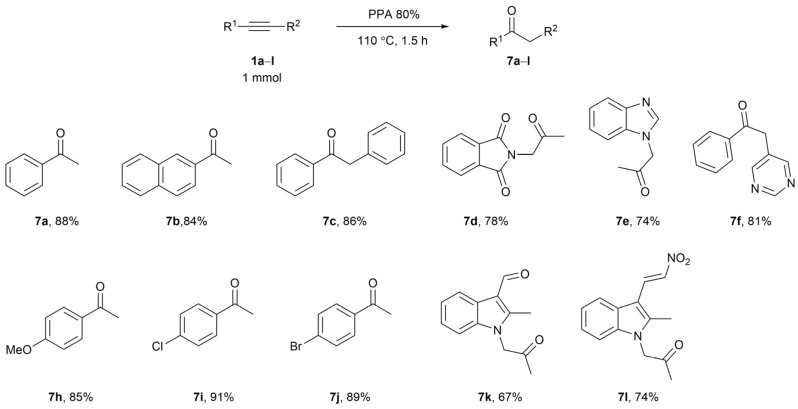
Acetophenones **7** prepared by hydrolysis of acetylenes **1** in PPA medium.

To demonstrate the general applicability of this protocol, a set of 11 acetophenones **7**, including 3 new ones (**7f**,**k**,**l**), were prepared (Scheme 5). Overall, the reaction proceeded smoothly with slight differences in yields depending on the structure of the starting acetylenes. Thus, both simple donor- and acceptor-substituted phenylacetylenes **1a**,**h**–**j** provide equally high yields of the corresponding acetophenones **7a**,**h**–**j**. The synthesis of ketones like **7d** and **7e** may be particularly useful here, since the standard approach to such compounds involves the alkylation of the benzimidazole or phthalimide derivatives with the corresponding, usually highly lacrimal α-haloketones. Another example of a synthetically valuable approach is the PPA-assisted hydrolysis of the Sonogashira adduct **1f**, which results in the corresponding arylheteroaryl ketone **7f**.

Overall, we think that although the synthesis of simple ketones is generally not a particularly challenging task, and many excellent methods exist to accomplish it, including the classical Kucherov reaction [66,67], the given procedure will be a valuable addition to the current arsenal of proven synthetic protocols.

**Scheme 6:**
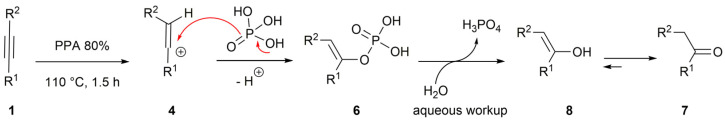
Probable mechanism of PPA-assisted conversion of alkynes **1** into ketones **7**.

The plausible reaction mechanism is shown in Scheme 6. As PPA is known for its strong dehydrating properties, it seems unlikely that this reaction proceeds through the classical Lewis/Bronsted acid-catalyzed alkyne hydration pathway also referred to as the Kucherov reaction [66,67]. Arguably, it involves the protonation of acetylene **1,** followed by the attack of phosphoric acid on the vinyl cation **4**. After treatment with water, the vinyl phosphate **6** is released in the form of the corresponding enol **8**, which then quickly tautomerizes to ketone **7**.

## 3. Materials and Methods

### 3.1. General Information

NMR spectra, ^1^H, and ^13^C were measured in solutions of CDCl_3_ or DMSO-*d*_6_ on a Bruker AVANCE-III HD instrument (at 400 and 101 MHz, respectively). Residual solvent signals were used as internal standards, in DMSO-*d*_6_ (2.50 ppm for ^1^H, and 40.45 ppm for ^13^C nuclei) or CDCl_3_ (7.26 ppm for ^1^H, and 77.16 ppm for ^13^C nuclei). HRMS spectra were measured on a Bruker maXis impact (electrospray ionization, in MeCN solutions, employing HCO_2_Na–HCO_2_H for calibration). IR spectra were measured on a FT-IR spectrometer Shimadzu IRAffinity-1S equipped with an ATR sampling module. See Supplementary Materials for the NMR (Figures S1–S46) and HRMS (Figures S47–S53) spectral charts. Reaction progress, purity of isolated compounds, and R*_f_* values were monitored with TLC on Silufol UV-254 plates. Column chromatography was performed on silica gel (32–63 μm, 60 Å pore size). Melting points were measured with the Stuart SMP30 apparatus. Acetylenes **1d** [68], **1e** [69], and **1f** [70] were synthesized according to the previously reported procedures and were identical to those described. All other reagents and solvents were purchased from commercial vendors and used as received.

### 3.2. Preparation of Indoles ***3*** (General Procedure)

A 5 mL round-bottom flask equipped with a magnetic stir bar was charged with acetylene **1** (1.00 mmol), arylhydrazine **2** (1.00 mmol) (or its hydrochloride), and polyphosphoric acid (2 g, P_2_O_5_ 80 w.t. %) and stirred upon heating at 100 °C for 30 min (TLC control). After the reaction was complete, the mixture was poured into 80 mL of cold water and basified with 20% ammonia solution. After extraction with EtOAc (4 × 20 mL), the organic fraction was concentrated in vacuo, and the crude material was purified by column chromatography (EtOAc/Hexane).

2-Phenyl-1*H*-indole (**3aa**): this compound was prepared employing phenylacetylene **1a** (109 µL, 102 mg, 1.00 mmol) and phenylhydrazine **2a** (98 µL, 108 mg, 1.00 mmol) in a yield of 179 mg (0.93 mmol, 93%). Purification was performed by column chromatography (EtOAc/Hexane = 1:4). ^1^H NMR (400 MHz, DMSO-*d*_6_) δ 11.55 (s, 1H), 7.54 (d, *J* = 7.8 Hz, 1H), 7.49–7.40 (m, 3H), 7.32 (t, *J* = 7.4 Hz, 1H), 7.14–7.07 (m, 1H), 7.01 (t, *J* = 7.4 Hz, 1H), 6.90 (d, *J* = 2.2 Hz, 1H); ^13^C{^1^H} NMR (101 MHz, DMSO-*d*_6_) δ 137.7, 137.2, 132.3, 129.0 (2C), 128.7, 127.5, 125.0 (2C), 121.6, 120.1, 119.4, 111.4, 98.7. All of the spectral characteristics were identical to those described previously [71].

6-Methyl-2-phenyl-1*H*-indole (**3ab**): this compound was prepared employing phenylacetylene **1a** (109 µL, 102 mg, 1.00 mmol) and *m*-tolylhydrazine hydrochloride **2b** (158.5 mg, 1.00 mmol) in a yield of 143 mg (0.69 mmol, 69%). Purification was performed by column chromatography (EtOAc/Hexane = 1:4). ^1^H NMR (400 MHz, DMSO-*d*_6_) δ 11.38 (s, 1H), 7.92–7.72 (m, 2H), 7.44 (t, *J* = 7.6 Hz, 2H), 7.34–7.22 (m, 3H), 6.92 (dd, *J* = 8.2, 1.7 Hz, 1H), 6.80 (d, *J* = 2.2 Hz, 1H), 2.36 (s, 3H); ^13^C{^1^H} NMR (101 MHz, DMSO-*d*_6_) δ 138.0, 136.0, 132.8, 129.3 (2C), 128.3, 127.7, 125.3 (2C), 123.7, 120.1, 111.5, 98.7, 21.6. All of the spectral characteristics were identical to those described previously [72].

5-Methyl-2-phenyl-1*H*-indole (**3ac**): this compound was prepared employing phenylacetylene **1a** (109 µL, 102 mg, 1.00 mmol) and *p*-tolylhydrazine hydrochloride **2c** (158.5 mg, 1.00 mmol) in a yield of 161 mg (0.78 mmol, 78%). Purification was performed by column chromatography (EtOAc/Hexane = 1:4). ^1^H NMR (400 MHz, DMSO-*d*_6_) δ 11.07 (s, 1H), 7.94 (d, *J* = 7.8 Hz, 2H), 7.46 (t, *J* = 7.6 Hz, 2H), 7.35 (dd, *J* = 12.9, 7.3 Hz, 2H), 6.90 (t, *J* = 6.7 Hz, 3H), 2.55 (s, 3H); ^13^C{^1^H} NMR (101 MHz, DMSO-*d*_6_) δ 138.2, 137.1, 132.8, 129.2 (2C), 128.8, 127.8, 125.9 (2C), 122.7, 121.2, 120.1, 118.1, 100.0, 17.7. All of the spectral characteristics were identical to those described previously [73].

5-Isopropyl-2-phenyl-1*H*-indole (**3ad**): this compound was prepared employing phenylacetylene **1a** (109 µL, 102 mg, 1.00 mmol) and (4-isopropylphenyl)hydrazine hydrochloride **2d** (186.5 mg, 1.00 mmol) in a yield of 166 mg (0.71 mmol, 71%). Purification was performed by column chromatography (EtOAc/Hexane = 1:4). ^1^H NMR (400 MHz, DMSO-*d*_6_) δ 11.38 (s, 1H), 8.03–7.67 (m, 2H), 7.44 (t, *J* = 7.6 Hz, 2H), 7.35 (s, 1H), 7.33–7.27 (m, 2H), 7.07–6.93 (m, 1H), 6.82 (s, 1H), 3.01–2.85 (m, 1H), 1.25 (s, 3H), 1.23 (s, 3H); ^13^C{^1^H} NMR (101 MHz, DMSO-*d*_6_) δ 139.9, 138.1, 136.2, 132.8, 129.4 (2C), 129.2, 127.7, 125.3 (2C), 121.3, 117.2, 111.5, 99.0, 34.0, 25.1 (2C). All of the spectral characteristics were identical to those described previously [74].

5,6-Dimethyl-2-phenyl-1*H*-indole (**3ae**): this compound was prepared employing phenylacetylene **1a** (109 µL, 102 mg, 1.00 mmol) and (3,4-dimethylphenyl)hydrazine hydrochloride **2e** (172.5 mg, 1.00 mmol) in a yield of 161 mg (0.73 mmol, 73%). Purification was performed by column chromatography (EtOAc/Hexane = 1:4). ^1^H NMR (400 MHz, CDCl_3_) δ 8.19 (s, 1H), 7.65 (d, *J* = 6.1 Hz, 3H), 7.48–7.37 (m, 4H), 7.31 (t, *J* = 7.3 Hz, 1H), 7.18 (s, 1H), 6.73 (s, 1H); ^13^C{^1^H} NMR (101 MHz, CDCl_3_) δ 136.8, 135.7, 132.4, 131.3, 128.8 (2C), 127.4, 127.1, 124.7 (2C), 120.5, 111.1, 99.2, 20.4, 19.9. All of the spectral characteristics were identical to those described previously [75].

5-Fluoro-2-phenyl-1*H*-indole (**3af**): this compound was prepared employing phenylacetylene **1a** (109 µL, 102 mg, 1.00 mmol) and (4-fluorophenyl)hydrazine hydrochloride **2f** (162.5 mg, 1.00 mmol) in a yield of 167 mg (0.79 mmol, 79%). Purification was performed by column chromatography (EtOAc/Hexane = 1:4). ^1^H NMR (400 MHz, DMSO-*d*_6_) δ 11.64 (s, 1H), 7.85 (d, *J* = 7.7 Hz, 2H), 7.47 (t, *J* = 7.7 Hz, 2H), 7.42–7.23 (m, 3H), 6.93 (td, *J* = 9.2, 2.6 Hz, 1H), 6.89 (d, *J* = 2.2 Hz, 1H); ^13^C{^1^H} NMR (101 MHz, DMSO-*d*_6_) δ 157.6 (d, *J* = 231.2 Hz), 140.0, 134.3, 132.3, 129.4 (2C), 129.3 (d, *J* = 10.5 Hz), 128.2, 125.6 (2C), 112.7 (d, *J* = 9.9 Hz), 110.1 (d, *J* = 26.1 Hz), 105.0 (d, *J* = 23.2 Hz), 99.3 (d, *J* = 4.8 Hz). All of the spectral characteristics were identical to those described previously [76].

5-Chloro-2-phenyl-1*H*-indole (**3ag**): this compound was prepared employing phenylacetylene **1a** (109 µL, 102 mg, 1.00 mmol) and (4-chlorophenyl)hydrazine hydrochloride **2g** (179.0 mg, 1.00 mmol) in a yield of 179 mg (0.79 mmol, 79%). Purification was performed by column chromatography (EtOAc/Hexane = 1:4). ^1^H NMR (400 MHz, DMSO-*d*_6_) δ 11.75 (s, 1H), 7.85 (d, *J* = 7.7 Hz, 2H), 7.57 (d, *J* = 2.0 Hz, 1H), 7.47 (t, *J* = 7.6 Hz, 2H), 7.40 (d, *J* = 8.6 Hz, 1H), 7.35 (d, *J* = 7.3 Hz, 1H), 7.09 (dd, *J* = 8.6, 2.1 Hz, 1H), 6.89 (d, *J* = 2.1 Hz, 1H); ^13^C{^1^H} NMR (101 MHz, DMSO-*d*_6_) δ 139.8, 136.0, 132.1 (2C), 130.2, 129.5, 128.3, 125.6 (2C), 124.4, 121.9, 119.5, 113.2, 98.8. All of the spectral characteristics were identical to those described previously [76].

5-Bromo-2-phenyl-1*H*-indole (**3ah**): this compound was prepared employing phenylacetylene **1a** (109 µL, 102 mg, 1.00 mmol) and (4-bromophenyl)hydrazine hydrochloride **2h** (223.5 mg, 1.00 mmol) in a yield of 216 mg (0.80 mmol, 80%). Purification was performed by column chromatography (EtOAc/Hexane = 1:4). ^1^H NMR (400 MHz, DMSO-*d*_6_) δ 11.77 (s, 1H), 7.86 (d, *J* = 7.7 Hz, 2H), 7.72 (s, 1H), 7.48 (t, *J* = 7.5 Hz, 2H), 7.36 (t, *J* = 7.1 Hz, 2H), 7.21 (d, *J* = 8.5 Hz, 1H), 6.90 (s, 1H); ^13^C{^1^H} NMR (101 MHz, DMSO-*d*_6_) δ 139.2, 135.8, 131.7, 130.6, 129.1 (2C), 128.0, 125.2 (2C), 124.0, 122.2, 113.3, 111.9, 98.3. All of the spectral characteristics were identical to those described previously [76].

2-Phenyl-1*H*-indole-5-carboxamide (**3ai**): this compound was prepared employing phenylacetylene **1a** (109 µL, 102 mg, 1.00 mmol) and 4-hydrazino-benzamide hydrochloride **2i** (187.5 mg, 1.00 mmol) in a yield of 170 mg (0.72 mmol, 72%). Purification was performed by column chromatography (EtOAc/Hexane =1:4). The titled compound was obtained as gray solid, m.p. 234–236 °C (EtOH), R*_f_* 0.33 (EtOAc/Hexane, 1:2, *v*/*v*). ^1^H NMR (400 MHz, DMSO-*d*_6_) δ 11.81 (s, 1H), 8.14 (s, 1H), 7.93–7.84 (m, 3H), 7.67 (dd, *J* = 8.5, 1.4 Hz, 1H), 7.47 (t, *J* = 7.7 Hz, 2H), 7.41 (d, *J* = 8.5 Hz, 1H), 7.33 (t, *J* = 7.3 Hz, 1H), 7.13 (s, 1H), 7.00 (s, 1H); ^13^C{^1^H} NMR (101 MHz, DMSO-*d*_6_) δ 169.2, 139.1, 138.9, 131.9, 129.1 (2C), 128.1, 127.9, 125.8, 125.2 (2C), 121.6, 120.4, 110.9, 99.8; IR, *v_max_*: 3336, 3192, 1658, 1551, 1424, 1278, 1011, 949 cm^−1^; HRMS (ESI TOF) *m*/*z* calcd. for C_15_H_12_N_2_NaO [M + Na]^+^: 259.0842, found: 259.0841 (0.4 ppm).

2-(2-Naphthyl)-1*H*-indole (**3ba**): this compound was prepared employing (2-naphthyl)acetylene **1b** (152 mg, 1.00 mmol) and phenylhydrazine **2a** (109.0 mg, 1.00 mmol) in a yield of 197 mg (0.81 mmol, 81%). Purification was performed by column chromatography (EtOAc/Hexane = 1:4). ^1^H NMR (400 MHz, CDCl_3_) δ 8.45 (s, 1H), 8.05 (s, 1H), 7.98–7.76 (m, 4H), 7.69 (d, *J* = 7.5 Hz, 1H), 7.48 (dd, *J* = 21.7, 6.5 Hz, 3H), 7.25 (d, *J* = 5.8 Hz, 1H), 7.17 (t, *J* = 7.0 Hz, 1H), 6.97 (s, 1H); ^13^C{^1^H} NMR (101 MHz, CDCl_3_) δ 137.9, 137.1, 133.7, 132.9, 129.7, 129.4, 128.9, 128.1, 127.9, 126.8, 126.2, 123.9, 123.2, 122.7, 120.8, 120.4, 111.1, 100.8. All of the spectral characteristics were identical to those described previously [76].

5-Methyl-2-(naphthalen-2-yl)-1*H*-indole (**3bc**): this compound was prepared employing (2-naphthyl)acetylene **1b** (152 mg, 1.00 mmol) and *p*-tolyl hydrazine hydrochloride **2c** (158.5 mg, 1.00 mmol) in a yield of 190 mg (0.74 mmol, 74%). Purification was performed by column chromatography (EtOAc/Hexane =1:4). The titled compound was obtained as white solid, m.p. 216–218 °C (EtOH), R*_f_* 0.53 (EtOAc/Hexane, 1:10, *v*/*v*). ^1^H NMR (400 MHz, DMSO-*d*_6_) δ 11.57 (s, 1H), 8.35 (s, 1H), 8.04–7.95 (m, 2H), 7.95–7.89 (m, 2H), 7.59–7.46 (m, 2H), 7.32 (d, *J* = 9.6 Hz, 2H), 6.95 (d, *J* = 8.0 Hz, 2H), 2.38 (s, 3H); ^13^C{^1^H} NMR (101 MHz, DMSO-*d*_6_) δ 137.6, 135.8, 133.3, 132.2, 129.8, 129.0, 128.4, 127.92, 127.87, 127.7, 126.7, 126.0, 123.8, 123.5, 122.7, 119.7, 111.1, 99.1, 21.3; IR, *v_max_*: 3431, 2922, 1758, 1407, 1243, 1050 cm^−1^; HRMS (ESI TOF) *m*/*z* calcd. for C_19_H_14_N [M − H]^−^: 256.1132, found: 256.1132 (0.1 ppm).

2-(3,4-Dimethylphenyl)-5-fluoro-1*H*-indole (**3bf**): this compound was prepared employing (2-naphthyl)acetylene **1b** (152 mg, 1.00 mmol) and (4-fluorophenyl) hydrazine hydrochloride **2f** (162.5 mg, 1.00 mmol) in a yield of 198 mg (0.76 mmol, 76%). Purification was performed by column chromatography (EtOAc/Hexane = 1:4). The titled compound was obtained as gray solid, m.p. 205–207 °C (EtOH), R*_f_* 0.6 (EtOAc/Hexane, 1:4, *v*/*v*). ^1^H NMR (400 MHz, DMSO-*d*_6_) δ 11.85 (s, 1H), 8.39 (s, 1H), 8.03–7.97 (m, 2H), 7.97–7.89 (m, 2H), 7.58–7.53 (m, 1H), 7.53–7.48 (m, 1H), 7.44 (dd, *J* = 8.7, 4.6 Hz, 1H), 7.32 (dd, *J* = 9.9, 2.5 Hz, 1H), 7.03 (d, *J* = 1.7 Hz, 1H), 6.98 (td, *J* = 9.3, 2.5 Hz, 1H); ^13^C{^1^H} NMR (101 MHz, DMSO-*d*_6_) δ 157.3 (d, *J* = 231.7 Hz), 139.6, 134.1, 133.3, 132.5, 129.4, 129.0 (d, *J* = 10.5 Hz), 128.6, 128.0, 127.8, 126.8, 126.3, 123.8, 123.2, 112.3 (d, *J* = 9.8 Hz), 110.0 (d, *J* = 26.1 Hz), 104.7 (d, *J* = 23.2 Hz), 99.7 (d, *J* = 4.8 Hz); IR, *v_max_*: 3079, 1443, 1347, 1301, 1224, 1046, 817 cm^−1^; HRMS (ESI TOF) *m*/*z* calcd. for C_18_H_13_FN [M + H]^+^: 262.1027, found: 262.1026 (0.1 ppm).

5-Chloro-2-(naphthalen-2-yl)-1H-indole (**3bg**): this compound was prepared employing (2-naphthyl)acetylene **1b** (152 mg, 1.00 mmol) and (4-chlorophenyl)hydrazine hydrochloride **2g** (179.0 mg, 1.00 mmol) in a yield of 213 mg (0.77 mmol, 77%). Purification was performed by column chromatography (EtOAc/Hexane = 1:4). The titled compound was obtained as white solid, m.p. 191–194 °C (EtOH), R*_f_* 0.41 (EtOAc/Hexane, 1:10, *v*/*v*). ^1^H NMR (400 MHz, DMSO-*d*_6_) δ 11.93 (s, 1H), 8.39 (s, 1H), 8.01 (s, 2H), 7.94 (t, *J* = 6.8 Hz, 2H), 7.60 (d, *J* = 2.2 Hz, 1H), 7.59–7.49 (m, 2H), 7.44 (d, *J* = 8.6 Hz, 1H), 7.12 (dd, *J* = 8.6, 2.1 Hz, 1H), 7.04 (s, 1H); ^13^C{^1^H} NMR (101 MHz, DMSO-*d*_6_) δ 139.3, 135.8, 133.2, 132.5, 129.9, 129.2, 128.6, 128.0, 127.8, 126.8, 126.3, 124.0, 123.8, 123.3, 121.7, 119.2, 112.8, 99.2; IR, *v_max_*: 3442, 1670, 1597, 1499, 1089 cm^−1^; HRMS (ESI TOF) *m*/*z* calcd. for C_18_H_11_ClN [M − H]^−^: 276.0586, found: 276.0589 (−1.2 ppm).

2,3-Diphenyl-1*H*-indole (**3ca**): this compound was prepared employing 1,2-diphenylacetylene **1c** (178 mg, 1.00 mmol) and phenylhydrazine **2a** (109.0 mg, 1.00 mmol) in a yield of 164 mg (0.61 mmol, 61%). Purification was performed by column chromatography (EtOAc/Hexane = 1:4). ^1^H NMR (400 MHz, CDCl_3_) δ 8.21 (s, 1H), 7.75 (d, *J* = 7.9 Hz, 1H), 7.50 (d, *J* = 7.1 Hz, 2H), 7.48–7.41 (m, 5H), 7.39–7.28 (m, 5H), 7.22 (t, *J* = 7.5 Hz, 1H); ^13^C{^1^H} NMR (101 MHz, CDCl_3_) δ 136.0, 135.2, 134.2, 132.8, 130.3 (2C), 128.83, 128.79 (2C), 128.7 (2C), 128.3 (2C), 127.8, 126.3, 122.8, 120.5, 119. 8, 115.1, 111.0. All of the spectral characteristics were identical to those described previously [77].

### 3.3. Preparation of Acetophenones ***7*** (General Procedure)

A 5 mL round-bottom flask equipped with a magnetic stir bar was charged with acetylene **1** (1.00 mmol) and polyphosphoric acid (2 g, P_2_O_5_ 80 w.t. %) and stirred upon heating at 100 °C for 30 min (TLC control). After the reaction was complete, the mixture was poured into 80 mL of cold water and basified with 20% ammonia solution. After extraction with EtOAc (4 × 20 mL), the organic fraction was concentrated in vacuo, and the crude material was purified by column chromatography (EtOAc/Hexane).

Acetophenone (**7a**): this compound was prepared employing phenylacetylene **1a** (109 µL, 102 mg, 1.00 mmol) in a yield of 105 mg (0.88 mmol, 88%). Purification was performed by column chromatography (EtOAc/Hexane = 1:4). ^1^H NMR (400 MHz, DMSO-*d*_6_) δ 7.95 (d, *J* = 7.9 Hz, 2H), 7.62 (t, *J* = 7.3 Hz, 1H), 7.51 (t, *J* = 7.2 Hz, 2H), 2.57 (s, 2H); ^13^C{^1^H} NMR (101 MHz, DMSO-*d*_6_) δ 198.0, 136.9, 133.2, 128.7 (2C), 128.2 (2C), 26.7. All of the spectral characteristics were identical to those described previously [78].

2-Acetylnaphtalene (**7b**): this compound was prepared employing 2-ethynylnaphthalene **1b** (152 mg, 1.00 mmol) in a yield of 143 mg (0.84 mmol, 84%). Purification was performed by column chromatography (EtOAc/Hexane = 1:4). ^1^H NMR (400 MHz, DMSO-*d*_6_) δ 8.66 (s, 1H), 8.12 (d, *J* = 8.0 Hz, 1H), 8.05–7.94 (m, 3H), 7.66 (ddd, *J* = 8.1, 6.8, 1.5 Hz, 1H), 7.62 (ddd, *J* = 8.2, 6.9, 1.5 Hz, 1H), 2.70 (s, 3H); ^13^C{^1^H} NMR (101 MHz, DMSO-*d*_6_) δ 198.4, 135.5, 134.6, 132.7, 130.8, 130.1, 129.1, 128.7, 128.1, 127.4, 124.0, 27.2. All of the spectral characteristics were identical to those described previously [79].

Phenylbenzylketone (**7c**): this compound was prepared employing 1,2-diphenylacetylene **1c** (178 mg, 1.00 mmol) in a yield of 169 mg (0.86 mmol, 86%). Purification was performed by column chromatography (EtOAc/Hexane = 1:4). ^1^H NMR (400 MHz, CDCl_3_) δ 8.10–7.97 (m, 2H), 7.62–7.53 (m, 1H), 7.51–7.43 (m, 2H), 7.40–7.33 (m, 2H), 7.29 (dt, *J* = 8.0, 2.0 Hz, 3H), 4.31 (s, 2H); ^13^C{^1^H} NMR (101 MHz, CDCl_3_) δ 197.8, 136.7, 134.6, 133.3, 129.6 (2C), 128.8 (2C), 128.8 (2C), 128.7 (2C), 127.0, 45.6. All of the spectral characteristics were identical to those described previously [80].

2-(2-Oxopropyl)isoindoline-1,3-dione (**7d**): this compound was prepared employing 2-(prop-2-yn-1-yl)isoindoline-1,3-dione **1d** [68] (185 mg, 1.00 mmol) in a yield of 158 mg (0.78 mmol, 78%). Purification was performed by column chromatography (EtOAc/Hexane = 1:4). ^1^H NMR (400 MHz, CDCl_3_) δ 7.87 (dd, *J* = 5.5, 3.1 Hz, 2H), 7.74 (dd, *J* = 5.5, 3.1 Hz, 2H), 4.50 (s, 2H), 2.27 (s, 3H); ^13^C{^1^H} NMR (101 MHz, CDCl_3_) δ 199.8 (2C), 167.8 (2C), 134.3 (2C), 132.2 (2C), 123.7 (2C), 47.3, 27.1. All of the spectral characteristics were identical to those described previously [81].

1-(1*H*-benzo[d]imidazol-1-yl)propan-2-one (**7e**): this compound was prepared employing 1-(prop-2-yn-1-yl)-1*H*-benzo[*d*]imidazole **1e** [69] (156 mg, 1.00 mmol) in a yield of 115 mg (0.74 mmol, 74%). Purification was performed by column chromatography (EtOAc/Hexane = 1:4). ^1^H NMR (400 MHz, CDCl_3_) δ 7.94 (s, 1H), 7.87–7.76 (m, 1H), 7.30 (dd, *J* = 5.4, 3.2 Hz, 2H), 7.24–7.15 (m, 1H), 4.92 (s, 2H), 2.18 (s, 3H); ^13^C{^1^H} NMR (101 MHz, CDCl_3_) δ 201.1, 143.4, 143.2 (2C), 133.9, 123.7, 122.8, 120.5 (2C), 109.3 (2C), 54.0, 27.1. All of the spectral characteristics were identical to those described previously [82].

1-Phenyl-2-(pyrimidin-5-yl)ethan-1-one (**7f**): this compound was prepared employing 5-(phenylethynyl)pyrimidine **1f** [70] (180 mg, 1.00 mmol) in a yield of 160 mg (0.81 mmol, 81%). Purification was performed by column chromatography (EtOAc/Hexane = 1:4). The titled compound was obtained as yellow solid, m.p. 61–62 °C (EtOAc/Hexane), R*_f_* 0.2 (EtOAc/Hexane, 1:2, *v*/*v*). ^1^H NMR (400 MHz, CDCl_3_) δ 9.15 (s, 1H), 8.67 (s, 2H), 8.07–7.99 (m, 2H), 7.68–7.59 (m, 1H), 7.55–7.48 (m, 2H), 4.32 (s, 2H); ^13^C{^1^H} NMR (101 MHz, CDCl_3_) δ 195.1, 158.0 (2C), 157.4, 136.0, 134.1, 129.1 (2C), 128.5 (2C), 128.4, 39.8; IR, *v_max_*: 3098, 3021, 1726, 1579, 1410, 1258, 1051 cm^−1^; HRMS (ESI TOF) *m*/*z* calcd. for C_12_H_10_N_2_NaO [M + Na]^+^: 221.0685, found: 221.0686 (−0.1 ppm).

4-Methoxyacetophenone (**7h**): this compound was prepared employing (4-methoxyphenyl)acetylene **1h** (132 mg, 1.00 mmol) in a yield of 128 mg (0.85 mmol, 85%). Purification was performed by column chromatography (EtOAc/Hexane = 1:4). ^1^H NMR (400 MHz, CDCl_3_) δ 8.08–7.75 (m, 2H), 7.01–6.79 (m, 2H), 3.85 (s, 3H), 2.54 (s, 3H); ^13^C{^1^H} NMR (101 MHz, CDCl_3_) δ 196.9, 163.6, 130.7 (2C), 130.4, 113.7 (2C), 55.6, 26.5. All of the spectral characteristics were identical to those described previously [79].

4-Chloroacetophenone (**7i**): this compound was prepared employing (4-chlorophenyl)acetylene **1i** (136 mg, 1.00 mmol) in a yield of 140 mg (0.91 mmol, 91%). Purification was performed by column chromatography (EtOAc/Hexane = 1:4). ^1^H NMR (400 MHz, CDCl_3_) δ 8.02–7.68 (m, 2H), 7.57–7.39 (m, 2H), 2.59 (s, 3H); ^13^C{^1^H} NMR (101 MHz, CDCl_3_) δ 197.0, 139.7, 135.5, 129.9 (2C), 129.0 (2C), 26.7. All of the spectral characteristics were identical to those described previously [79].

4-Bromoacetophenone (**7j**): this compound was prepared employing (4-bromophenyl)acetylene **1j** (180 mg, 1.00 mmol) in a yield of 176 mg (0.88 mmol, 88%). Purification was performed by column chromatography (EtOAc/Hexane = 1:4). ^1^H NMR (400 MHz, CDCl_3_) δ 7.85–7.76 (m, 2H), 7.64–7.58 (m, 2H), 2.58 (s, 2H); ^13^C{^1^H} NMR (101 MHz, CDCl_3_) δ 197.21, 135.92, 132.03, 129.98, 128.46, 26.71. All of the spectral characteristics were identical to those described previously [83].

2-Methyl-1-(2-oxopropyl)-1*H*-indole-3-carbaldehyde (**7k**): this compound was prepared employing 2-methyl-1-(prop-2-yn-1-yl)-1*H*-indole-3-carbaldehyde **1k** [84] (197 mg, 1.00 mmol) in a yield of 144 mg (0.67 mmol, 67%). Purification was performed by column chromatography (EtOAc/Hexane = 1:4). The titled compound was obtained as beige solid, m.p. 151–152 °C (EtOH), R*_f_* 0.15 (EtOAc/Hexane, 1:1, *v*/*v*). ^1^H NMR (400 MHz, DMSO-*d*_6_) δ 10.09 (s, 1H), 8.10 (dd, *J* = 5.7, 3.1 Hz, 1H), 7.46 (dd, *J* = 5.8, 3.3 Hz, 1H), 7.24–7.16 (m, 2H), 5.33 (s, 2H), 2.55 (s, 3H), 2.30 (s, 3H); ^13^C{^1^H} NMR (101 MHz, DMSO-*d*_6_) δ 202.6, 184.7, 149.8, 136.9, 124.9, 122.9, 122.5, 120.1, 113.8, 110.3, 52.5, 27.3, 10.0; IR, *v_max_*: 3073, 2763, 1790, 1703, 1542, 1436, 1244, 1143 cm^−1^; HRMS (ESI TOF) *m*/*z* calcd. for C_13_H_13_NNaO_2_ [M + Na]^+^: 238.0838, found: 238.0845 (−2.7 ppm).

(*E*)-1-(2-Methyl-3-(2-nitrovinyl)-1*H*-indol-1-yl)propan-2-one (**7l**): this compound was prepared employing (*E*)-2-methyl-3-(2-nitrovinyl)-1-(prop-2-yn-1-yl)-1*H*-indole **1l** [39] (240 mg, 1.00 mmol) in a yield of 191 mg (0.74 mmol, 74%). Purification was performed by column chromatography (EtOAc/Hexane = 1:4). The titled compound was obtained as orange solid, m.p. 250–251 °C (EtOH), R*_f_* 0.32 (EtOAc/Hexane, 1:1, *v*/*v*). ^1^H NMR (400 MHz, DMSO-*d*_6_) δ 8.35 (d, *J* = 13.2 Hz, 1H), 7.97 (d, *J* = 13.2 Hz, 1H), 7.90 (dd, *J* = 6.2, 2.7 Hz, 1H), 7.51 (dd, *J* = 6.2, 2.9 Hz, 1H), 7.28–7.21 (m, 2H), 5.36 (s, 2H), 2.47 (s, 3H), 2.30 (s, 3H); ^13^C{^1^H} NMR (101 MHz, DMSO-*d*_6_) δ 202.3, 148.6, 137.8, 133.2, 130.4, 124.5, 123.0, 122.4, 120.3, 110.7, 105.4, 53.0, 27.3, 10.4. IR, *v_max_*: 3099, 1793, 1711, 1603, 1456, 1311, 1131 cm^−1^; HRMS (ESI TOF) *m*/*z* calcd. for C_14_H_14_N_2_NaO_3_ [M + Na]^+^: 281.0897, found: 281.0904 (−2.6 ppm).

## 4. Conclusions

Herein, we demonstrated the synthetic practicality and efficiency of homogeneous, PPA-catalyzed hydroamination of simple alkynes with arylhydrazines, which, under the given protocol, leads to the formation of the corresponding Fischer indoles in good to excellent yields. In addition, we showed, for the first time, that acetylenes, like nitriles, can undergo hydrolysis in polyphosphoric acids, thereby, acting as a masked carbonyl group for further transformations.

## Figures and Tables

**Table 1:** Screening of reaction parameters for the synthesis of the 2-phenylindole **3aa**.

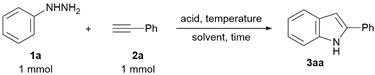					
#	Acid, g	Temperature, °C	Solvent	Time, h	Yield, ^a^ %
1	PPA 80%, 2	90		0.5	88
2	PPA 80%, 2	100		0.5	90
3	PPA 80%, 2	110		0.5	93
4	PPA 80%, 1	110		1	85
5	PPA 80%, 4	110		0.5	92
6	PPA 87%, 2	100		1	50
7	H_3_PO_4_, 1 mL	100		3	65
8	H_3_PO_4_, 0.5 mL	reflux	EtOH (1 mL)	3	33
9	PPA 80%, 2	reflux	Toluene (1 mL)	1.5	91

^a^ All reactions were performed on 1 mmol scales and equimolar quantities of reactants. Isolated yields of purified materials are provided.

**Table 2:** Screening of reaction parameters for PPA-assisted hydrolysis of phenylacetylene **1a**.

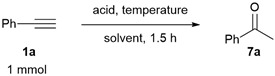				
#	Acid, g	Temperature, °C	Solvent	Yield ^a^, %
1	PPA 80%, 2	90		88
2	PPA 80%, 2	100		90
3	PPA 80%, 2	110		93
4	PPA 80%, 1	110		85
5	PPA 80%, 4	110		92
6	PPA 87%, 2	100		50
7	H_3_PO_4_, 2	100		65
8	H_3_PO_4_, 0.5	reflux	EtOH (1 mL)	33
9	PPA 80%, 2	reflux	Toluene (1 mL)	91

^a^ All reactions were performed on 1 mmol scales. Isolated yields of purified materials are provided.

## Data Availability

The supporting information includes NMR and HRMS spectral charts.

## Supplementary Materials

The following supporting information can be downloaded at: https://www.mdpi.com/article/10.3390/ijms25168750/s1.
