# Supplementary material for: Metal-Free, PPA-Mediated Fisher Indole Synthesis via Tandem Hydroamination–Cyclization Reaction between Simple Alkynes and Arylhydrazines
Source: Int J Mol Sci. 2024 Aug 11;25(16):8750. doi: 10.3390/ijms25168750 (PMC11354626; doi:10.3390/ijms25168750)
Supplement: Supplementary file 1 [file ijms-25-08750-s001.zip › ijms-3139253-supplementary.pdf]

Electronic Supplementary Material (ESI)

Metal-Free, PPA-Mediated Fisher Indole Synthesis via Tandem, Hydroamination-Cyclization Reaction between Simple Alkynes and Arylhydrazines

Alexander V. Aksenov*, Dinara C. Makieva, Rodion A. Arestov, Nikolai A. Arutiunov, Dmitrii A. Aksenov, Nicolai A. Aksenov, Alexander V. Leontiev and Inna V. Aksenova

Department of Chemistry, North Caucasus Federal University, 1a Pushkin St., 355017 Stavropol, Russia

*****corresponding author: aaksenov@ncfu.ru

^{1}H and ^{13}C NMR spectral charts.. S2

^{1}H and ^{13}C NMR spectral charts for indoles **3**.. S2

^{1}H and ^{13}C NMR spectral charts for acetophenones **7**.. S26

HRMS spectral charts... S48

HRMS spectral charts for indoles **3**.. S48

HRMS charts for acetophenones **7**.. S50

^{1}H and ^{13}C NMR spectral charts for indoles **3**

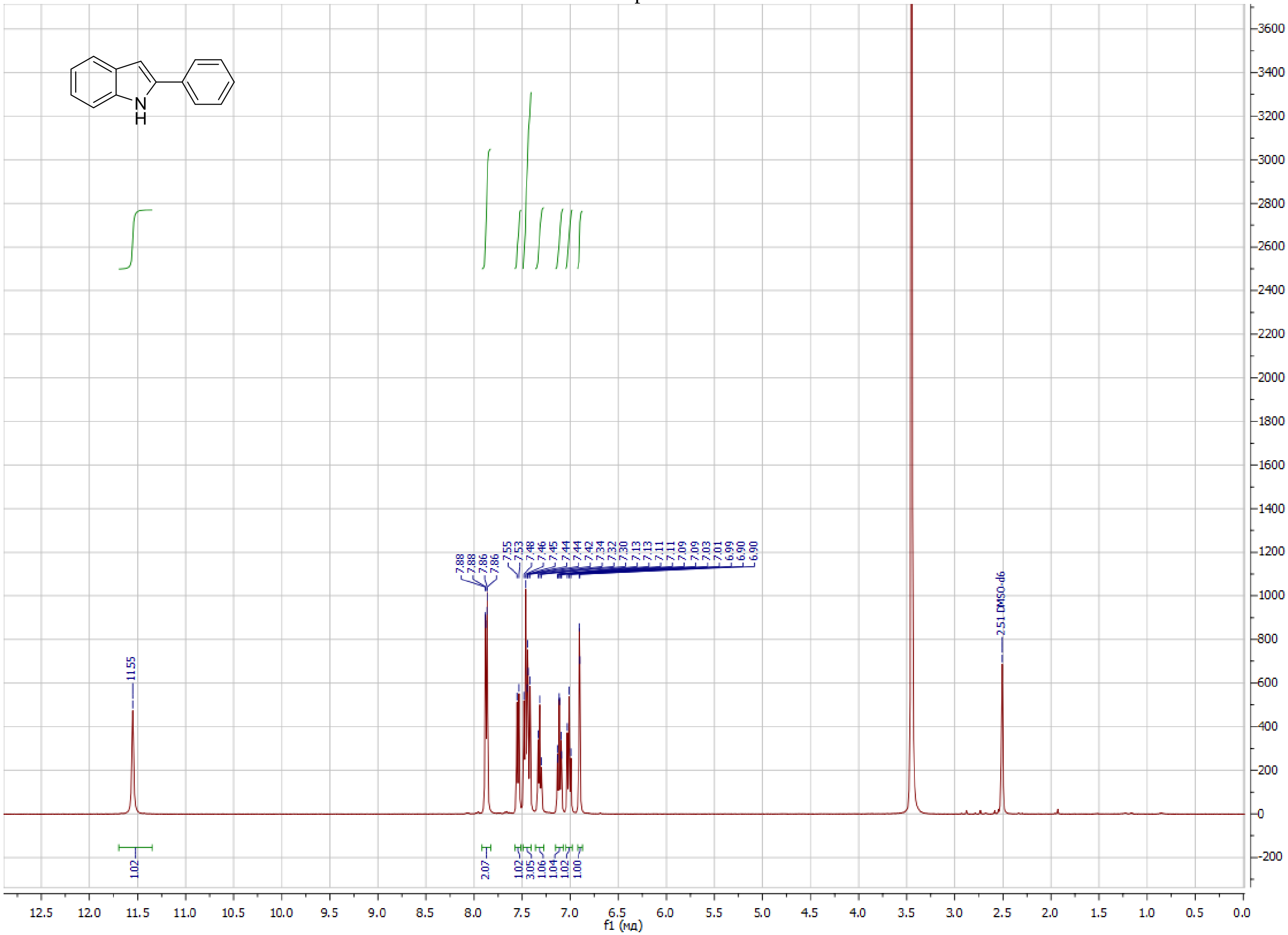

Figure S1. ^{1}H NMR spectrum of indole **3aa** in DMSO-d_6 (400 MHz)

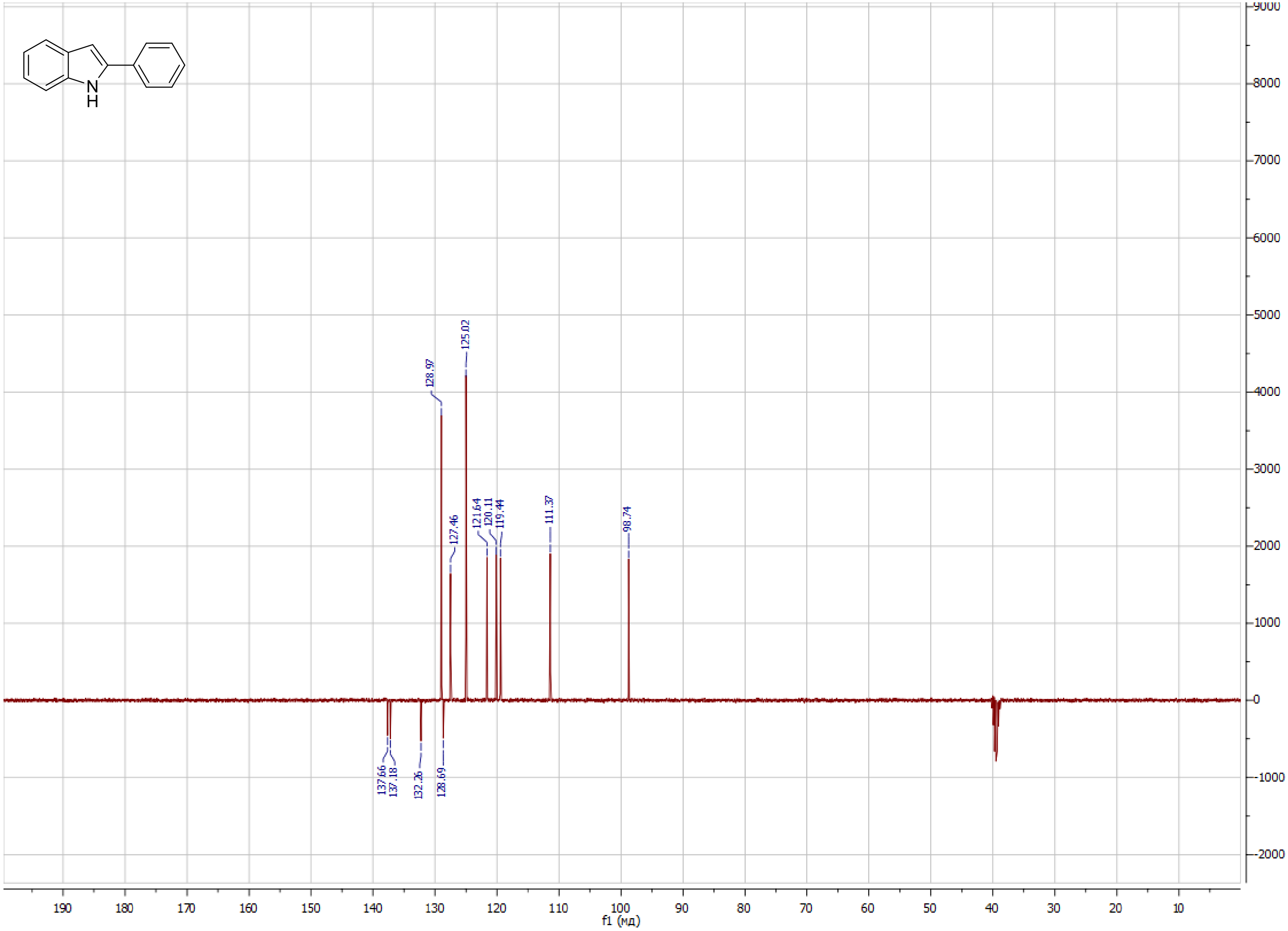

Figure S2. $^{13}C\{^1H\}$ NMR spectrum of indole **3aa** in DMSO-d_6 (100 MHz)

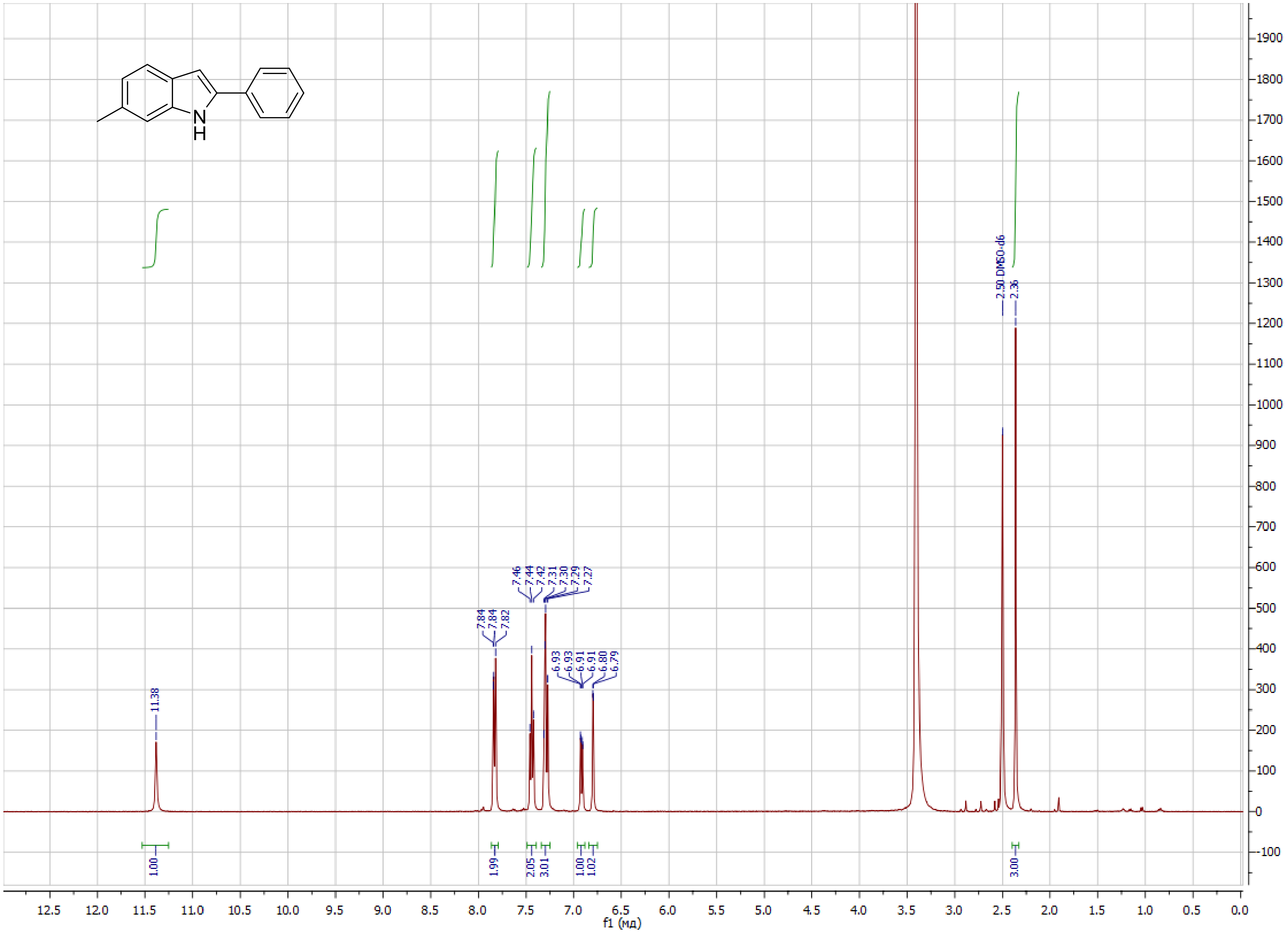

Figure S3. ^{1}H NMR spectrum of indole **3ab** in DMSO-d_6 (400 MHz)

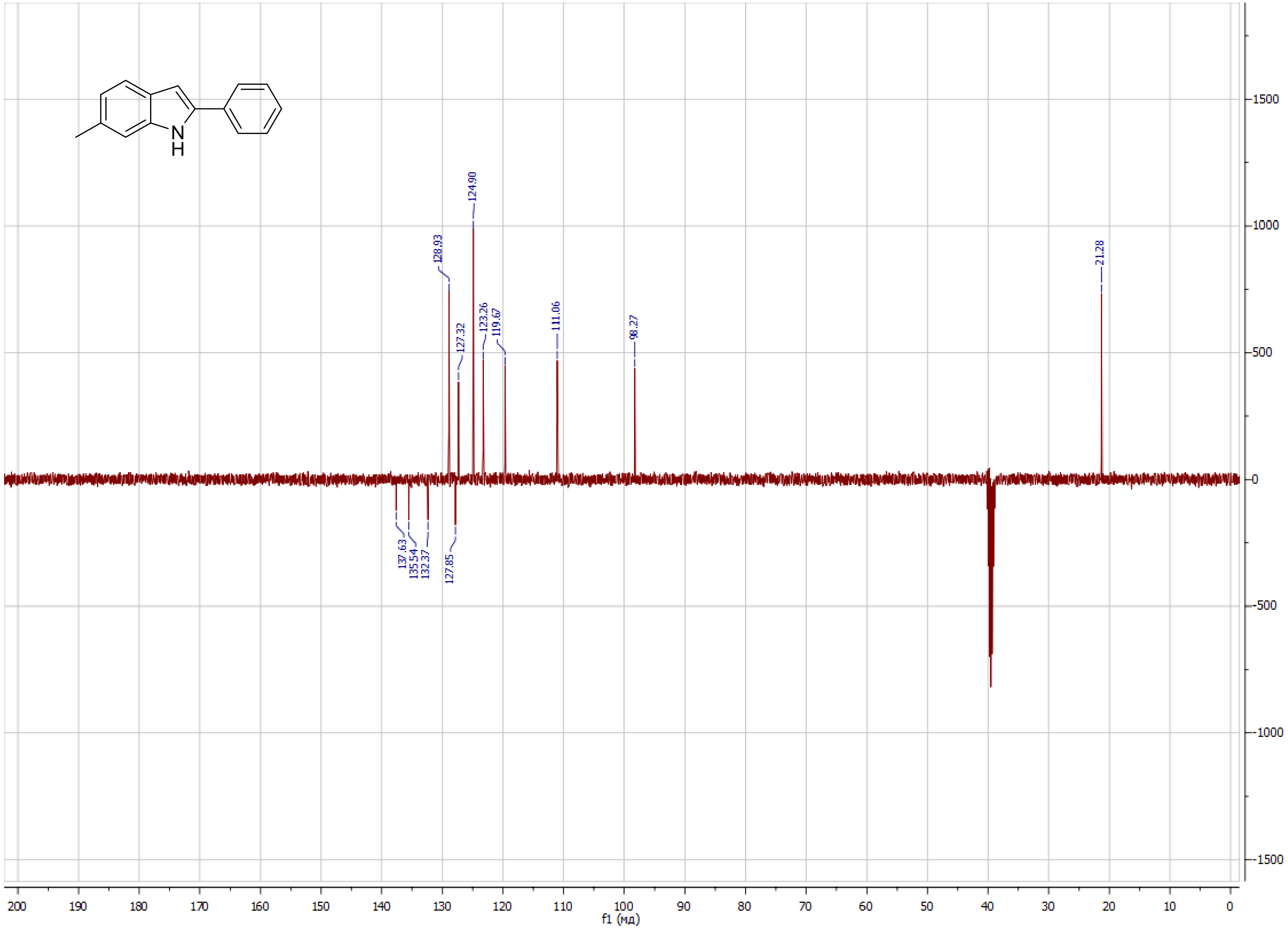

Figure S4. $^{13}C\{^{1}H\}$ NMR spectrum of indole **3ab** in DMSO-d_6 (100 MHz)

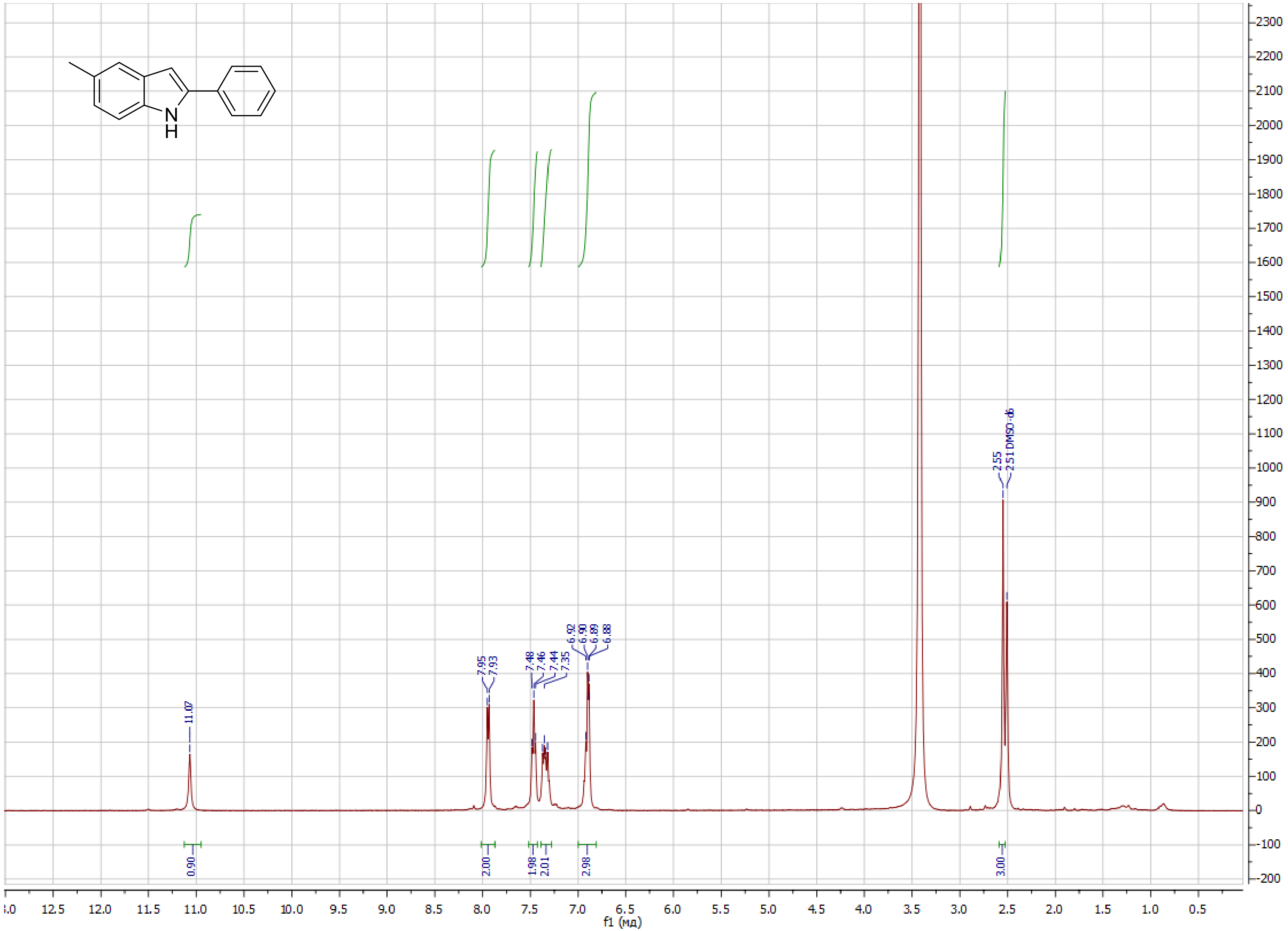

Figure S5. ^{1}H NMR spectrum of indole **3ac** in DMSO-d_6 (400 MHz)

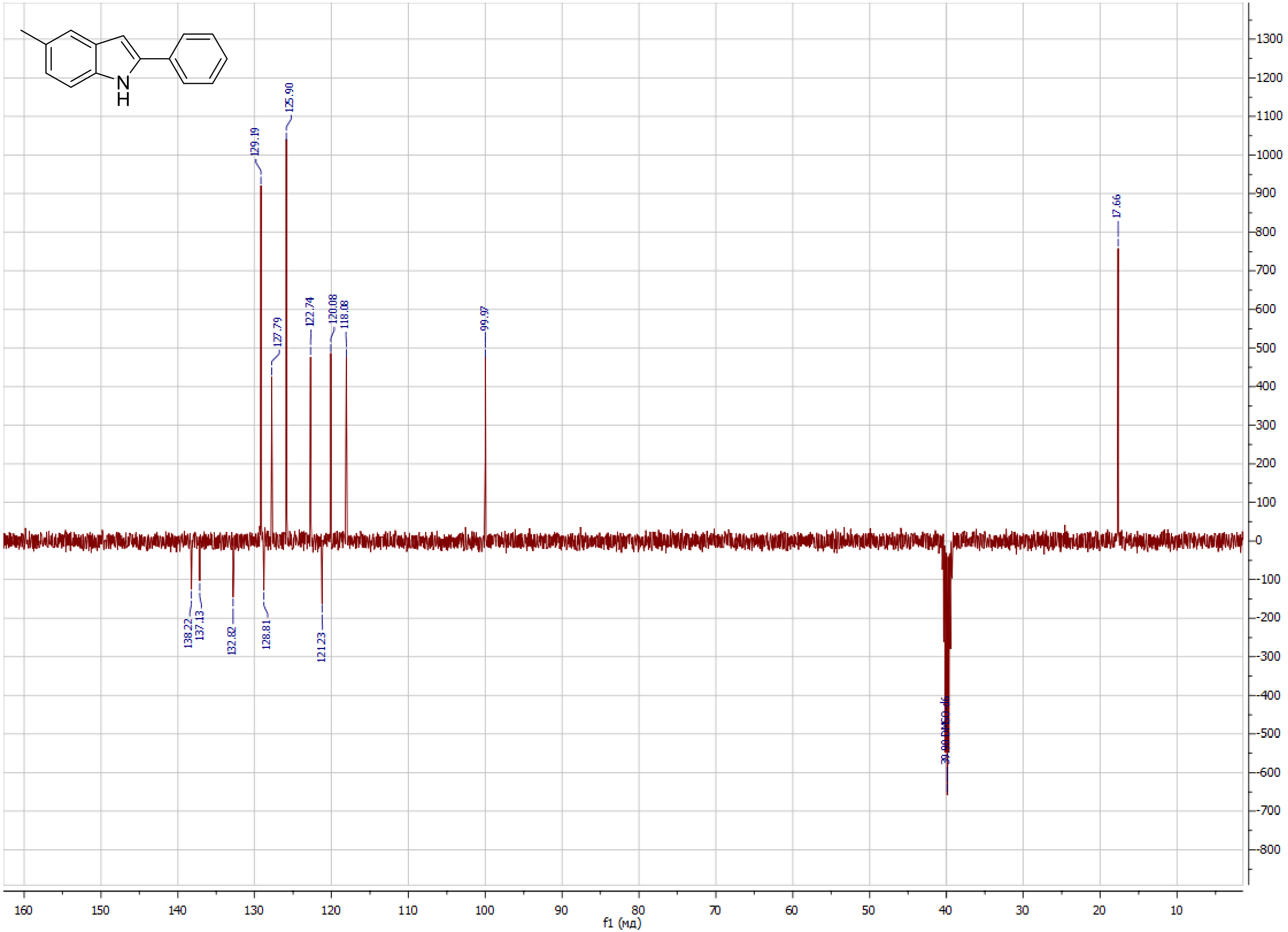

Figure S6. $^{13}C\{^{1}H\}$ NMR spectrum of indole **3ac** in DMSO-d_6 (100 MHz)

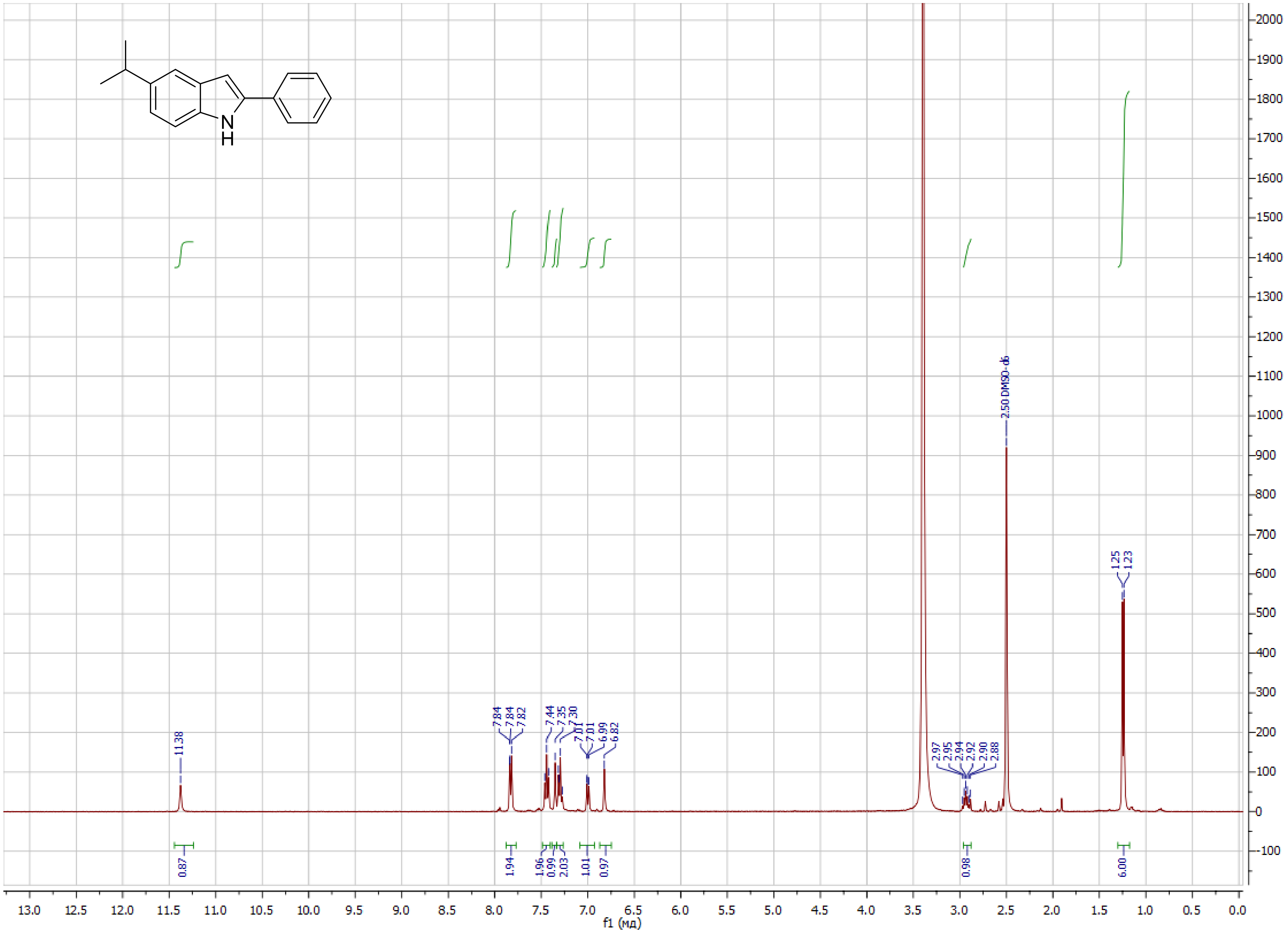

Figure S7. ^{1}H NMR spectrum of indole **3ad** in DMSO-d_6 (400 MHz)

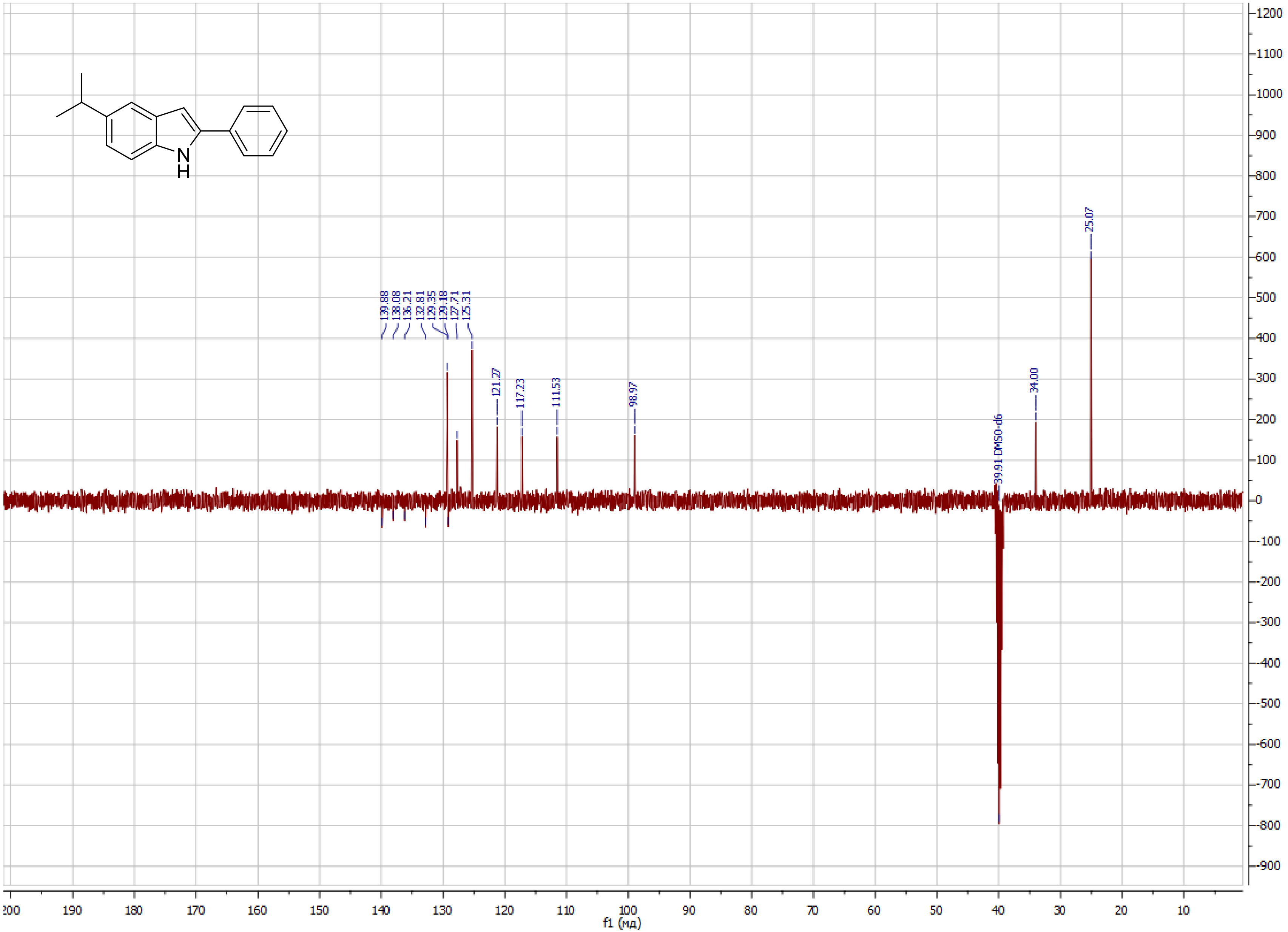

Figure S8. $^{13}C\{^{1}H\}$ NMR spectrum of indole **3ad** in DMSO-d_6 (100 MHz)

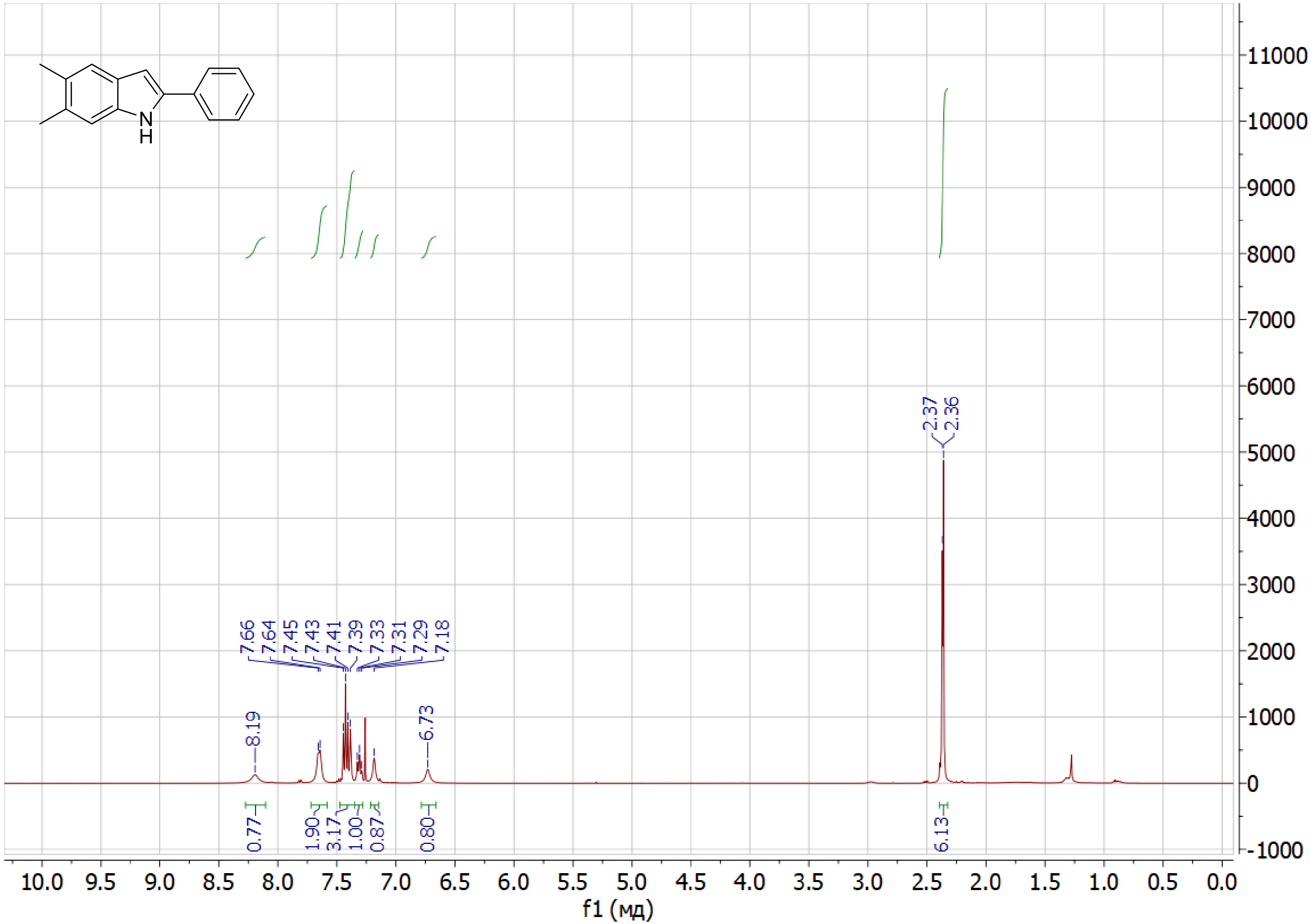

Figure S9. ^{1}H NMR spectrum of indole **3ae** in $CDCl_3$ (400 MHz)

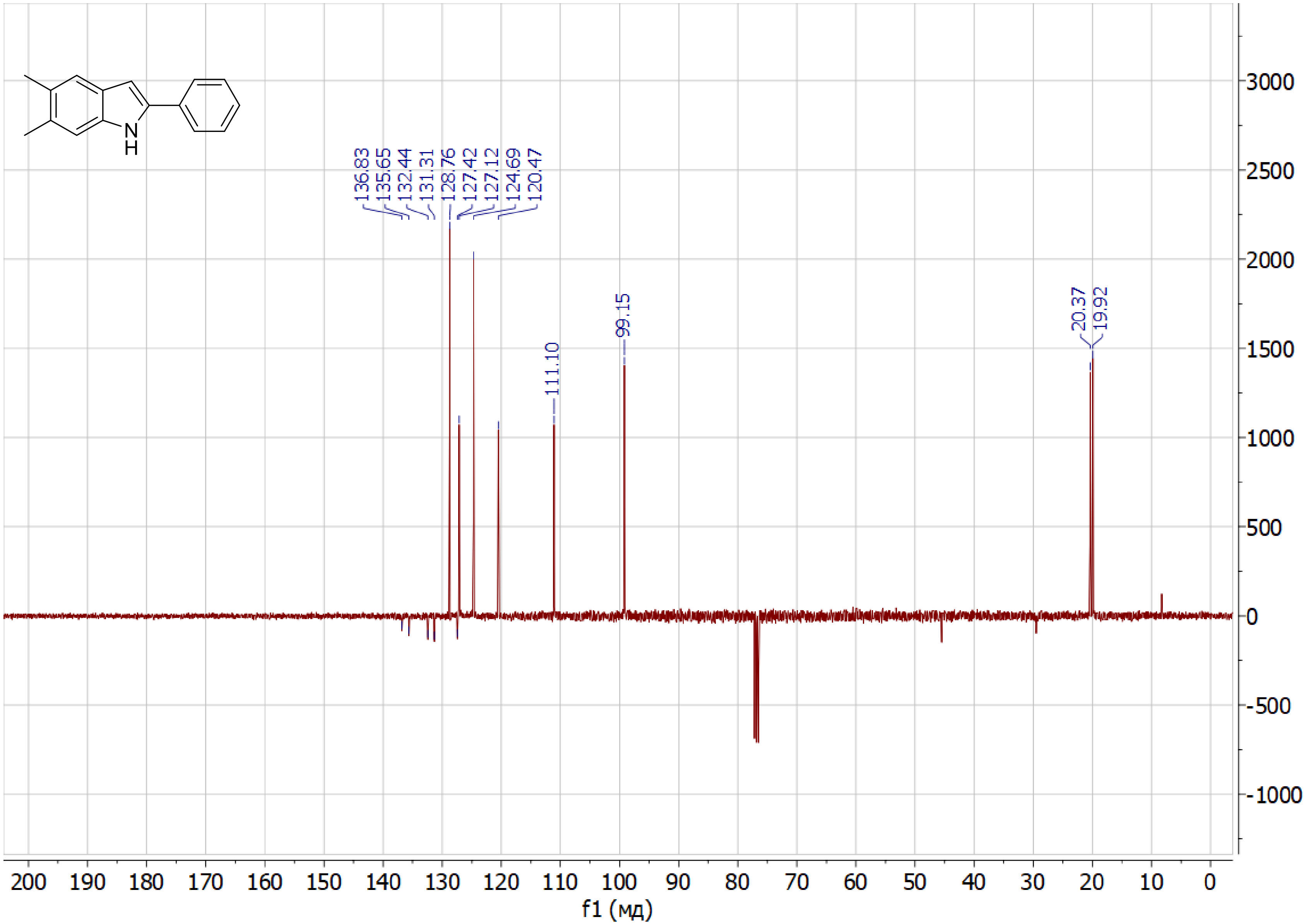

Figure S10. $^{13}C\{^1H\}$ NMR spectrum of indole **3ae** in $CDCl_3$ (100 MHz)

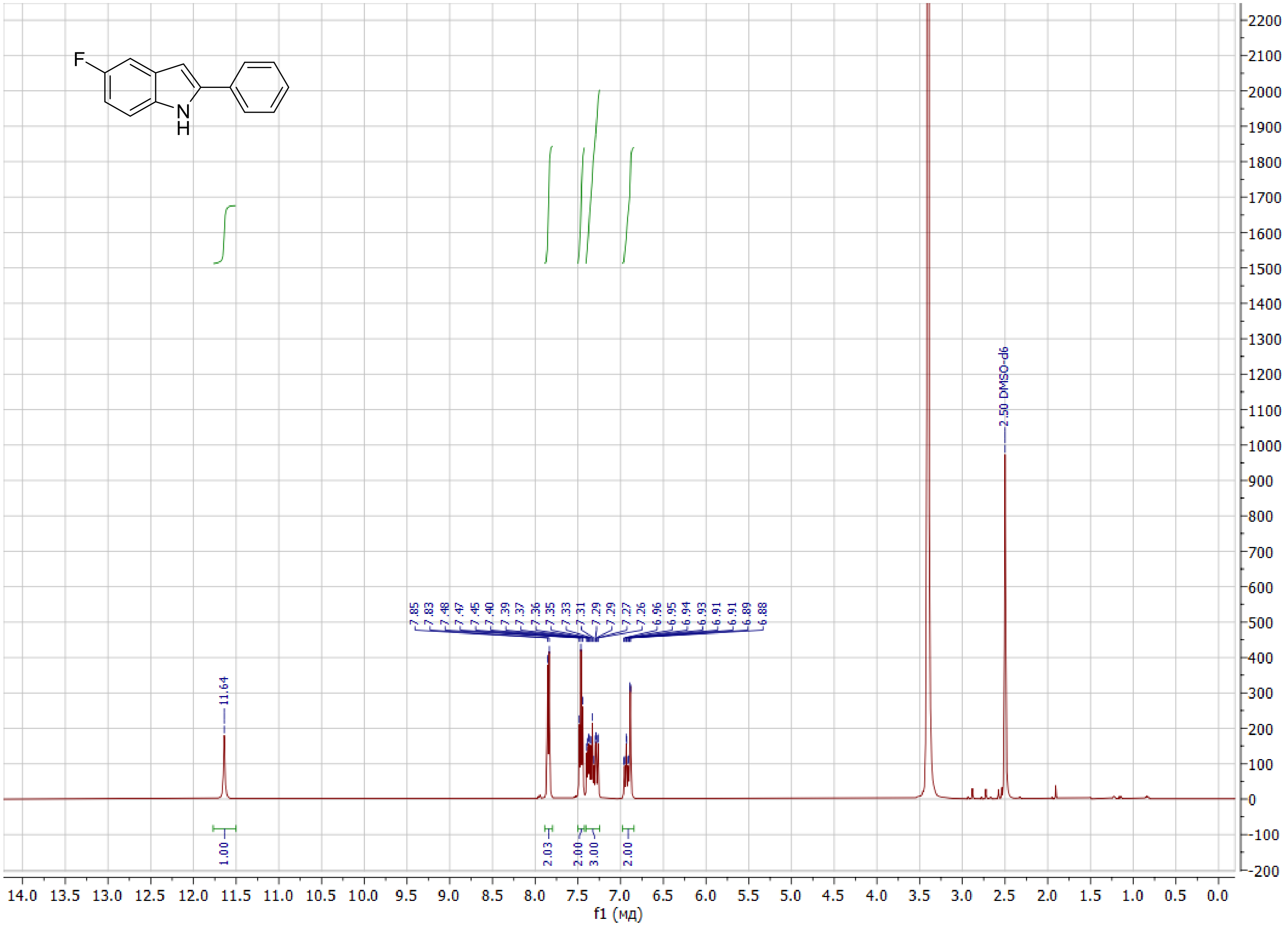

Figure S11. ^{1}H NMR spectrum of indole **3af** in DMSO-d_6 (400 MHz)

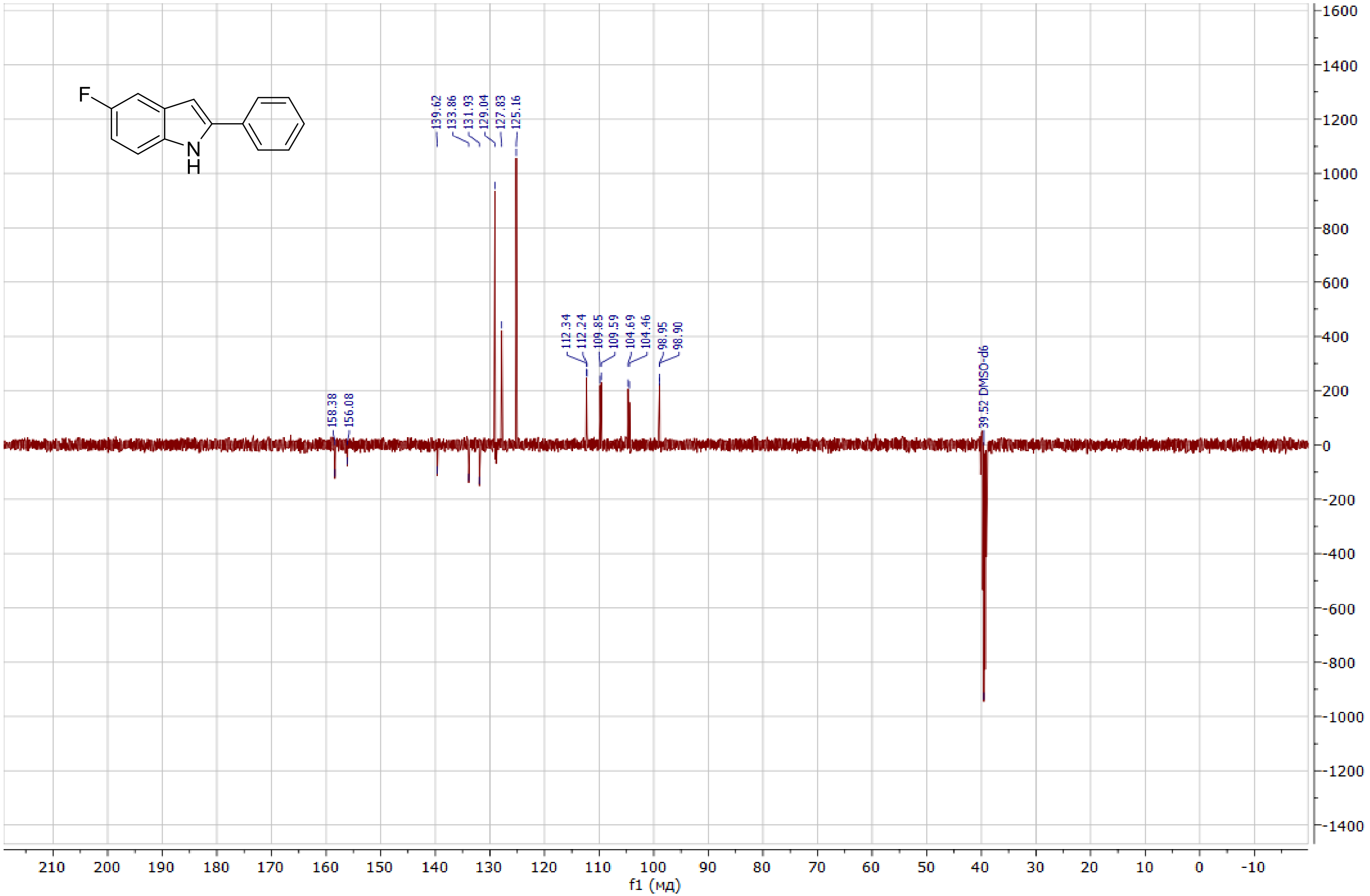

Figure S12. $^{13}C\{^1H\}$ NMR spectrum of indole **3af** in DMSO-d_6 (100 MHz)

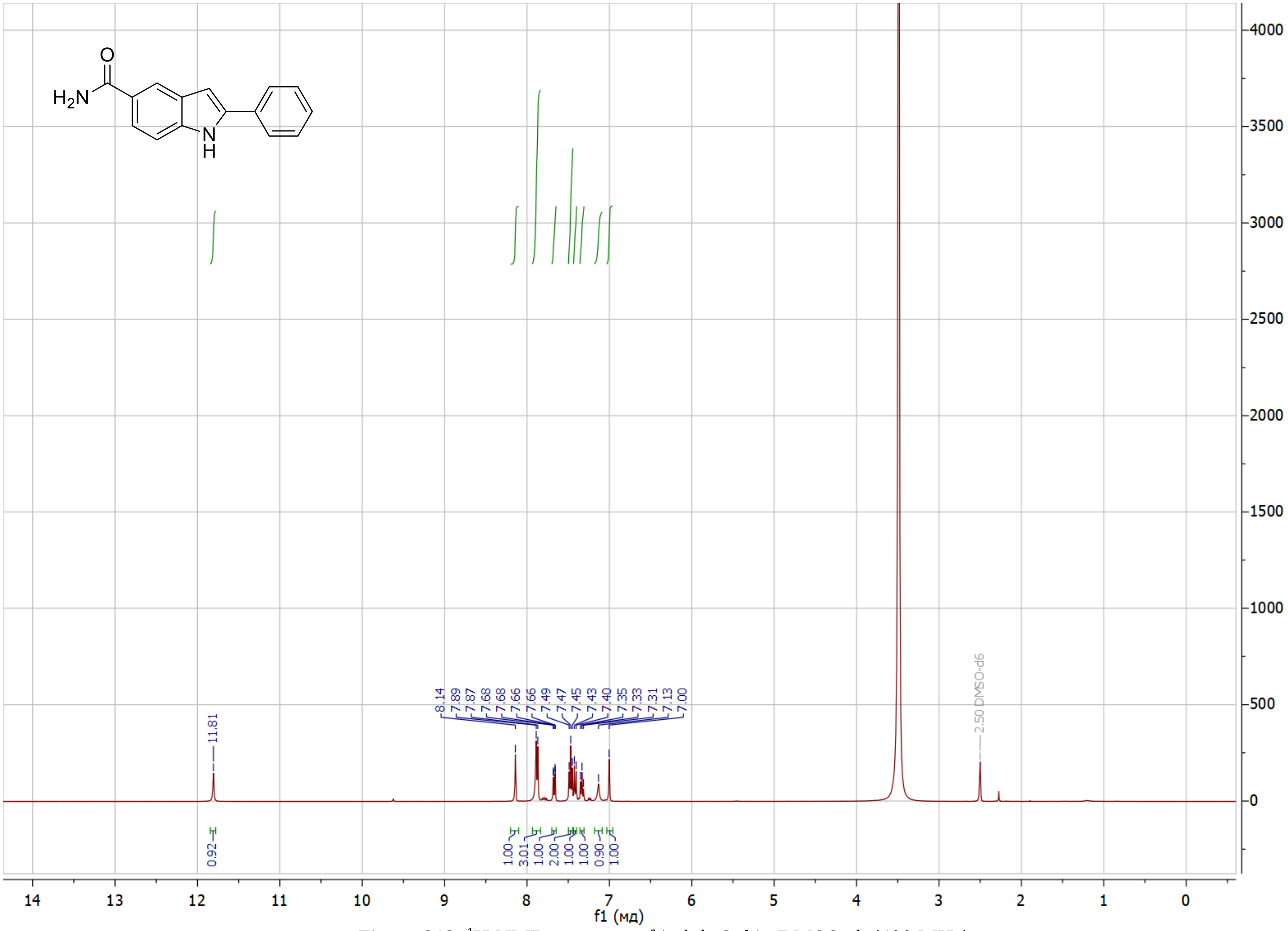

Figure S13. ^{1}H NMR spectrum of indole **3ai** in DMSO-d_6 (400 MHz)

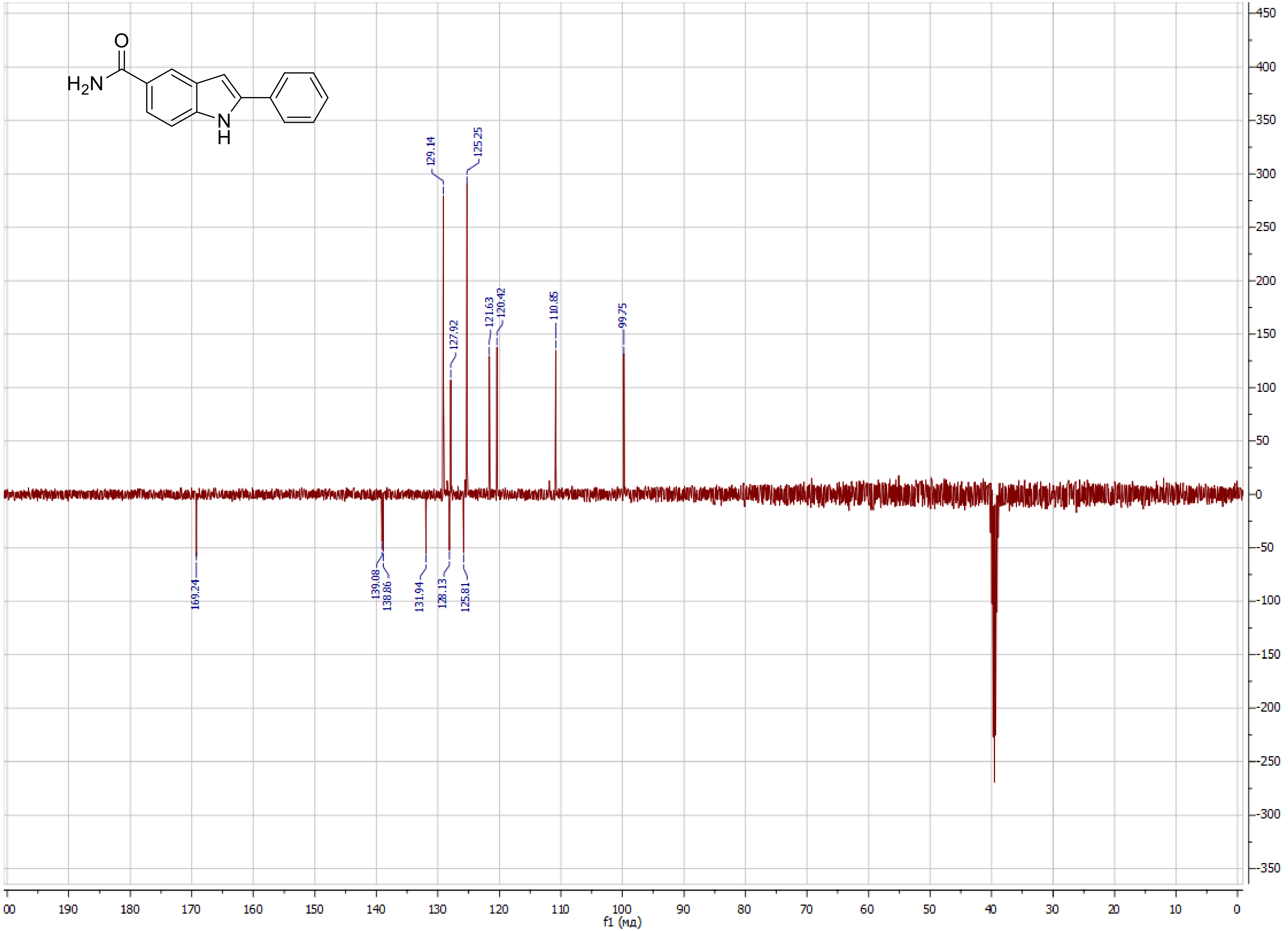

Figure S14. $^{13}C\{^{1}H\}$ NMR spectrum of indole **3ai** in DMSO-d_6 (100 MHz)

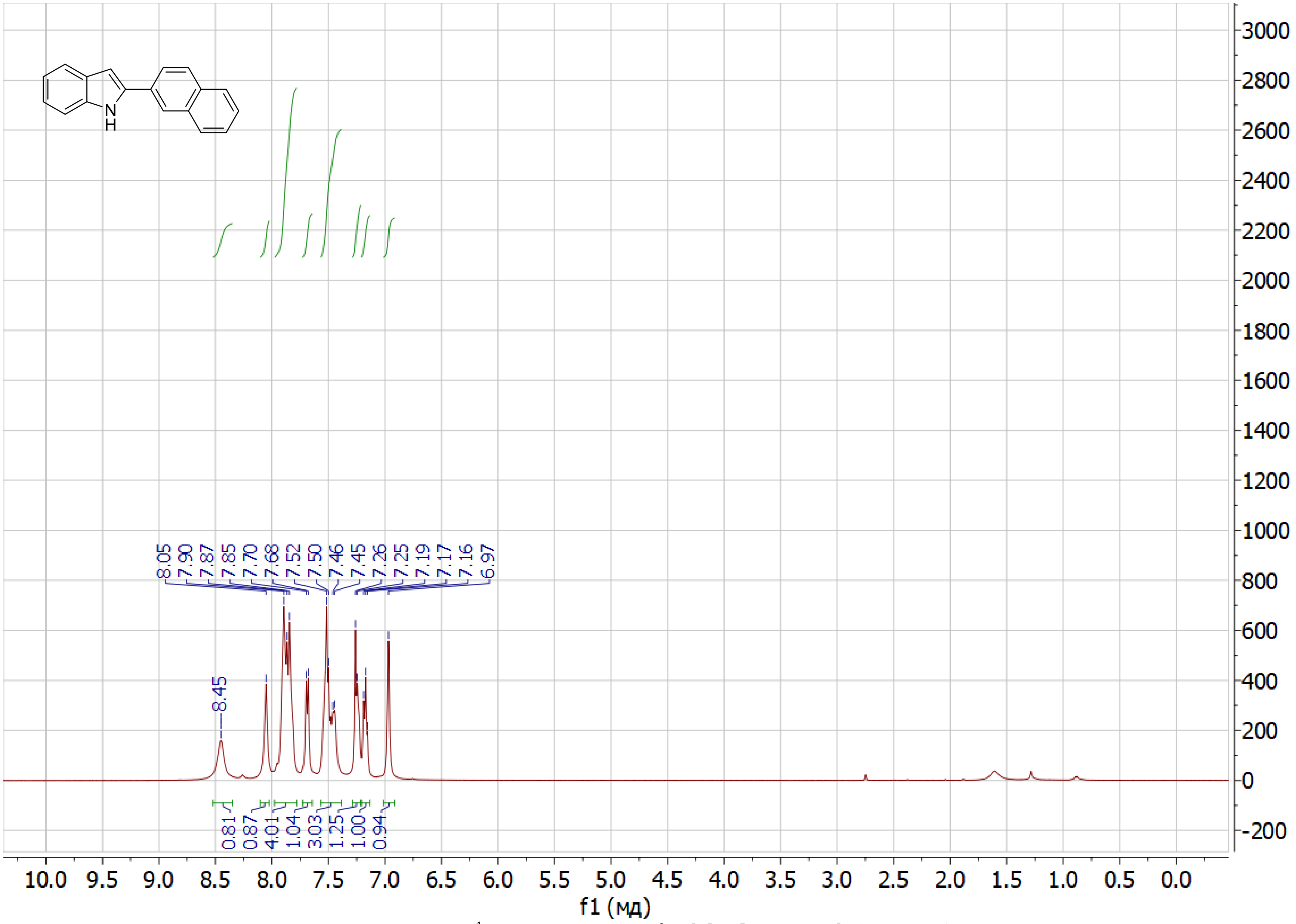

Figure S15. ^{1}H NMR spectrum of indole **3ba** in $CDCl_3$ (400 MHz)

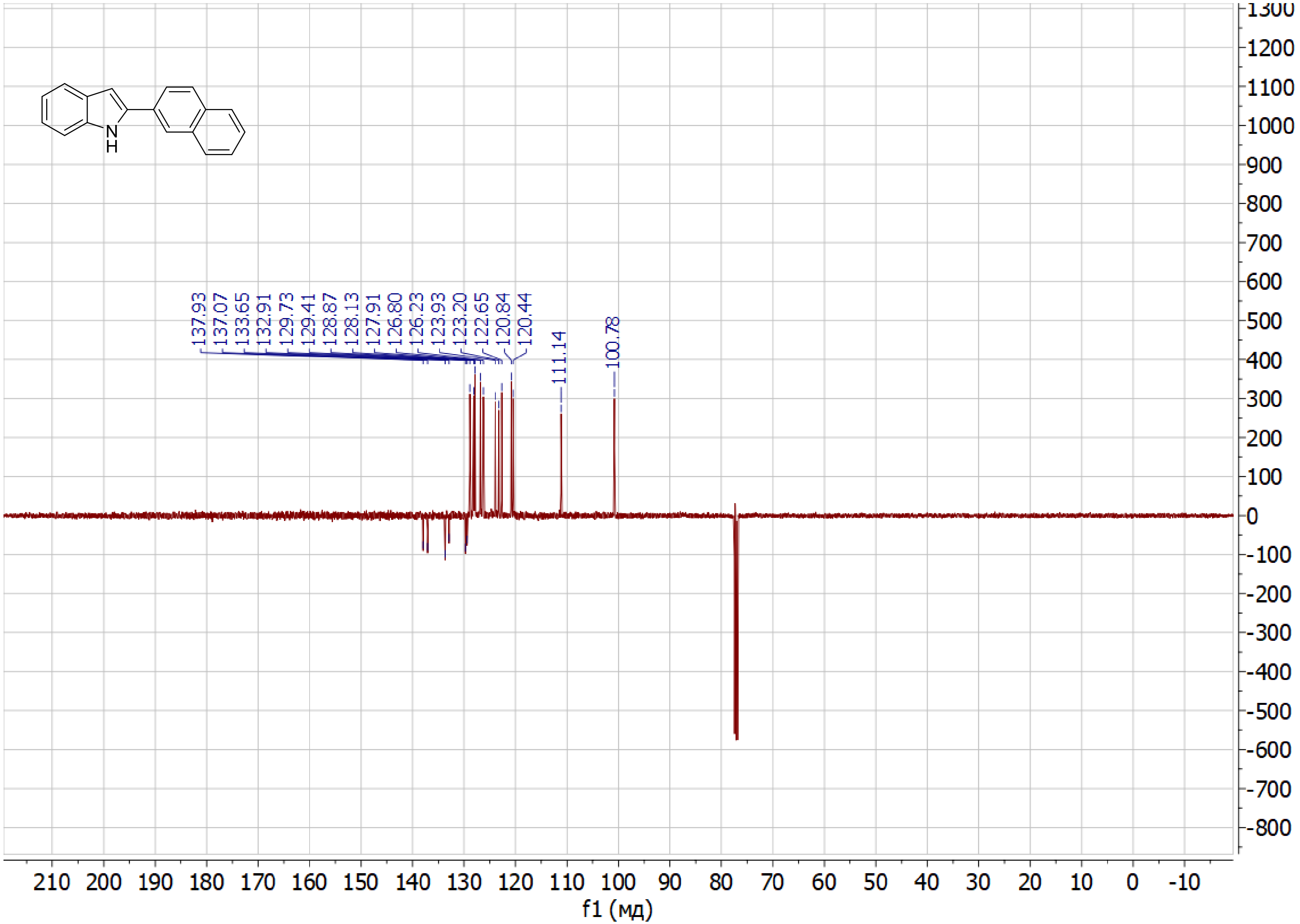

Figure S16. $^{13}C\{^{1}H\}$ NMR spectrum of indole **3ba** in $CDCl_3$ (100 MHz)

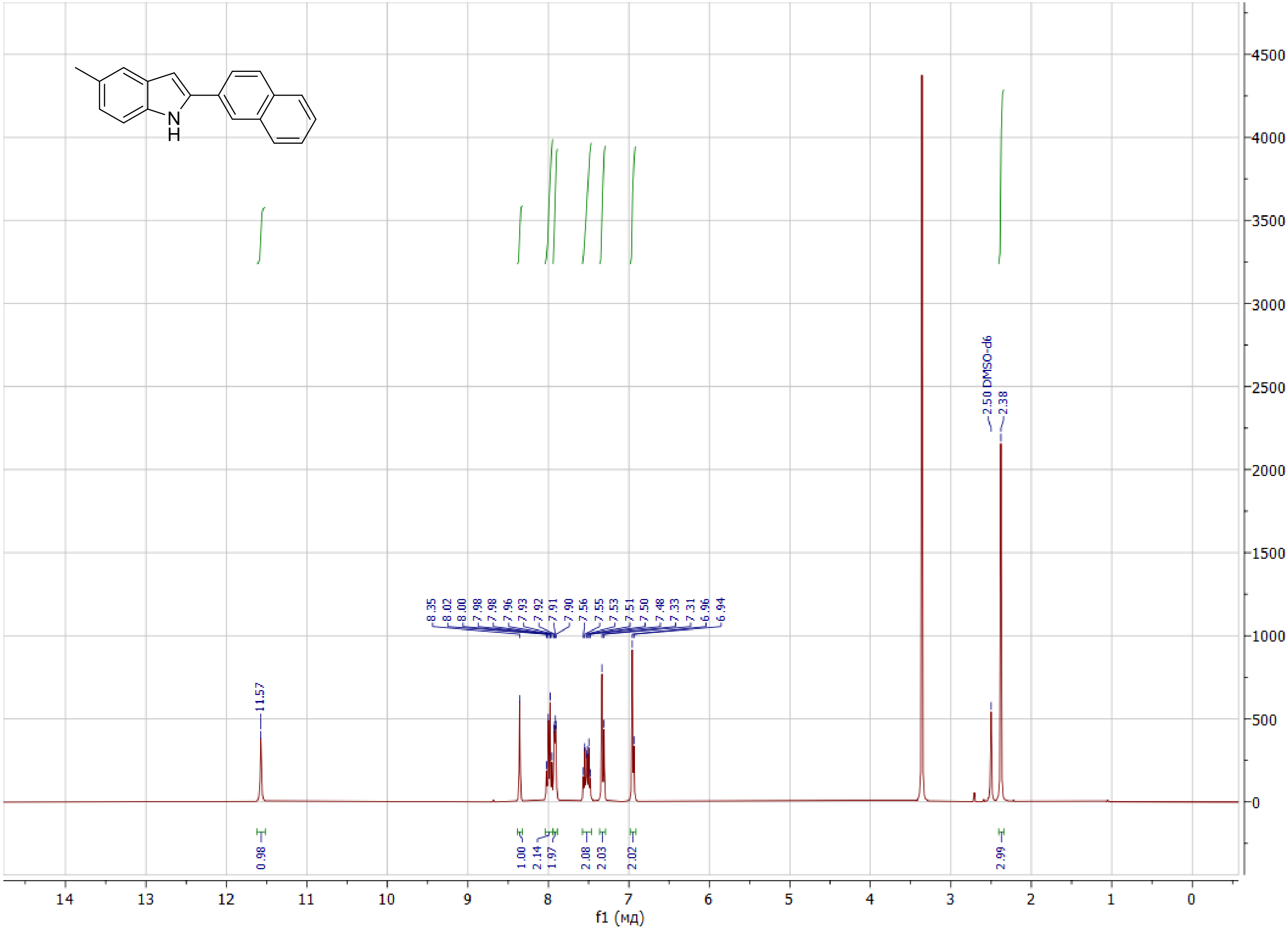

Figure S17. ^{1}H NMR spectrum of indole **3bc** in DMSO-d_6 (400 MHz)

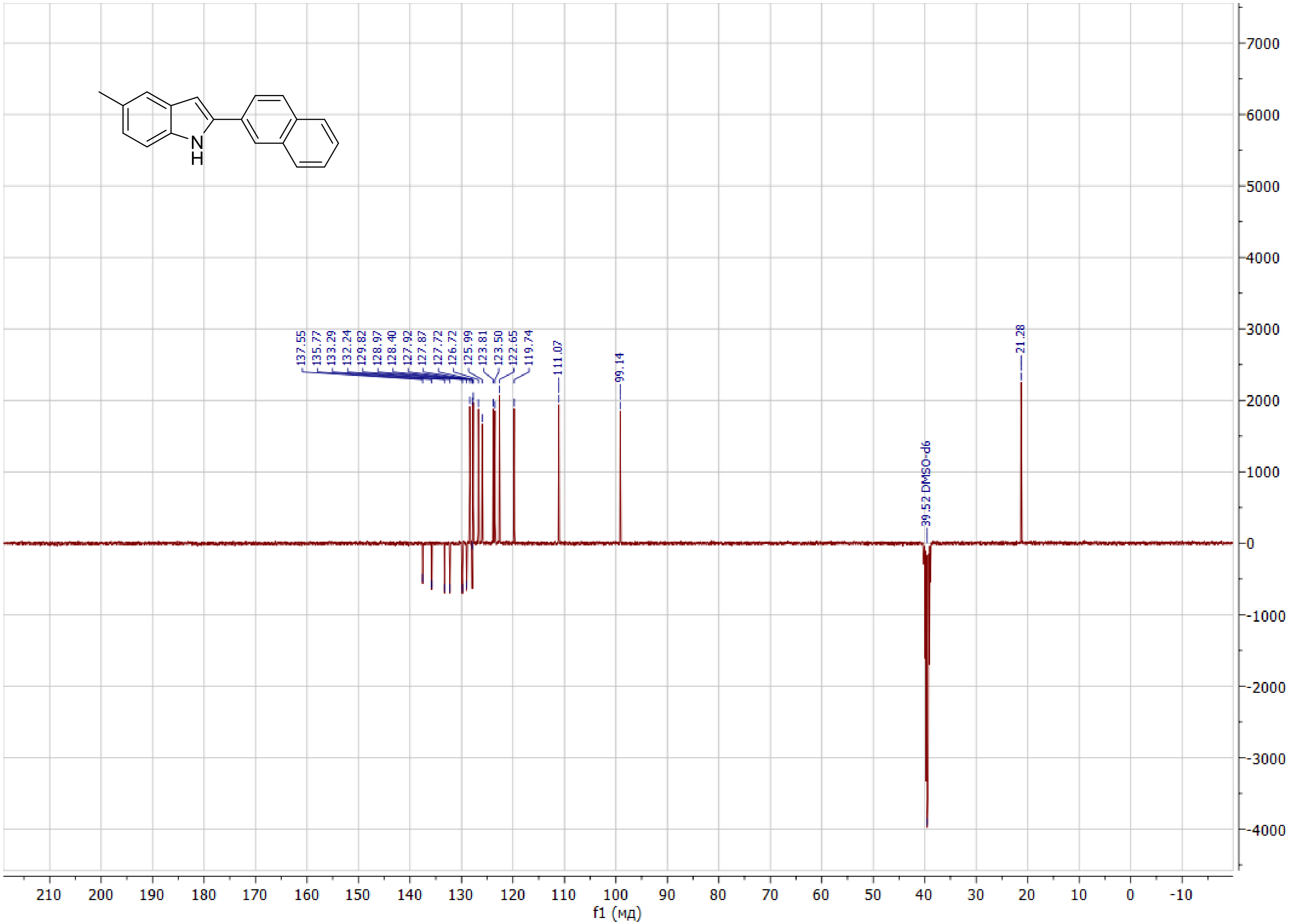

Figure S18. $^{13}C\{^{1}H\}$ NMR spectrum of indole **3bc** in DMSO-d_6 (100 MHz)

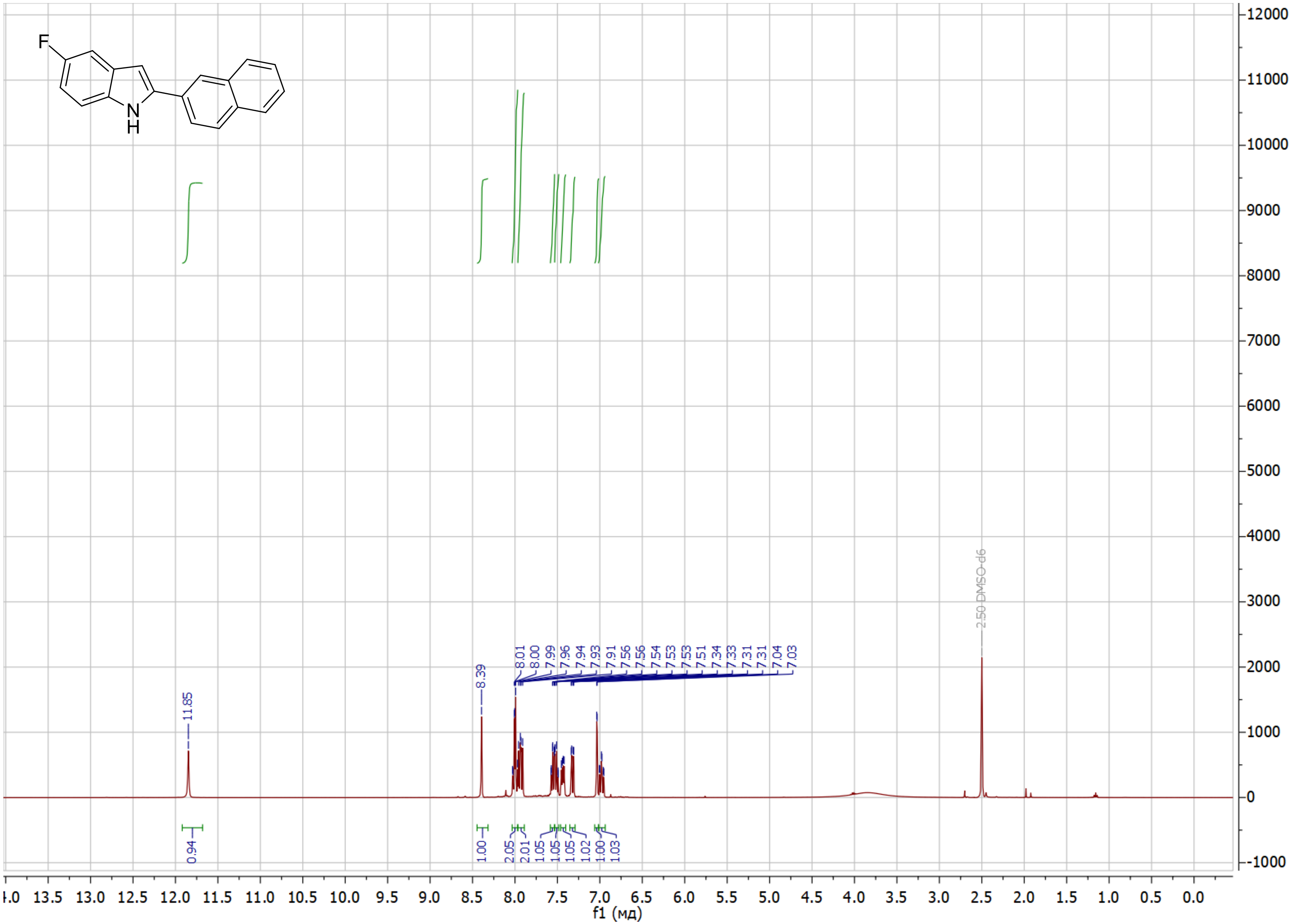

Figure S19. ^{1}H NMR spectrum of indole **3bf** in DMSO-d_6 (400 MHz)

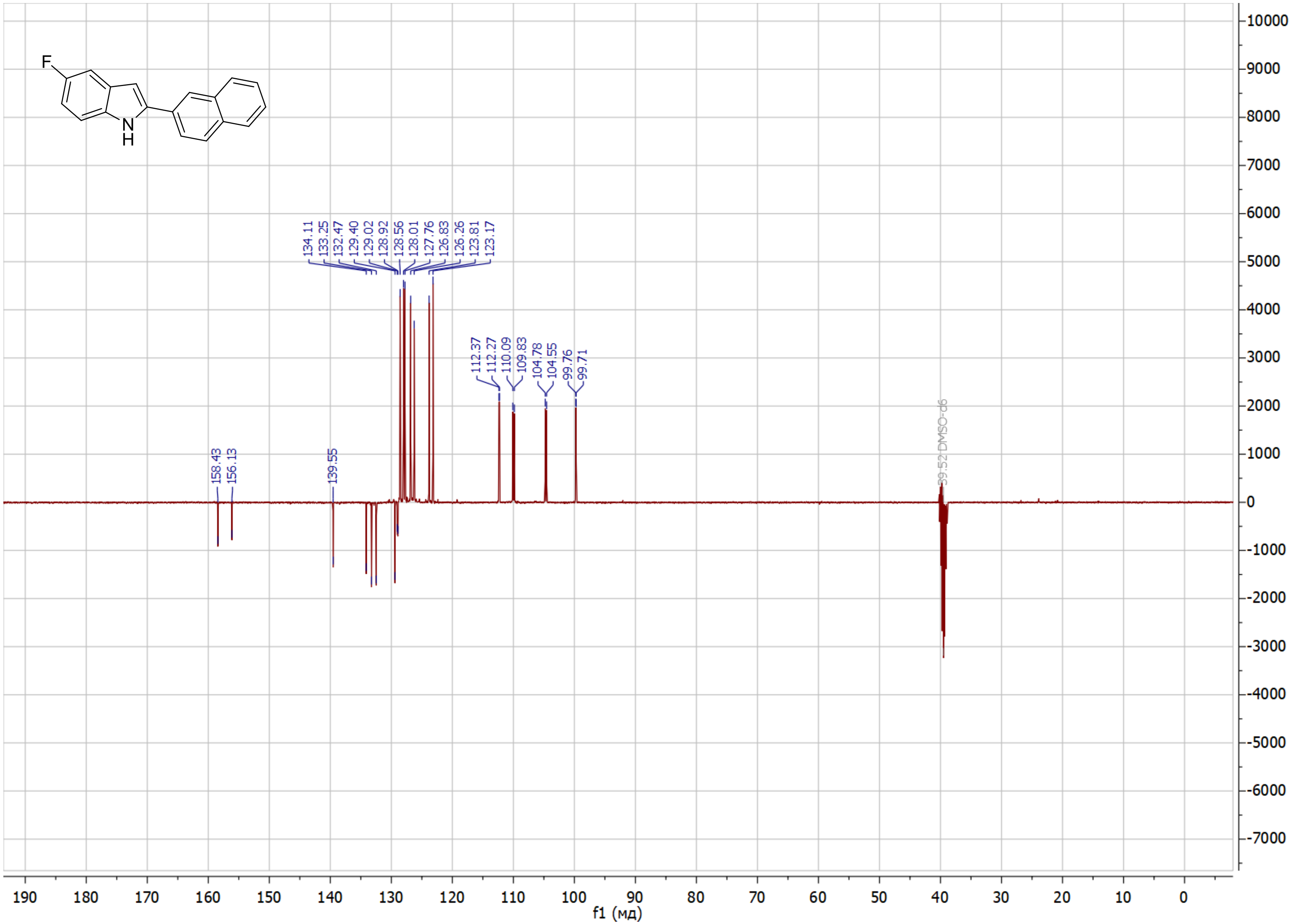

Figure S20. $^{13}C\{^{1}H\}$ NMR spectrum of indole **3bf** in DMSO-d_6 (100 MHz)

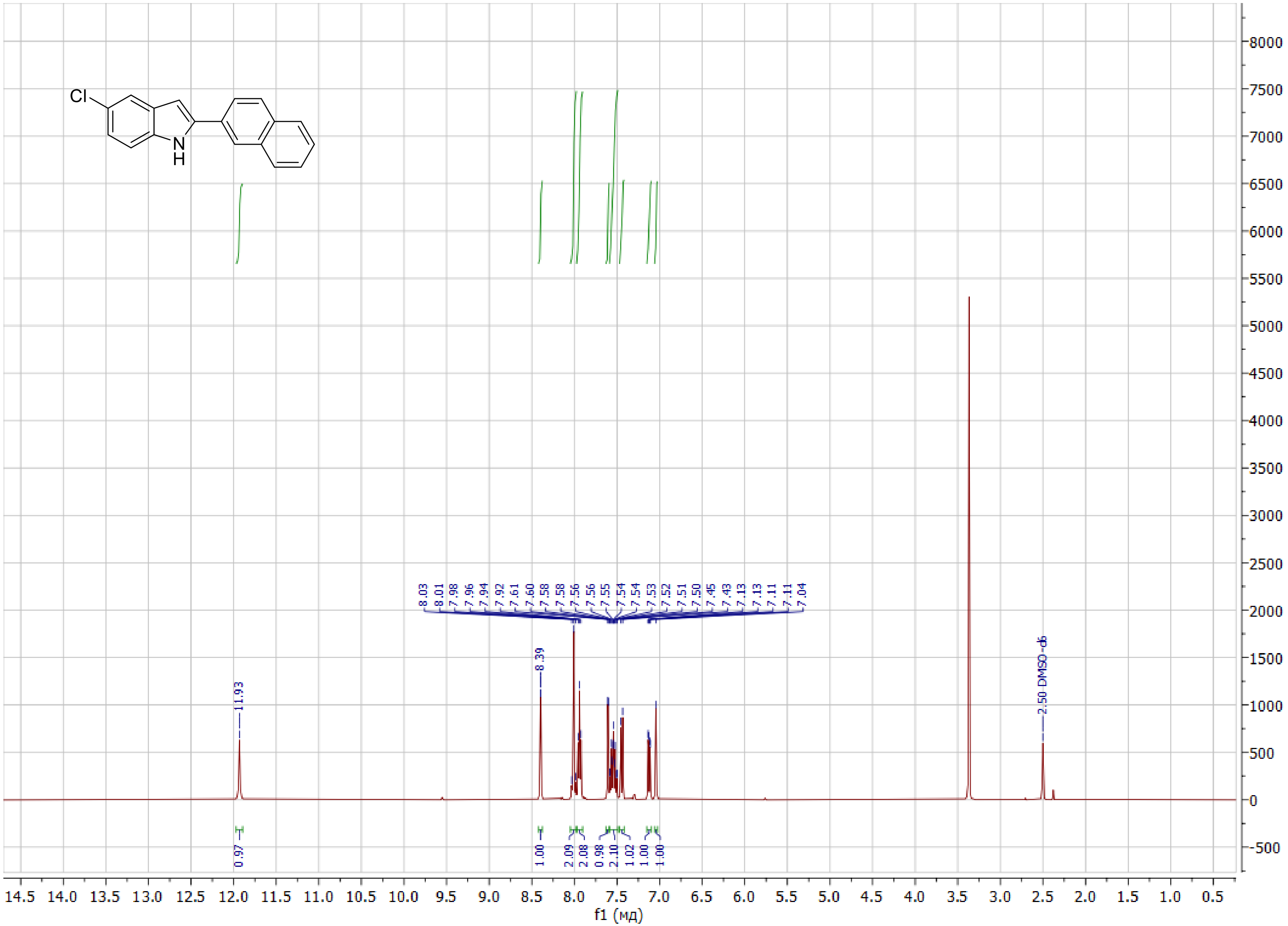

Figure S21. ^{1}H NMR spectrum of indole **3bg** in DMSO-d_6 (400 MHz)

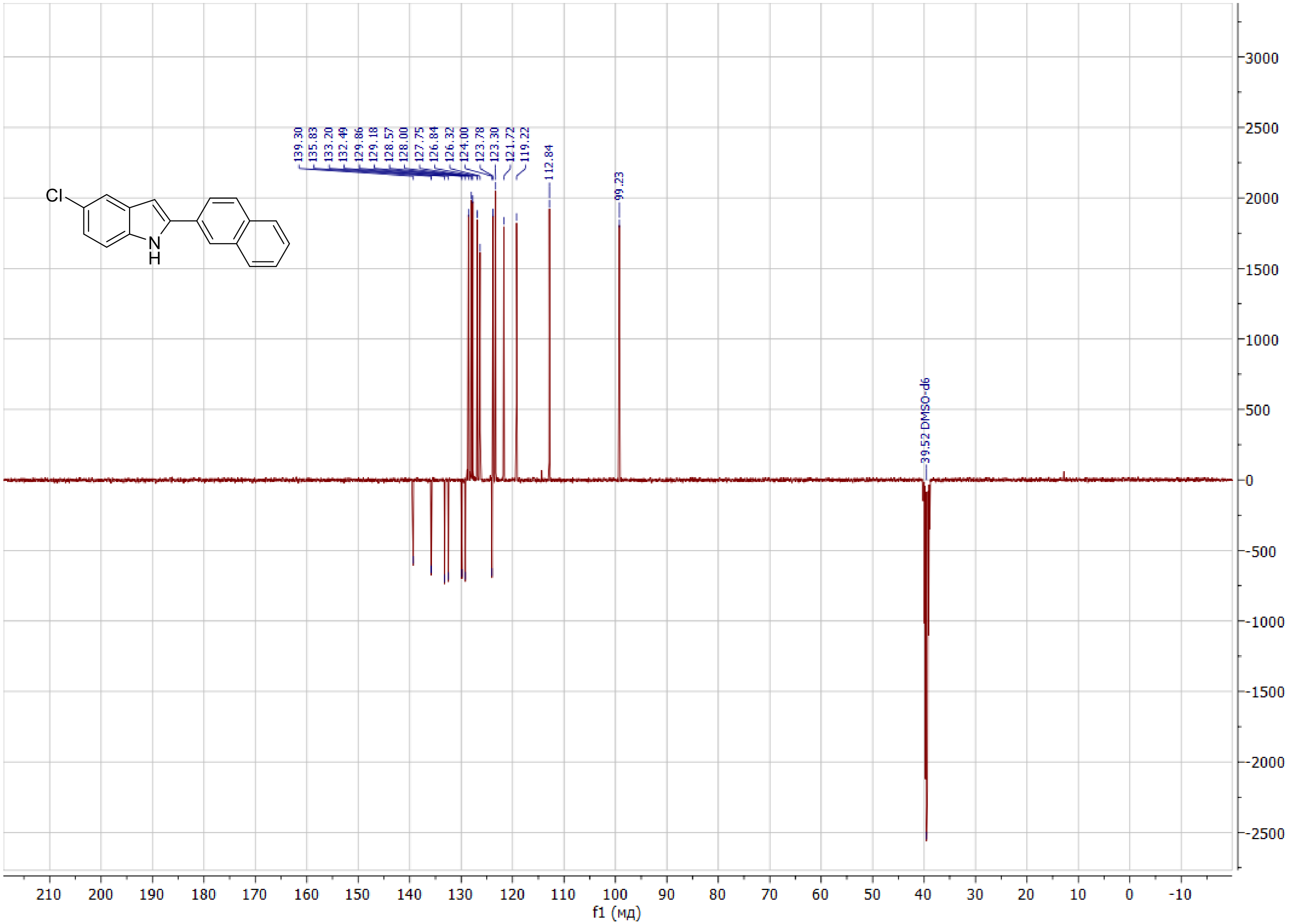

Figure S22. $^{13}C\{^{1}H\}$ NMR spectrum of indole **3bg** in DMSO-d_6 (100 MHz)

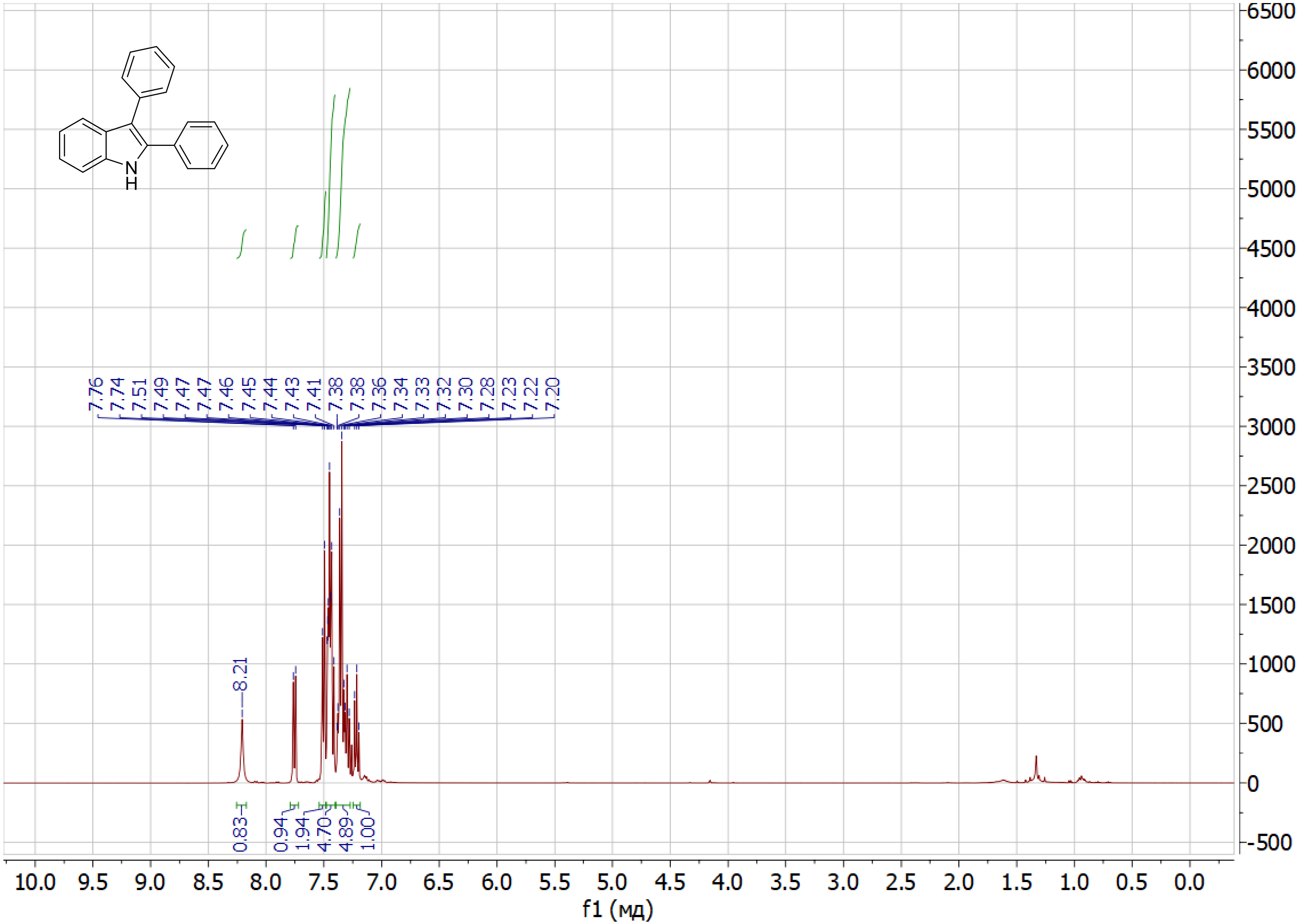

Figure S23. ^{1}H NMR spectrum of indole **3ca** in $CDCl_3$ (400 MHz)

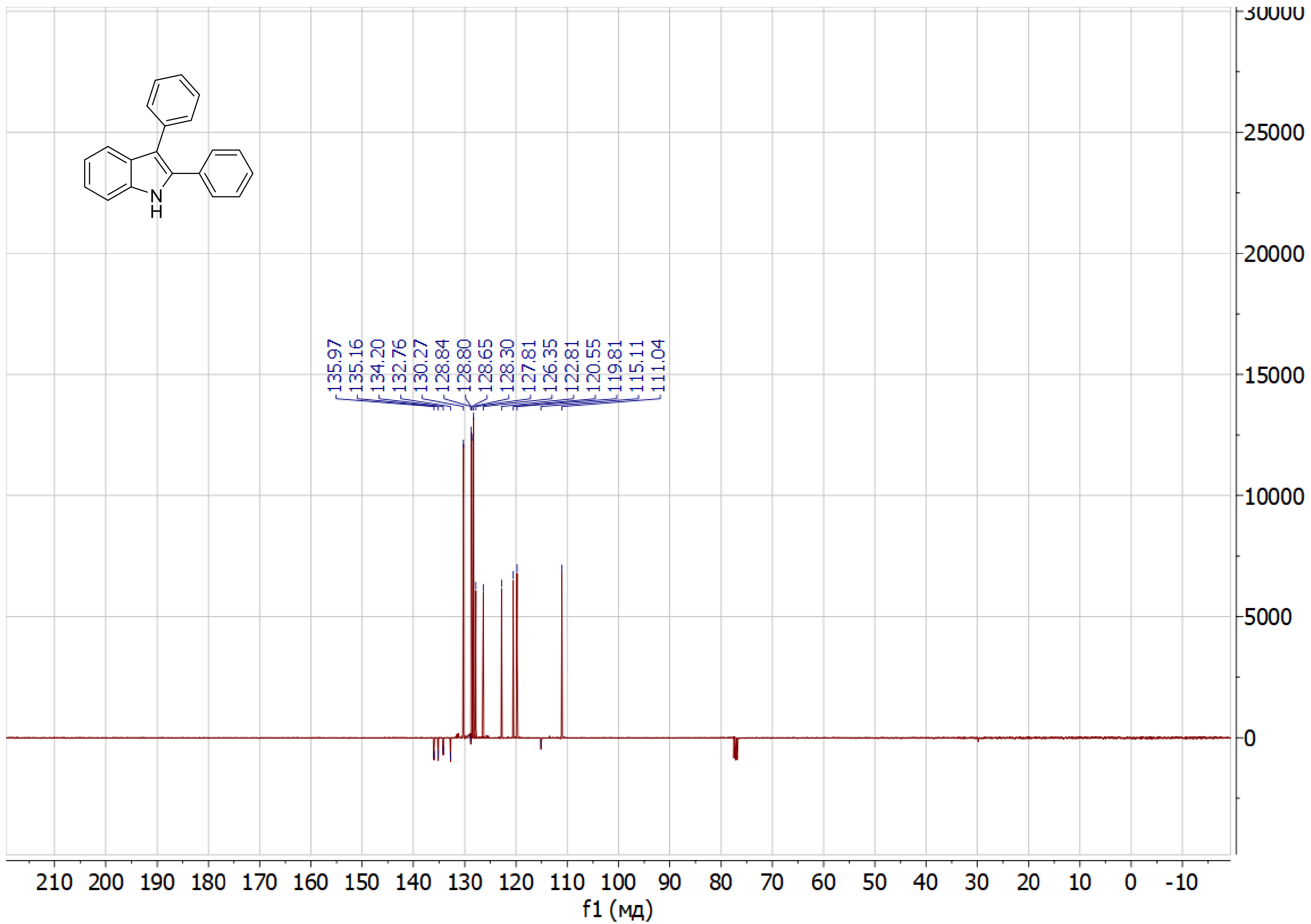

Figure S24. $^{13}C\{^{1}H\}$ NMR spectrum of indole **3ca** in $CDCl_3$ (100 MHz)

^{1}H and ^{13}C NMR spectral charts for acetophenones **7**

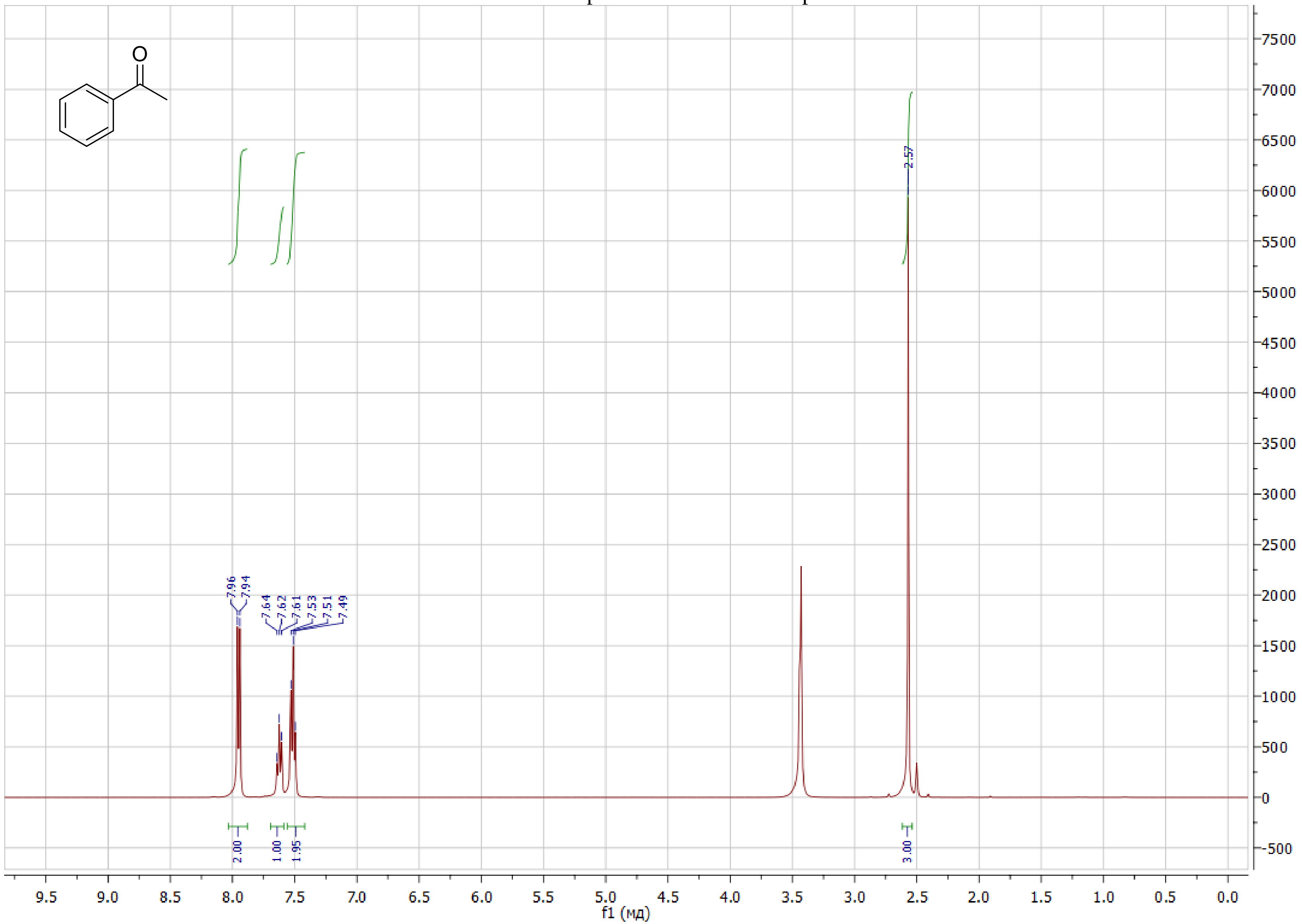

Figure S25. ^{1}H NMR spectrum of acetophenone **7a** in DMSO-d_6 (400 MHz)

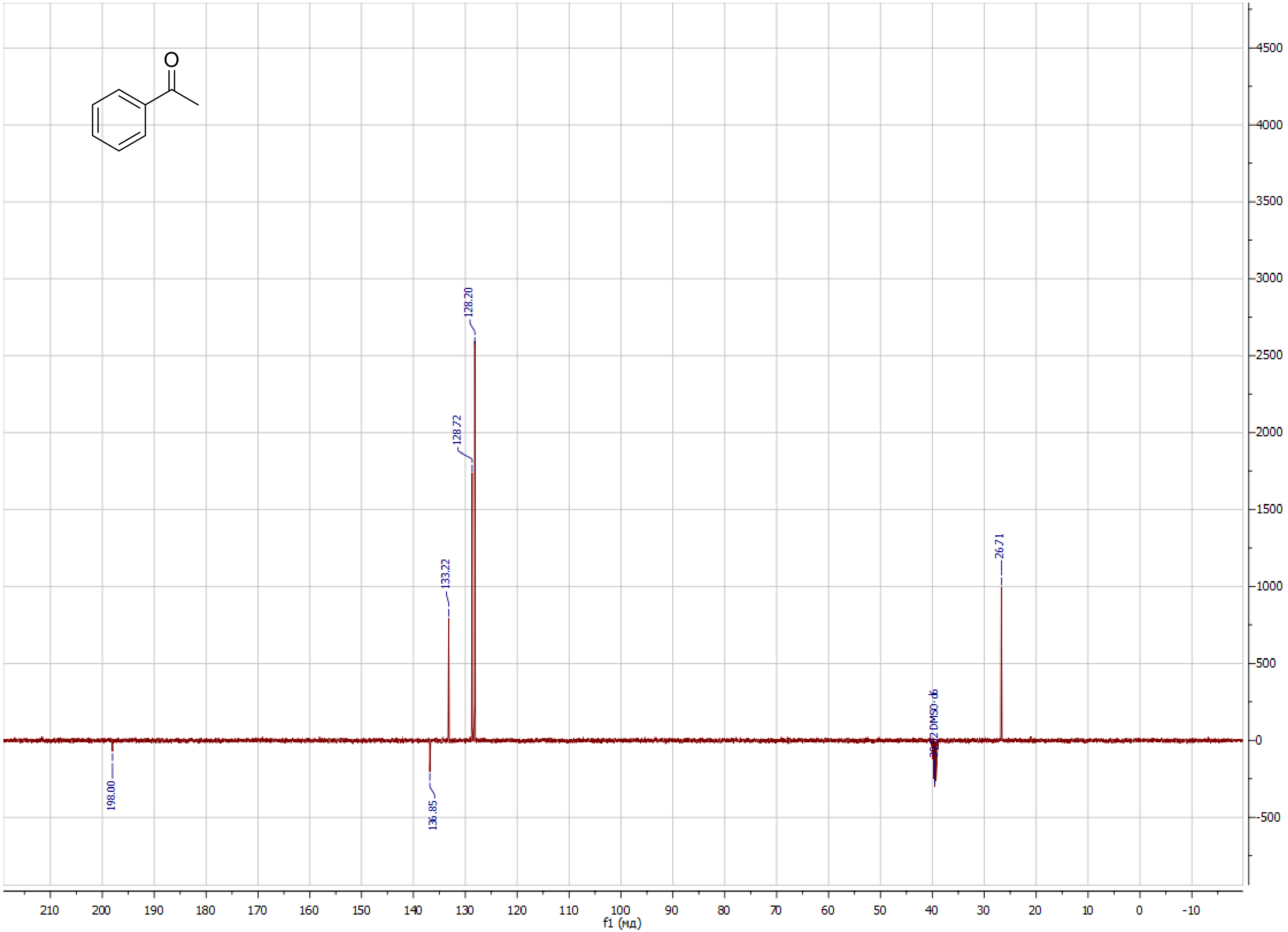

Figure S26. $^{13}C\{^{1}H\}$ NMR spectrum of acetophenone **7a** in DMSO-d_6 (100 MHz)

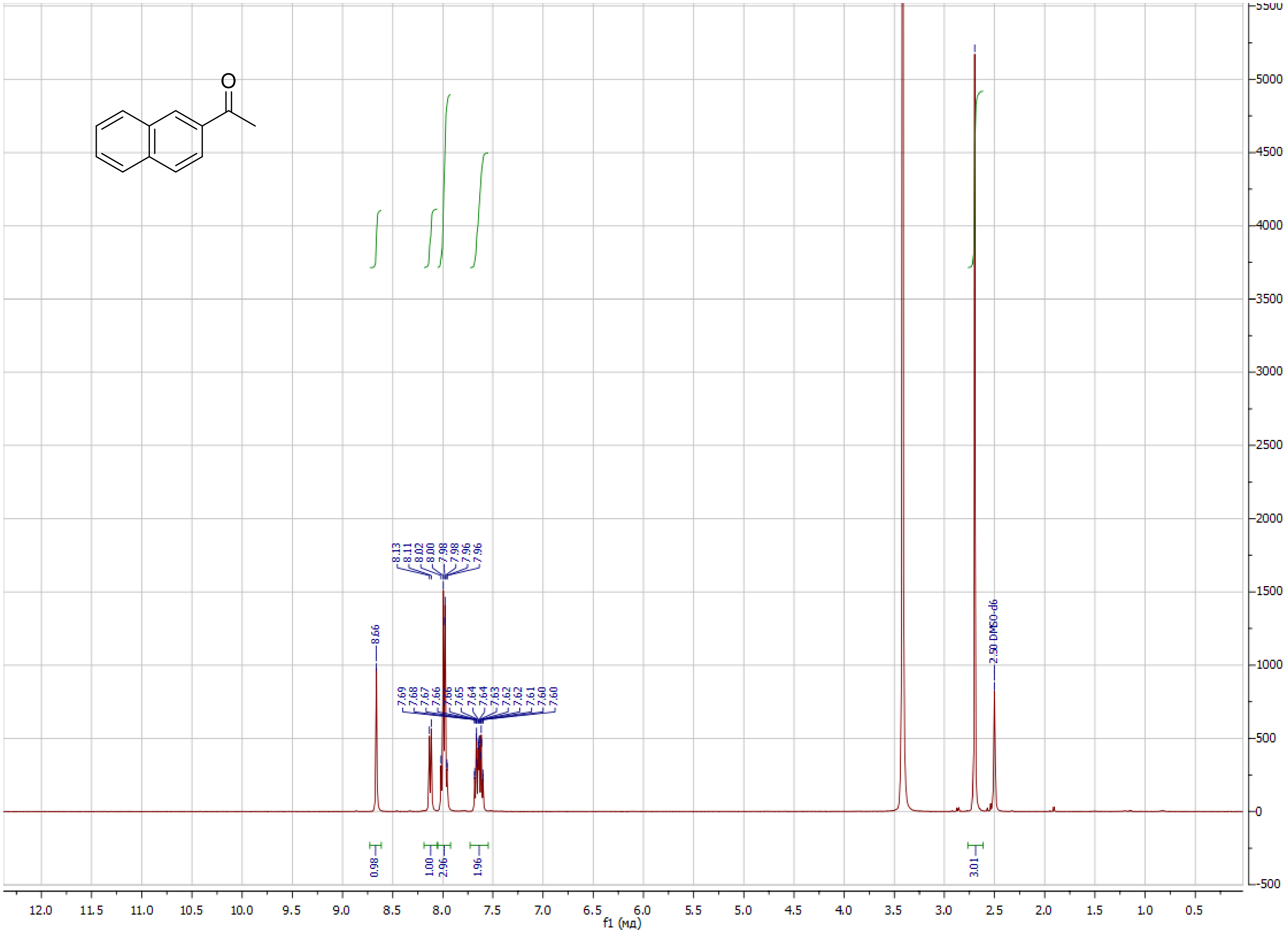

Figure S27. ^{1}H NMR spectrum of acetophenone **7b** in DMSO-d_6 (400 MHz)

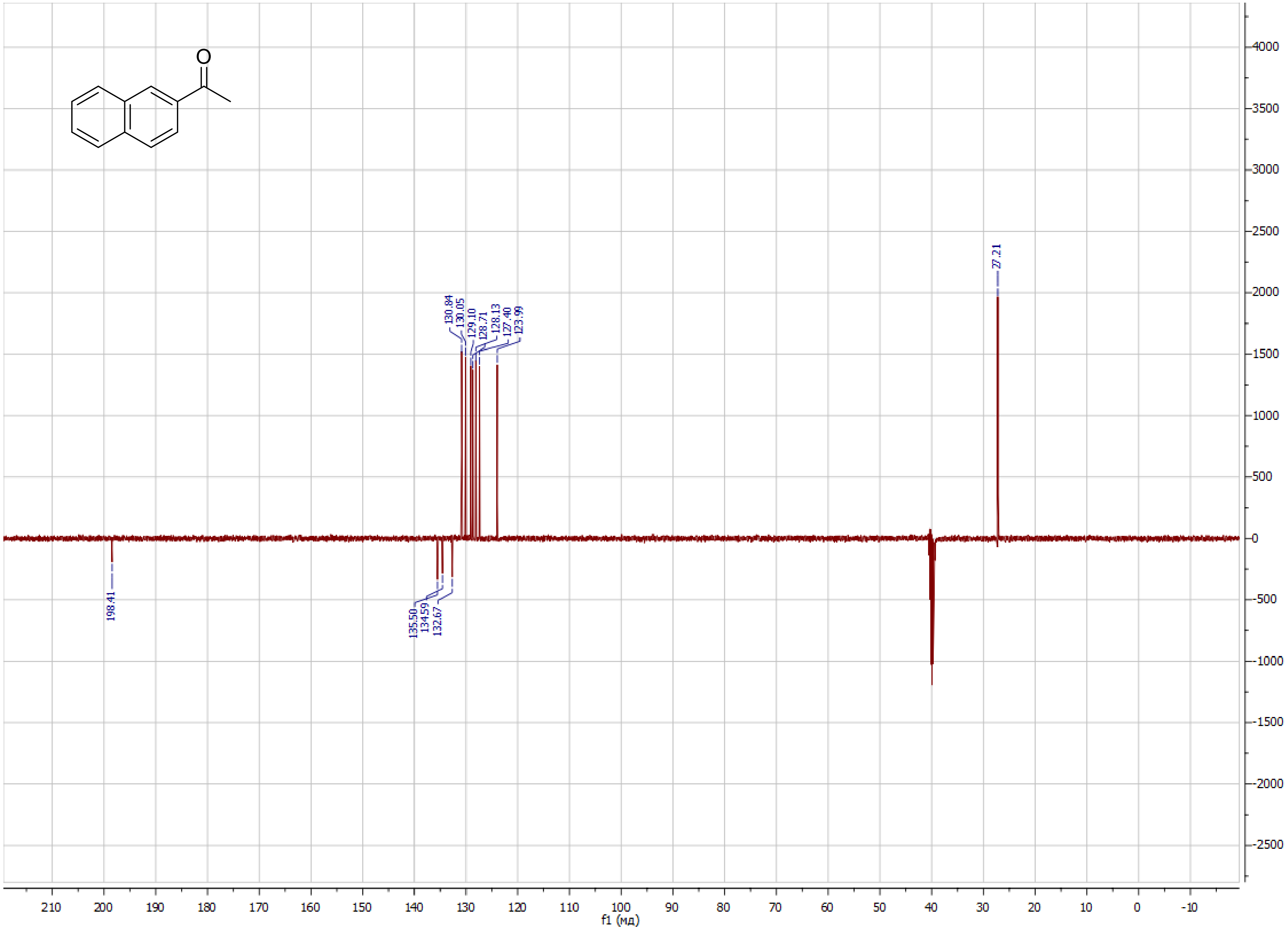

Figure S28. $^{13}C\{^{1}H\}$ NMR spectrum of acetophenone **7b** in DMSO-d_6 (100 MHz)

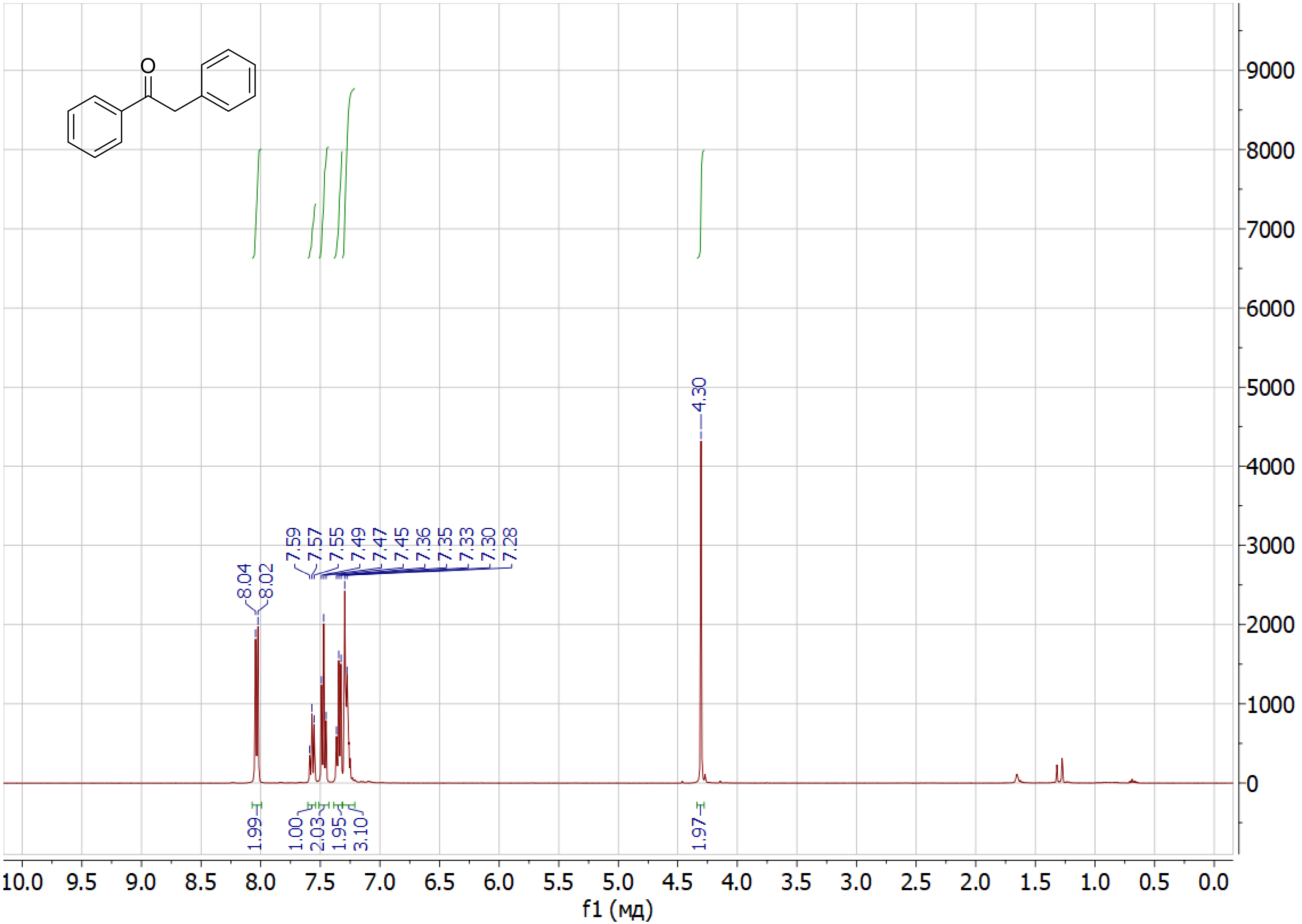

Figure S29. ^{1}H NMR spectrum of acetophenone **7c** in $CDCl_3$ (400 MHz)

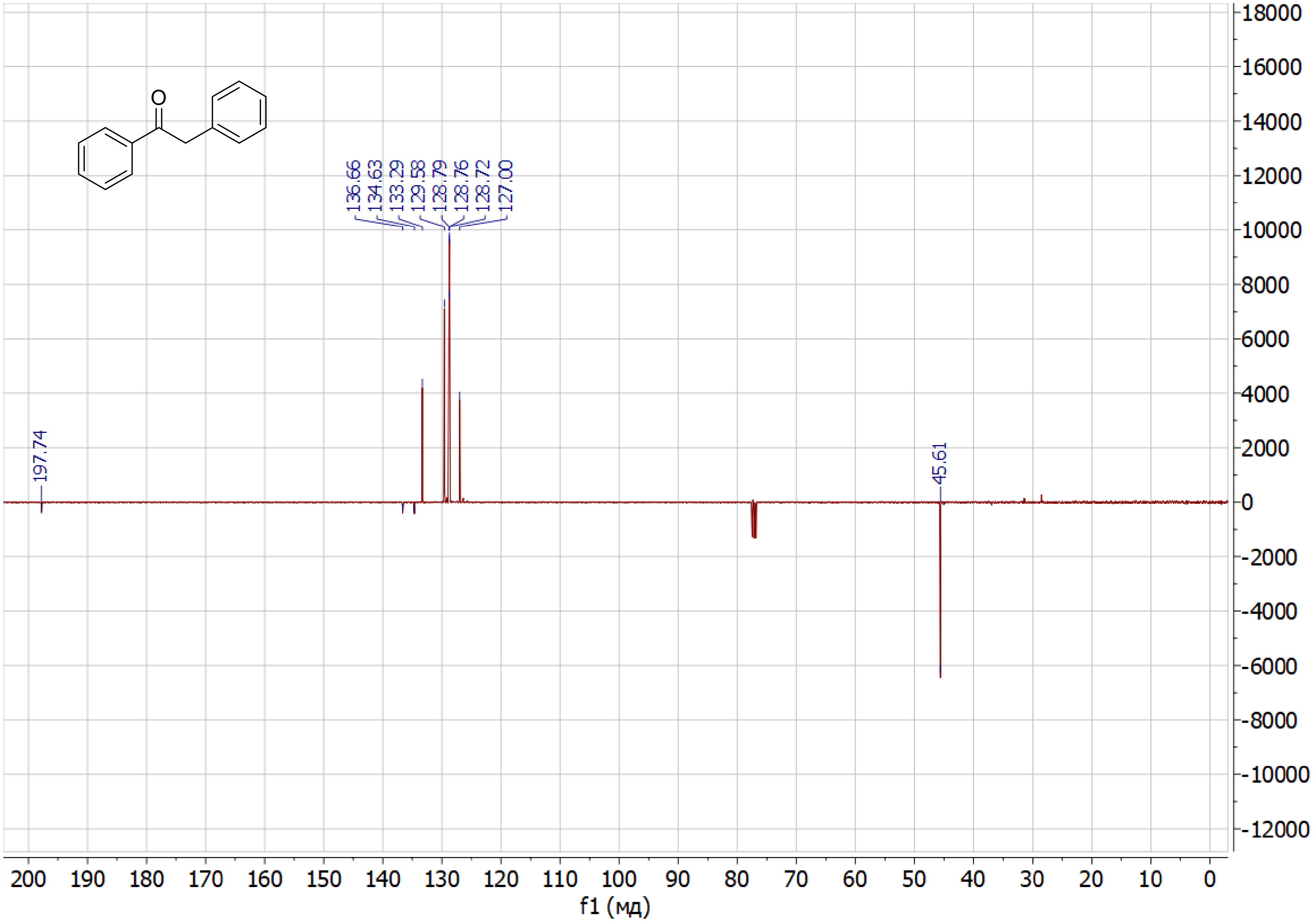

Figure S30. $^{13}C\{^{1}H\}$ NMR spectrum of acetophenone **7c** in $CDCl_3$ (100 MHz)

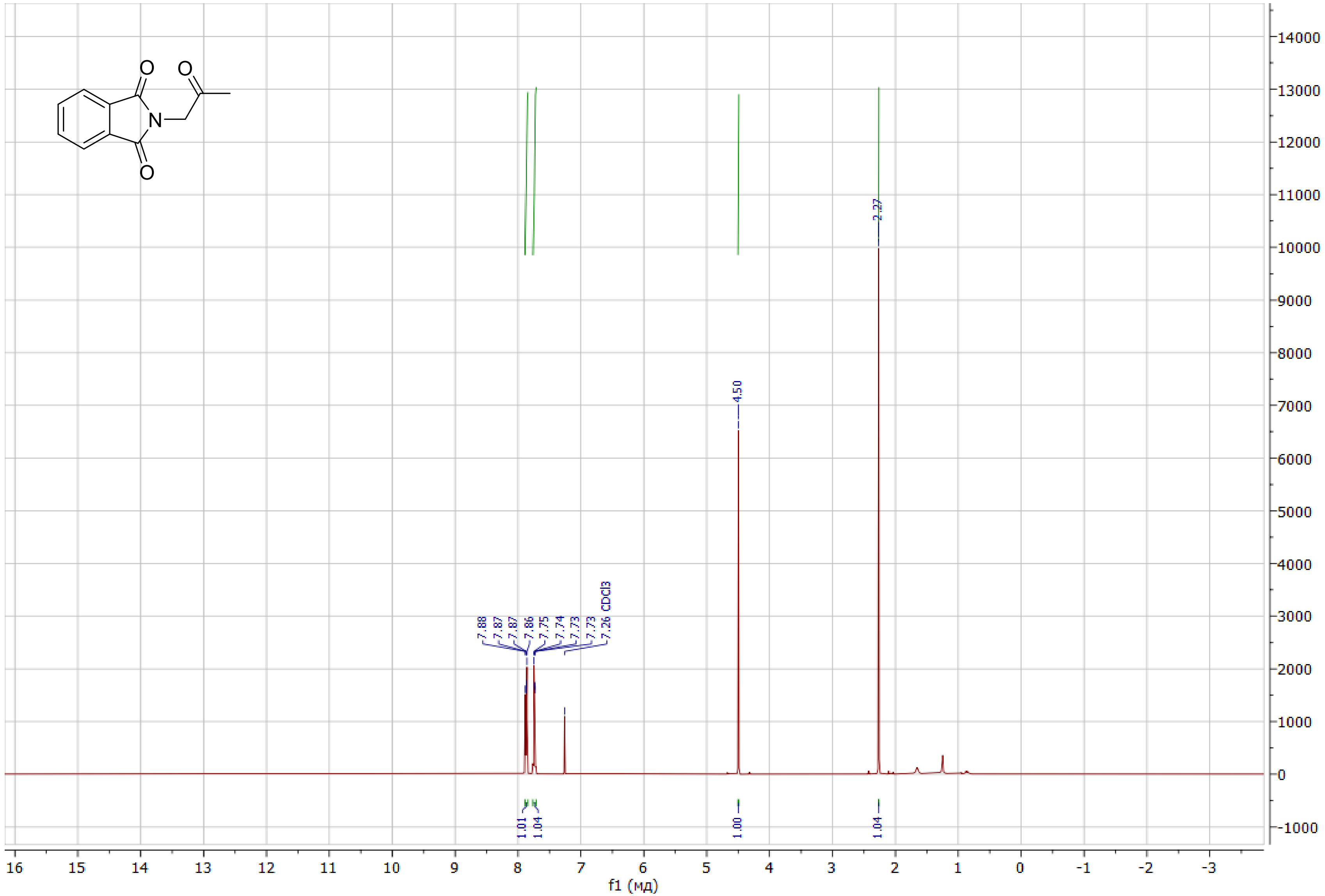

Figure S31. ^{1}H NMR spectrum of acetophenone **7d** in $CDCl_3$ (400 MHz)

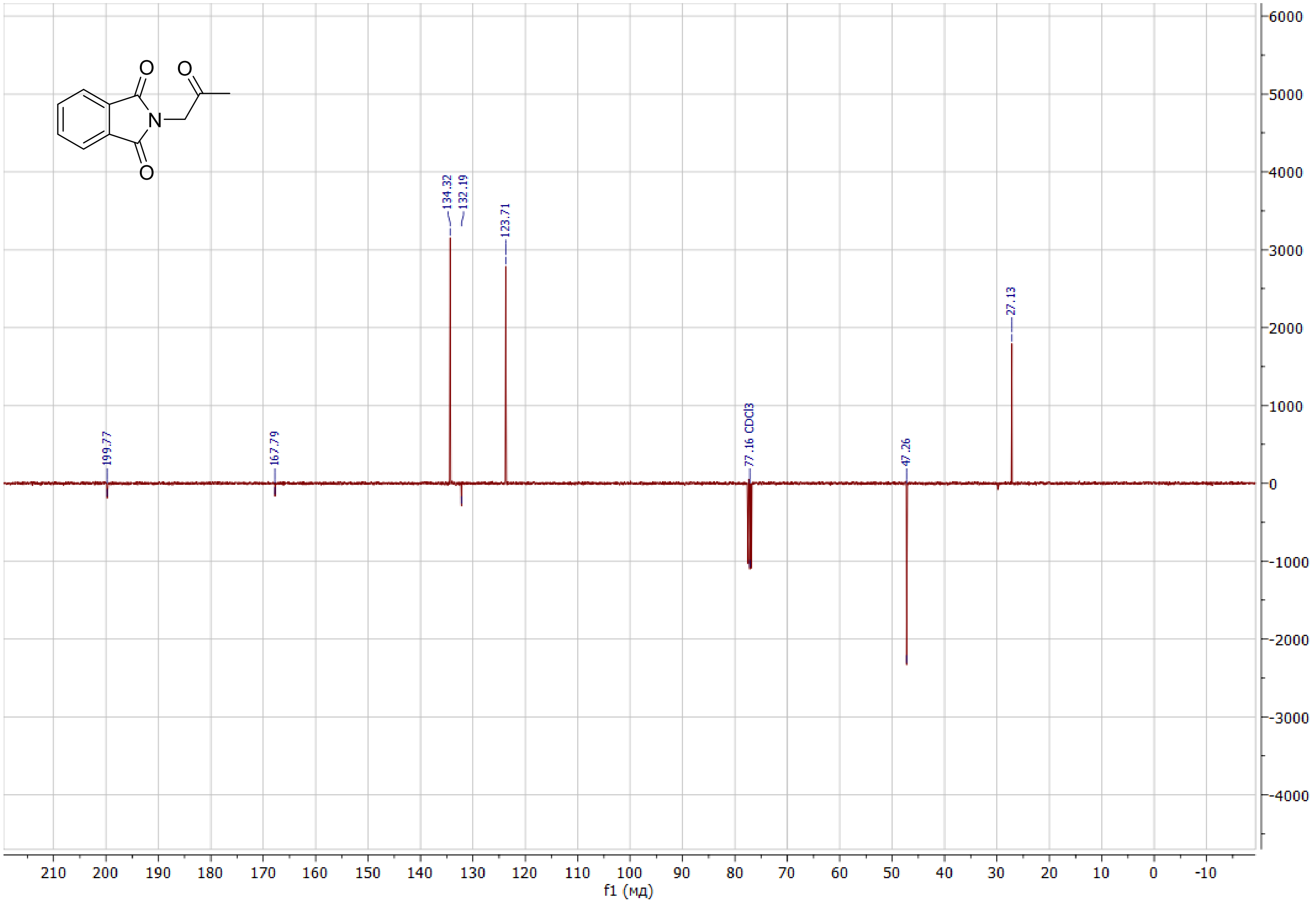

Figure S32. $^{13}C\{^{1}H\}$ NMR spectrum of acetophenone **7d** in $CDCl_3$ (100 MHz)

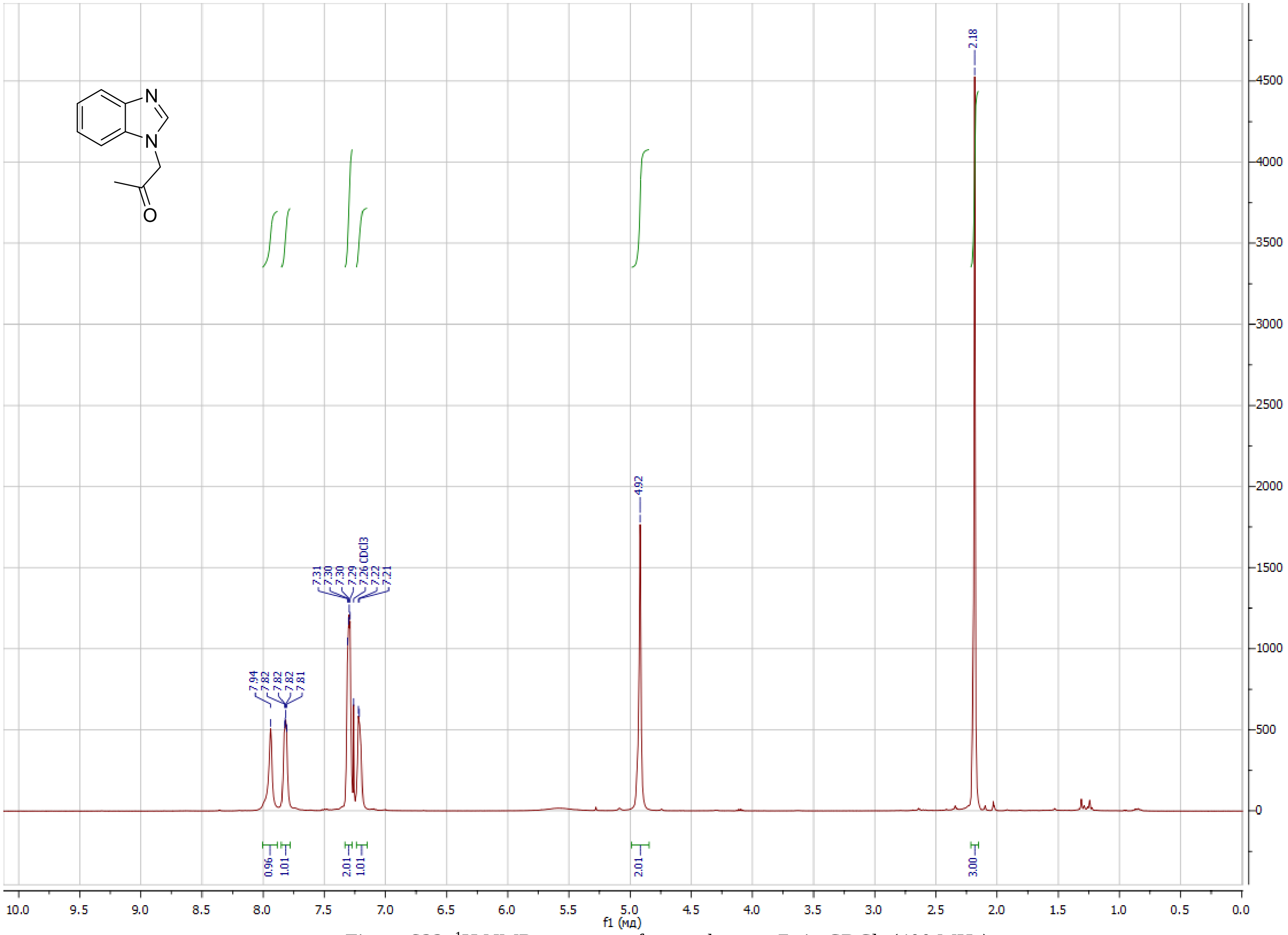

Figure S33. ^{1}H NMR spectrum of acetophenone **7e** in $CDCl_3$ (400 MHz)

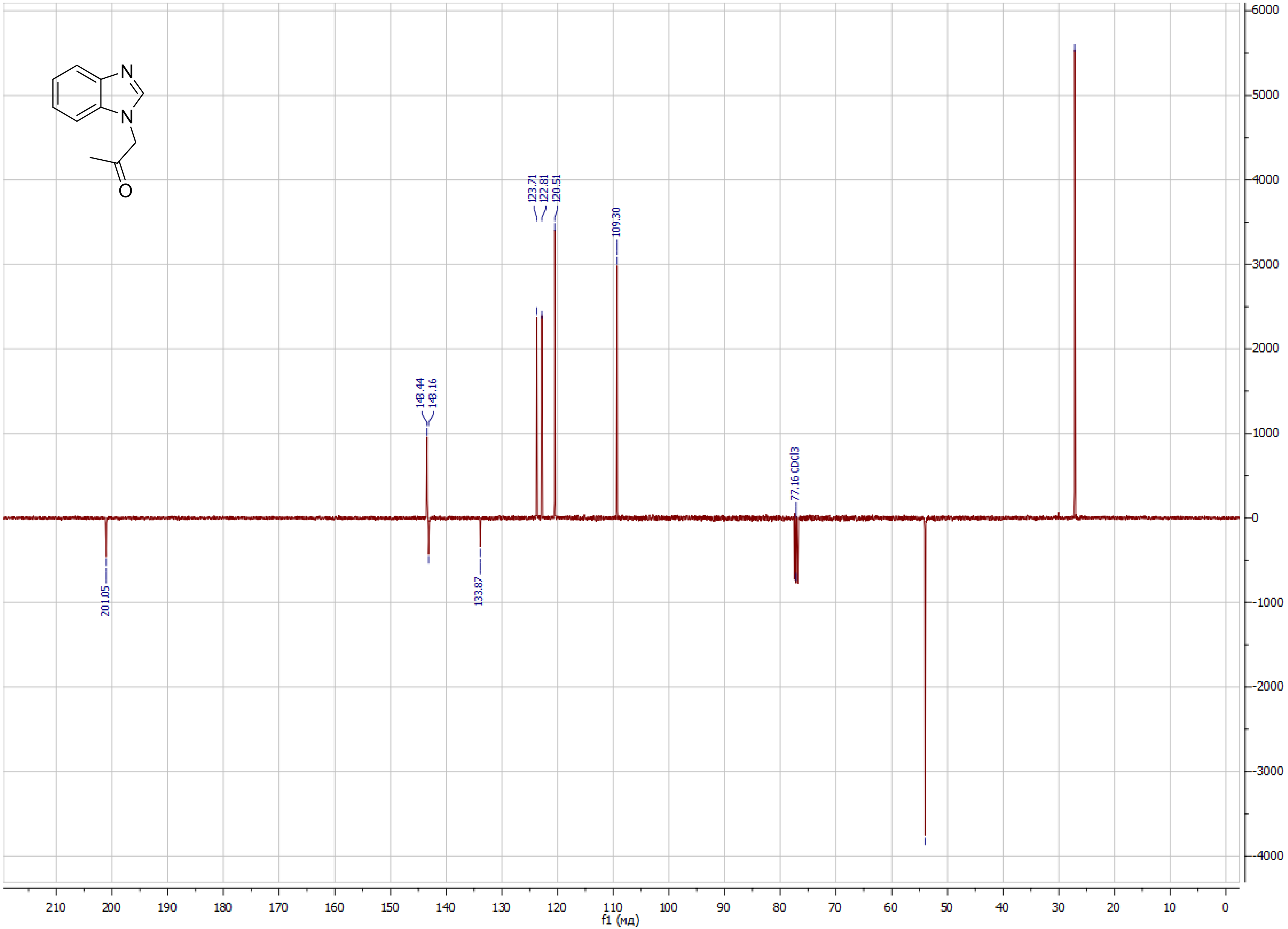

Figure S34. $^{13}C\{^{1}H\}$ NMR spectrum of acetophenone **7e** in $CDCl_3$ (100 MHz)

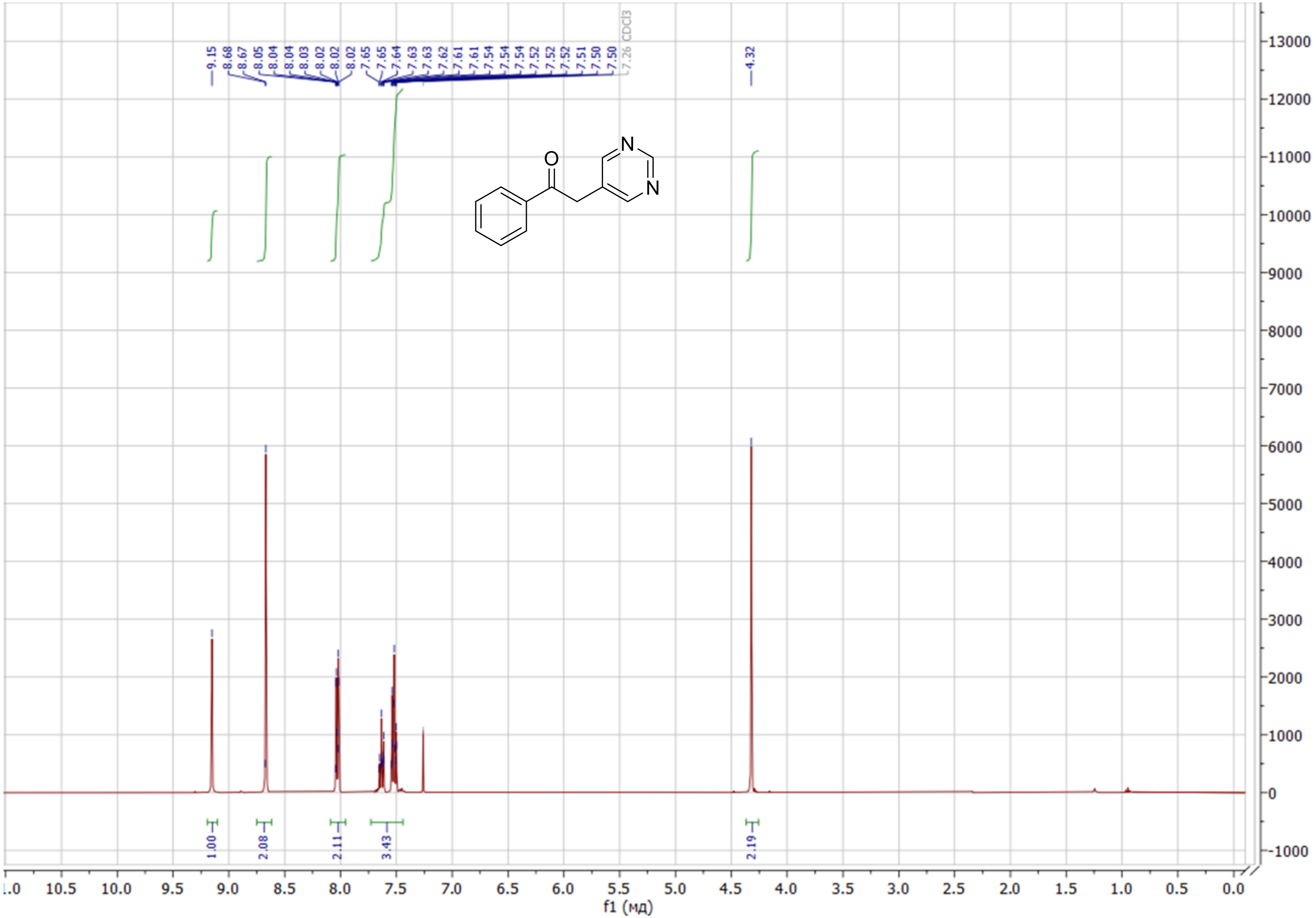

Figure S35. ^{1}H NMR spectrum of acetophenone **7f** in $CDCl_3$ (400 MHz)

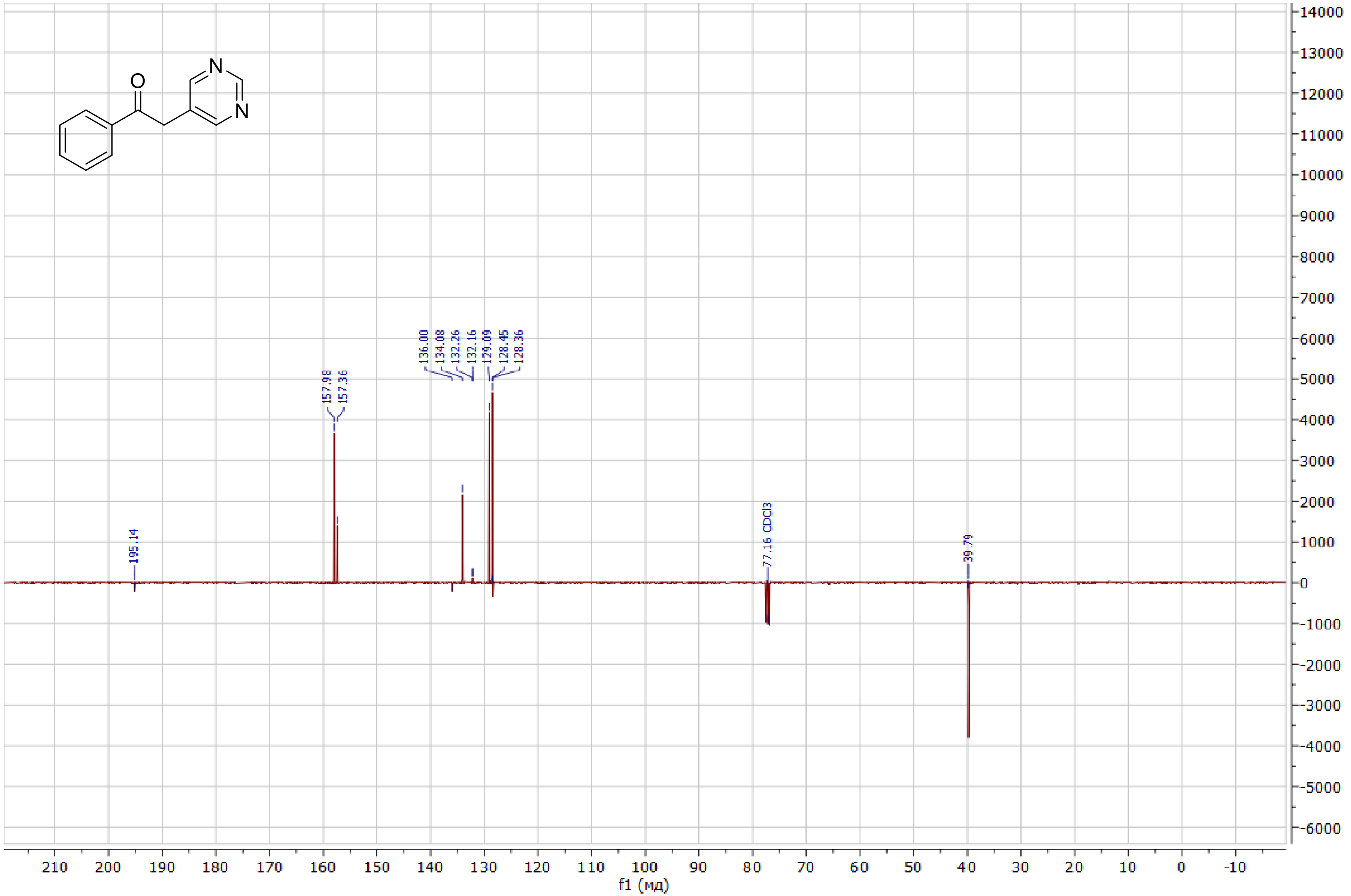

Figure S36. $^{13}C\{^{1}H\}$ NMR spectrum of acetophenone **7f** in $CDCl_3$ (100 MHz)

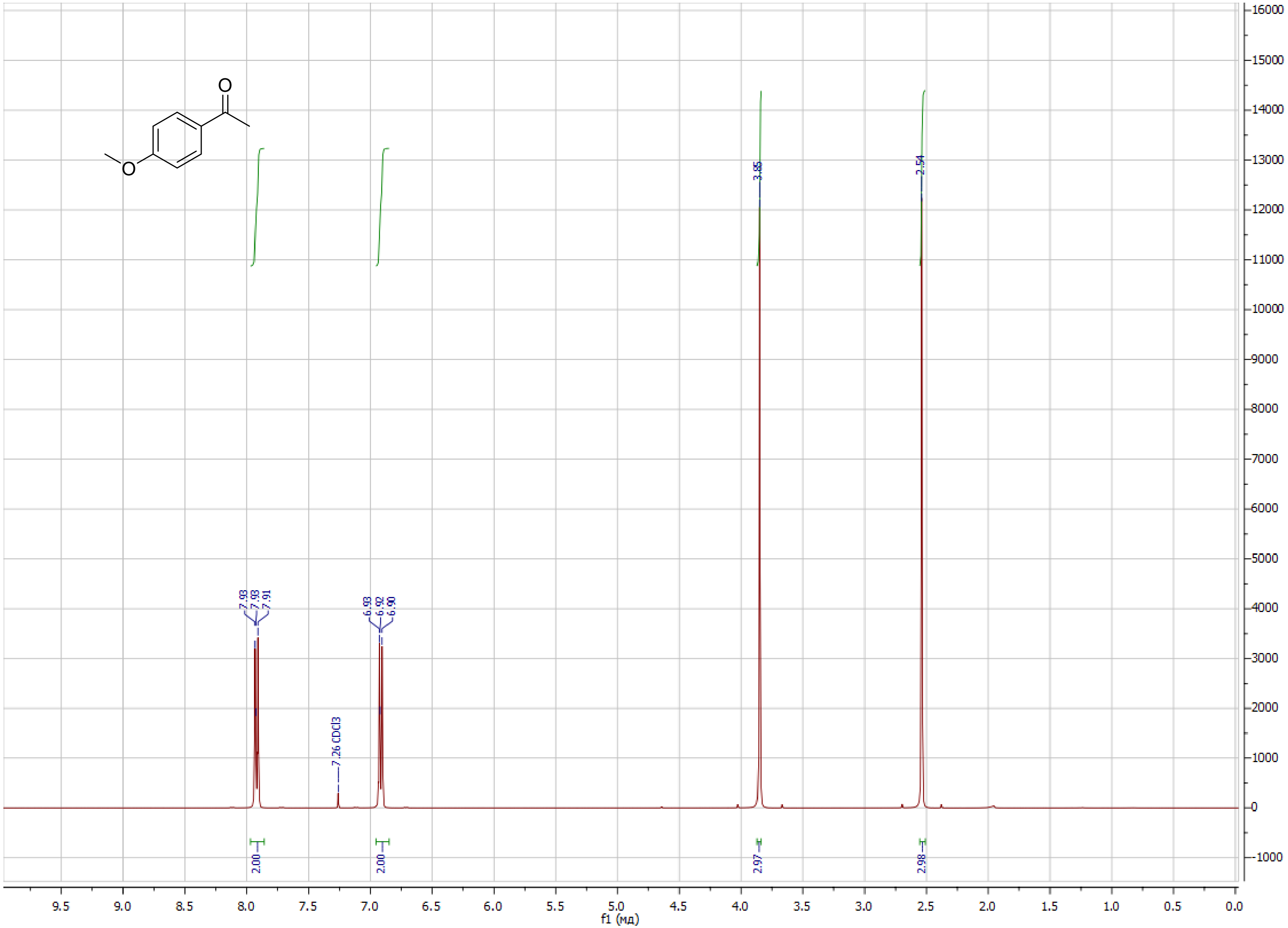

Figure S37. ^{1}H NMR spectrum of acetophenone **7h** in $CDCl_3$ (400 MHz)

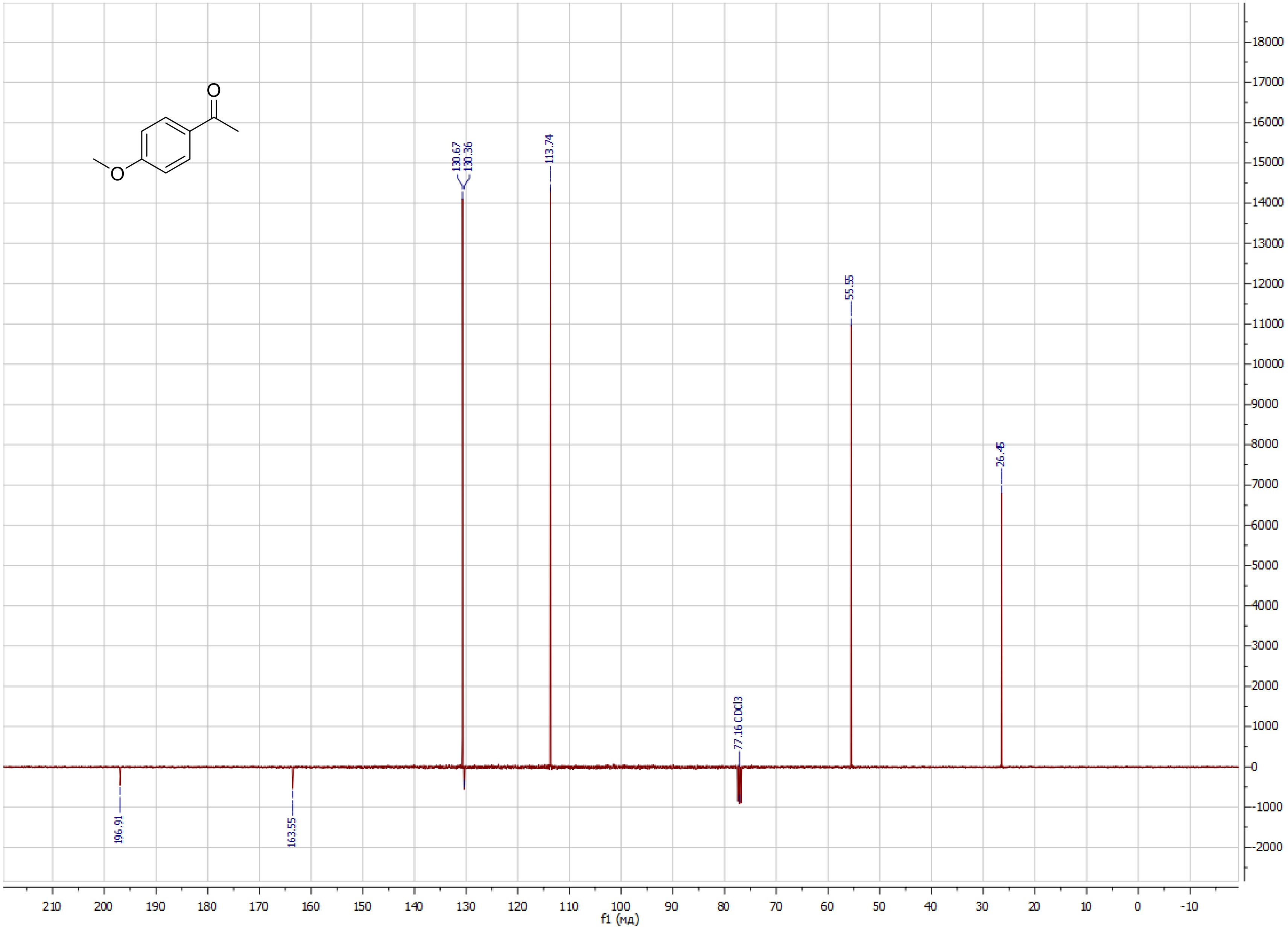

Figure S38. $^{13}C\{^{1}H\}$ NMR spectrum of acetophenone **7h** in $CDCl_3$ (100 MHz)

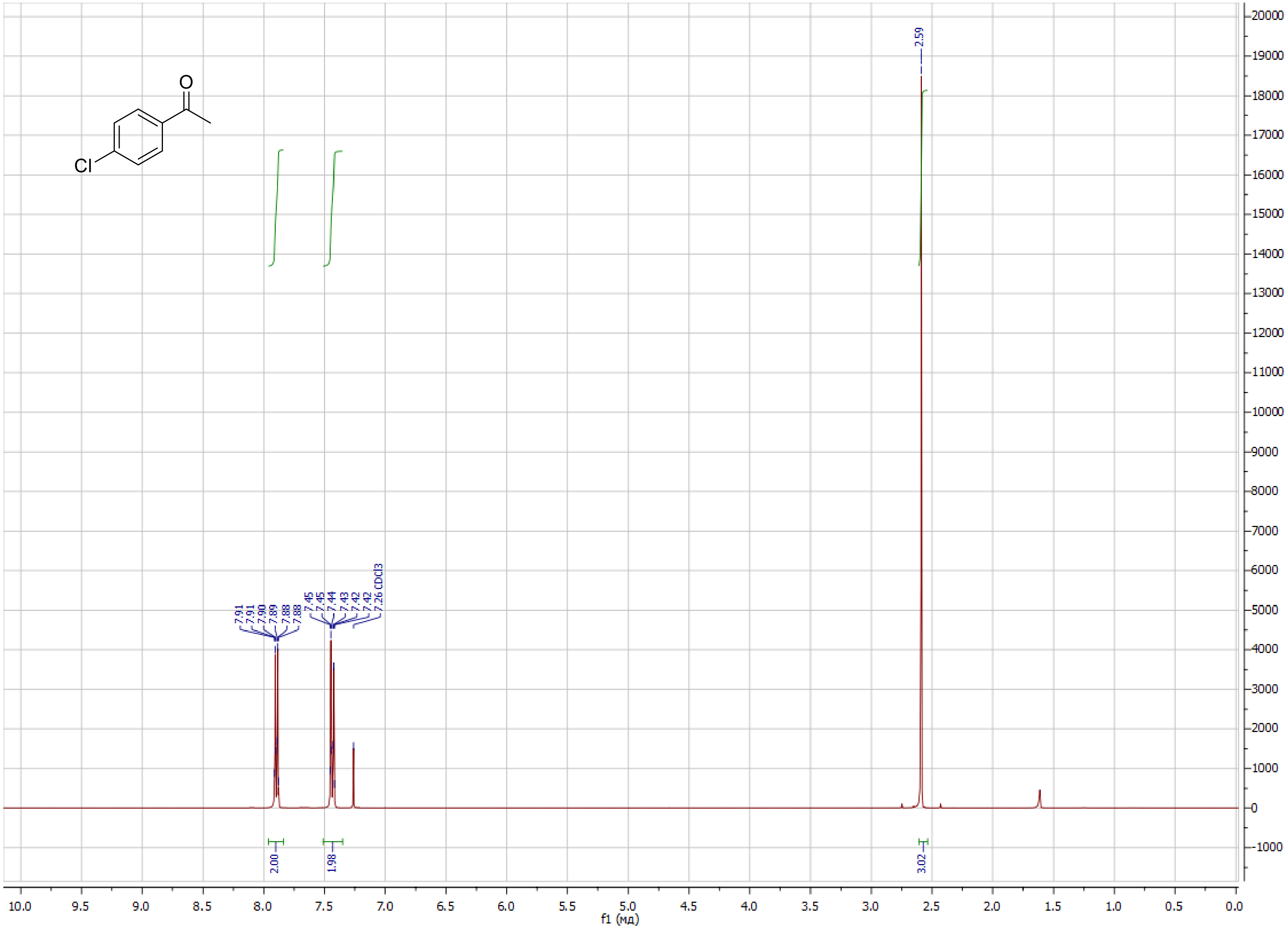

Figure S39. ^{1}H NMR spectrum of acetophenone **7i** in $CDCl_3$ (400 MHz)

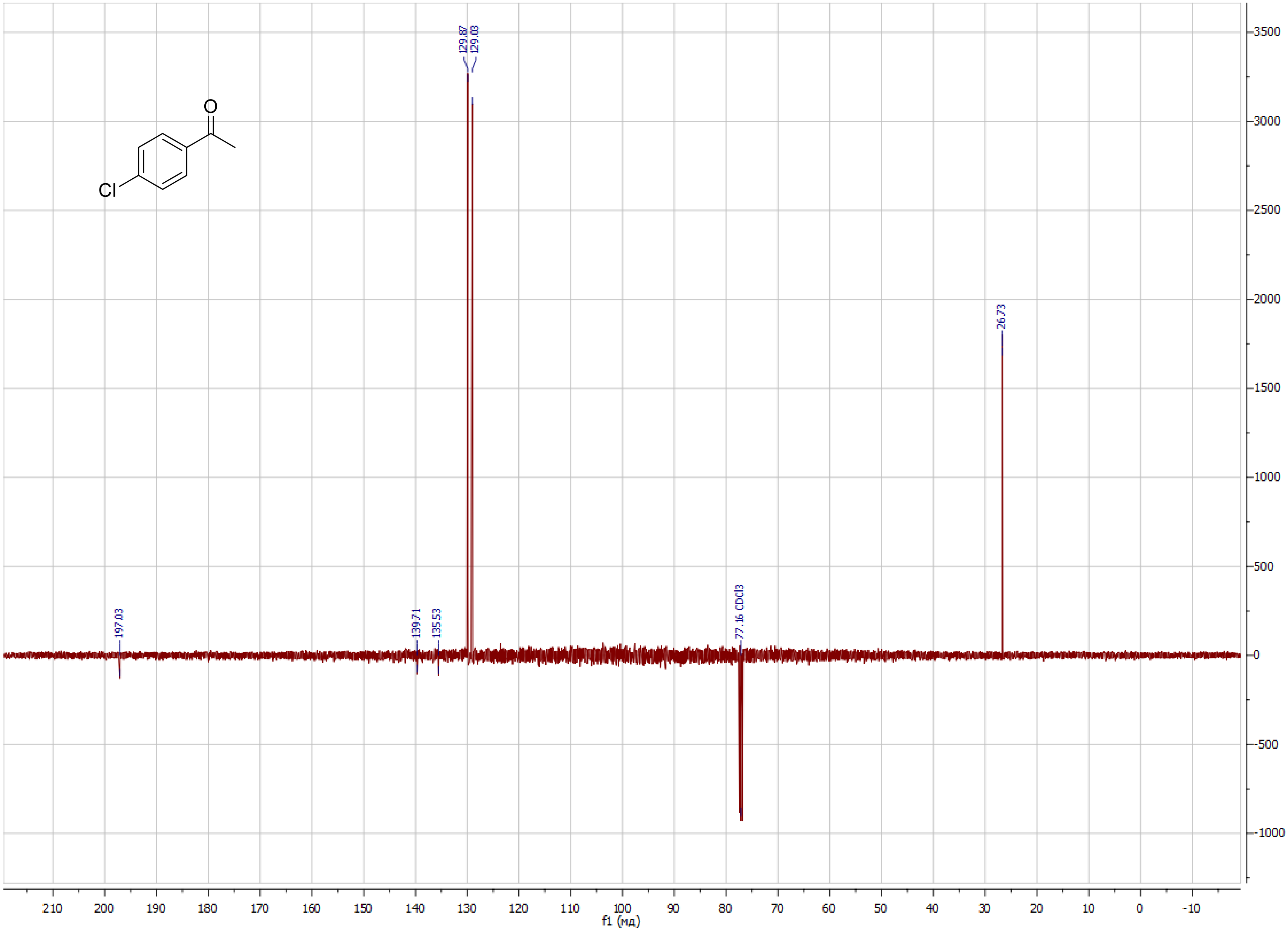

Figure S40. $^{13}C\{^1H\}$ NMR spectrum of acetophenone **7i** in $CDCl_3$ (100 MHz)

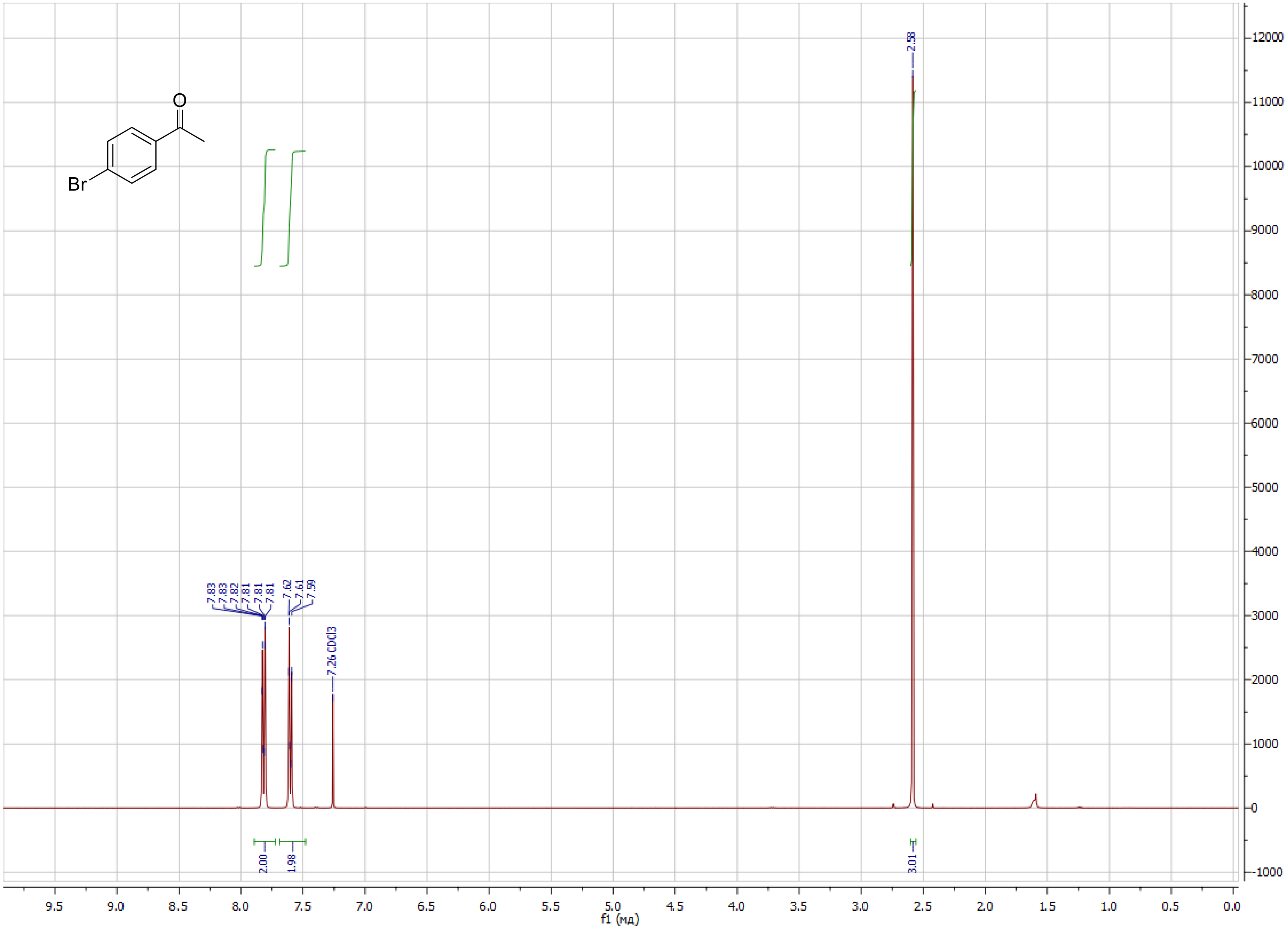

Figure S41. ^{1}H NMR spectrum of acetophenone **7j** in $CDCl_3$ (400 MHz)

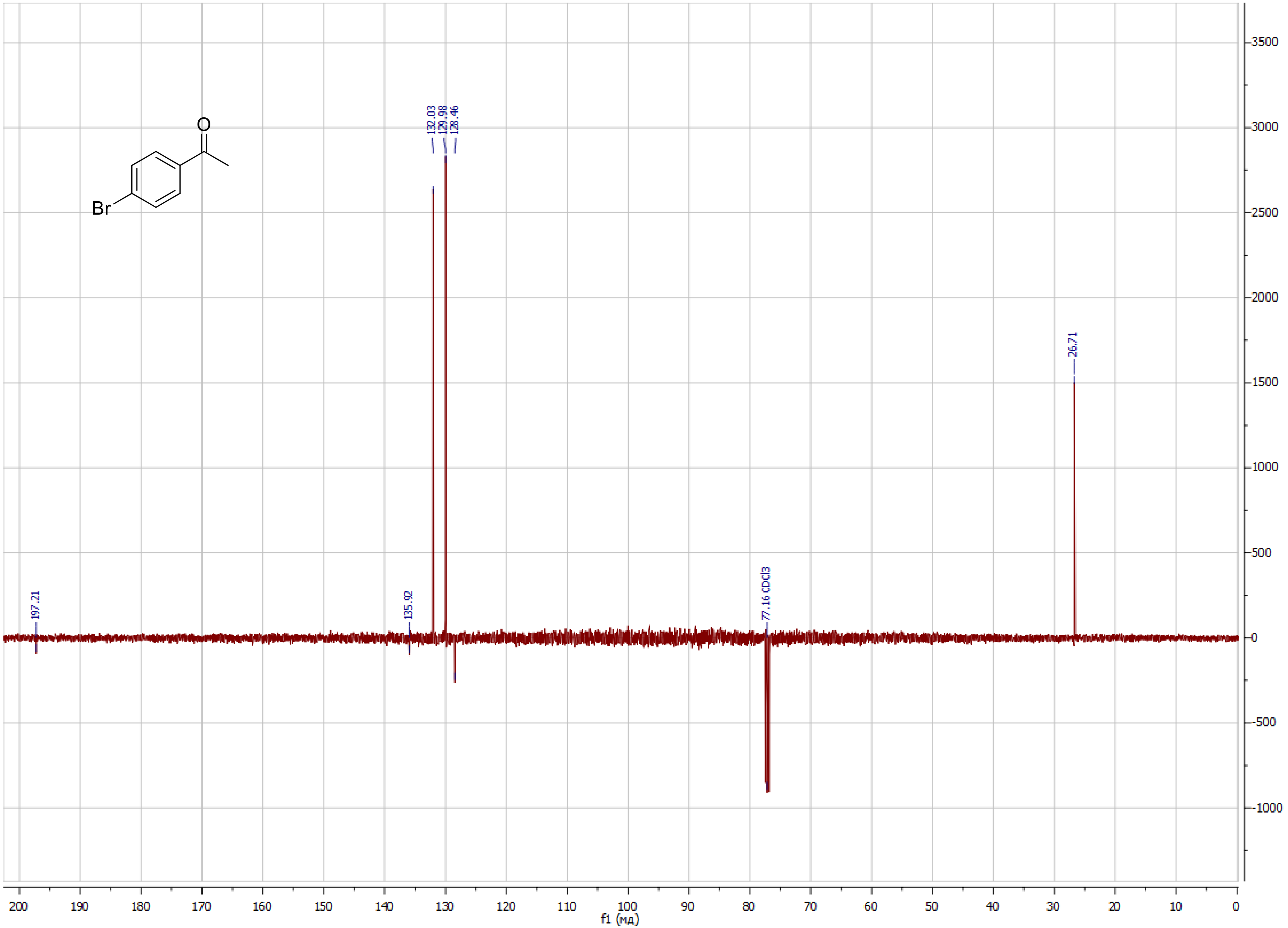

Figure S42. $^{13}C\{^1H\}$ NMR spectrum of acetophenone **7j** in $CDCl_3$ (100 MHz)

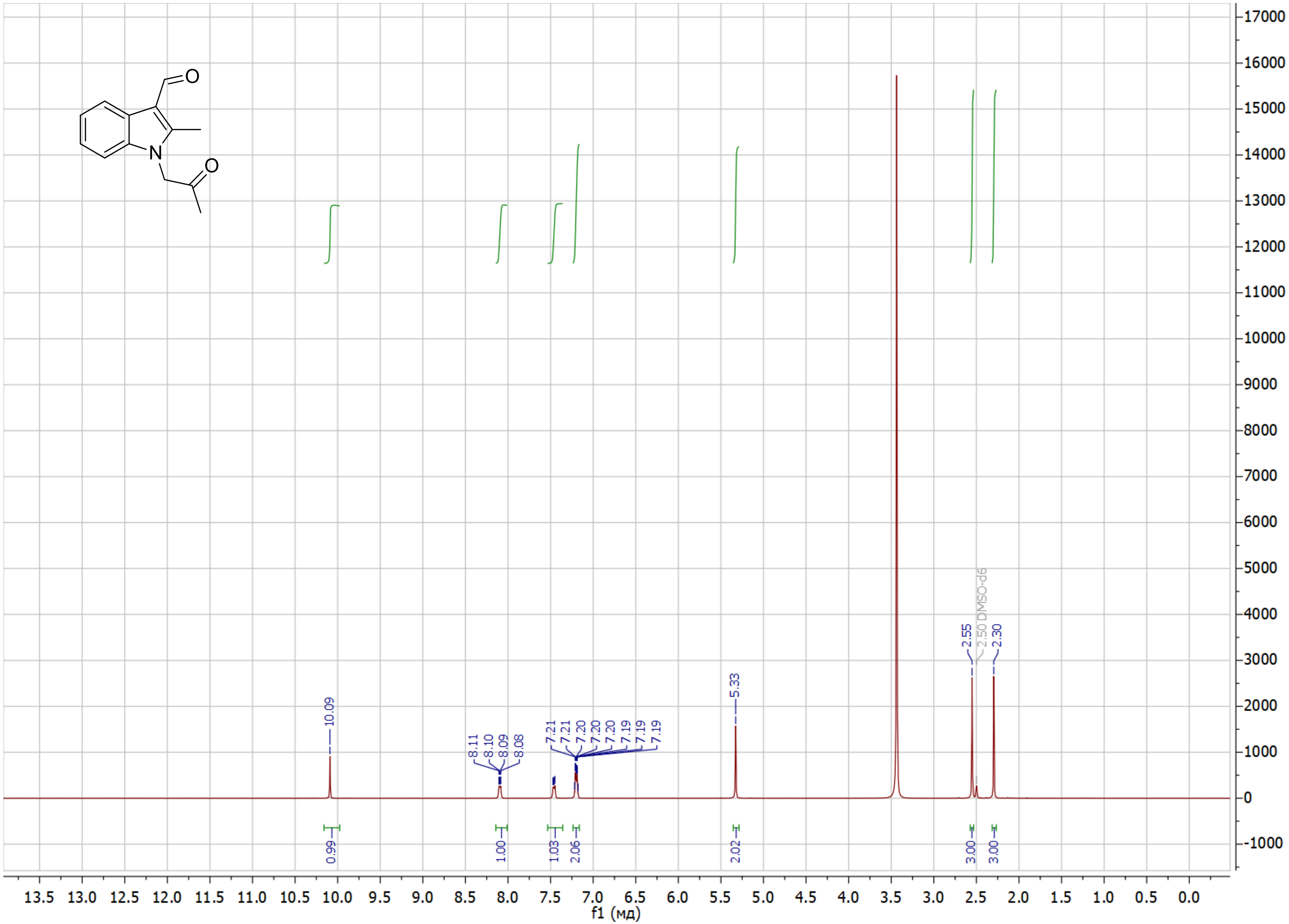

Figure S43. ^{1}H NMR spectrum of acetophenone **7k** in DMSO-d_6 (400 MHz)

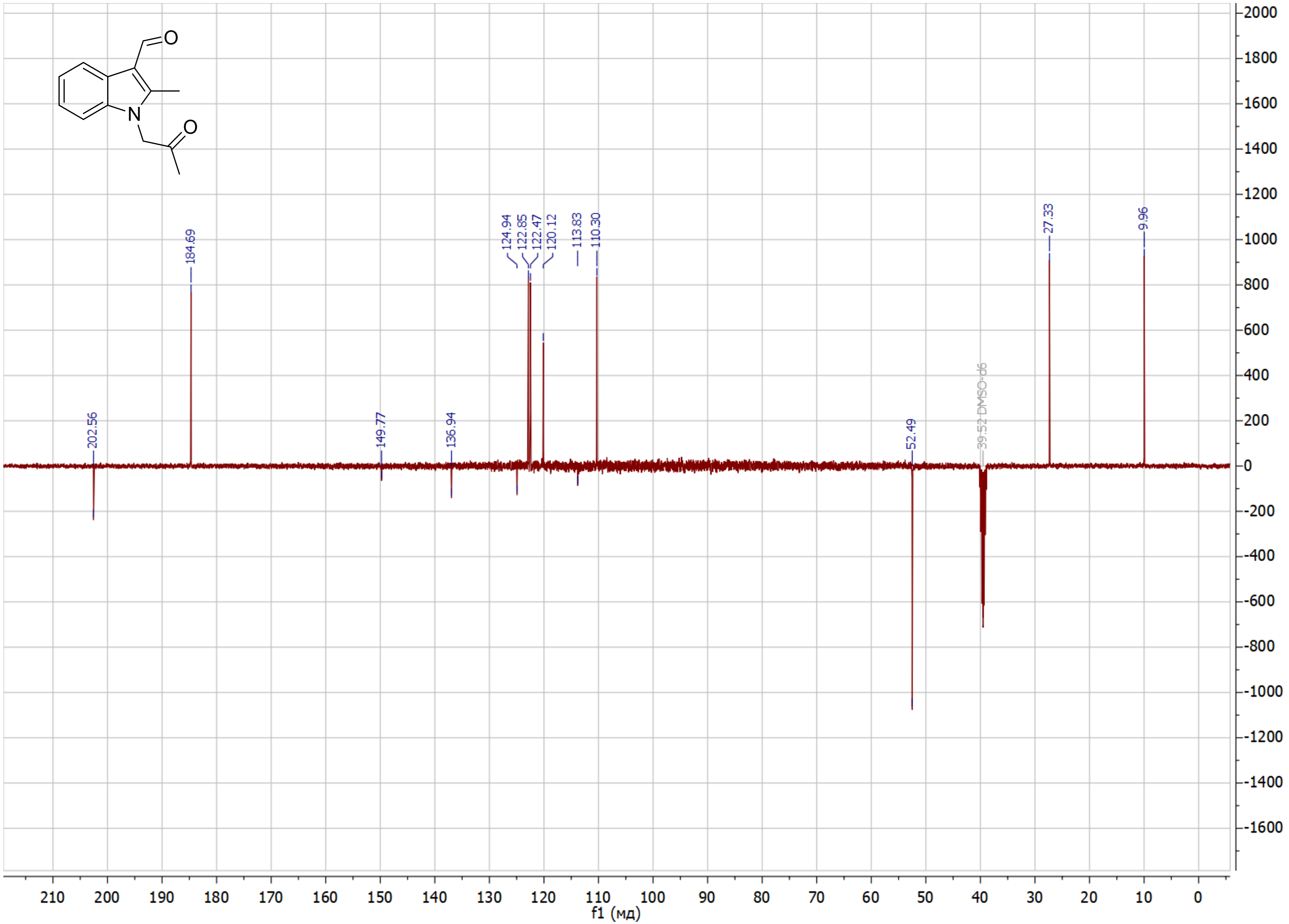

Figure S44. ^{1}H NMR spectrum of acetophenone **7k** in DMSO-d_6 (400 MHz)

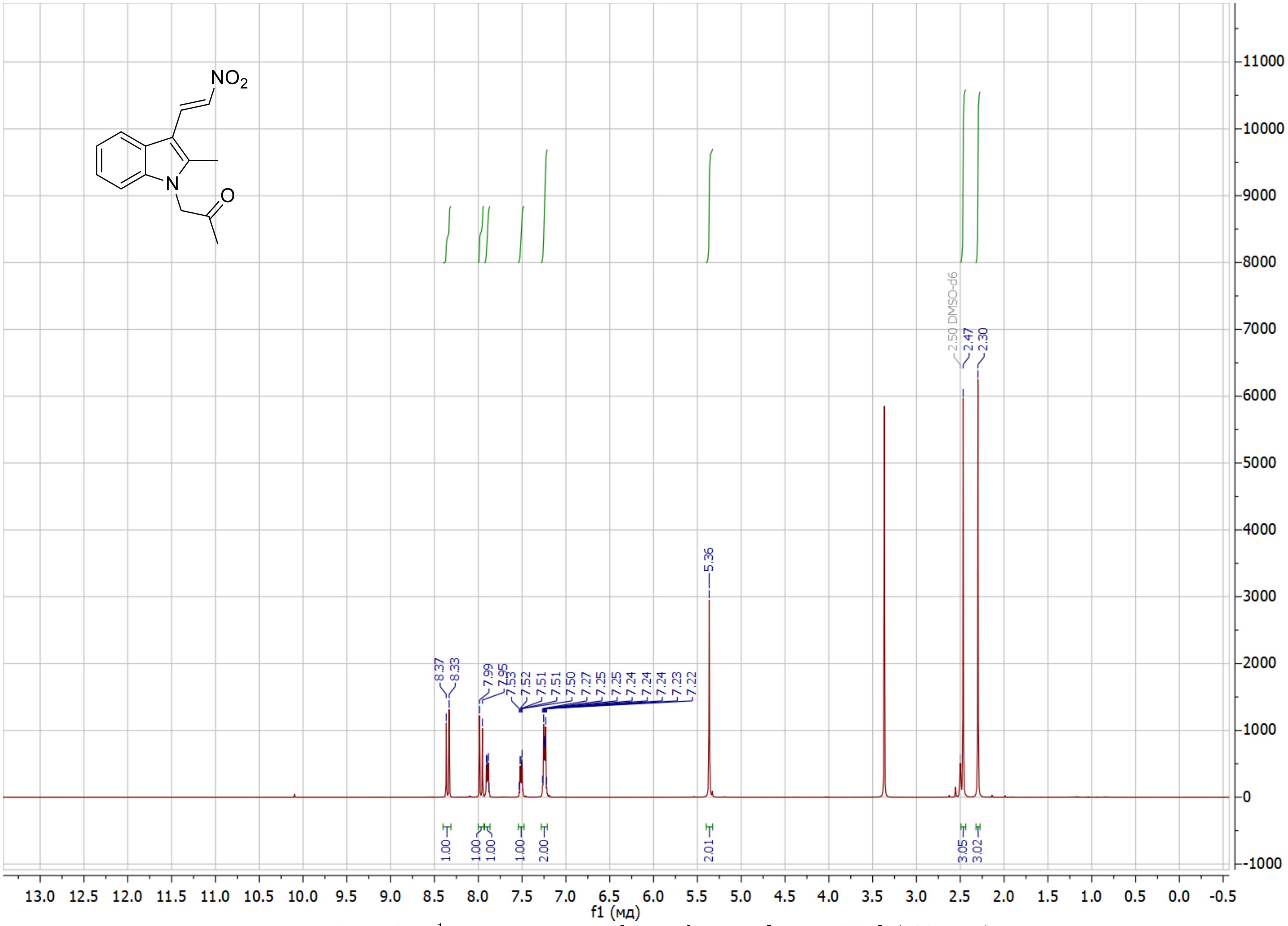

Figure S45. ^{1}H NMR spectrum of acetophenone **7l** in DMSO-d_6 (400 MHz)

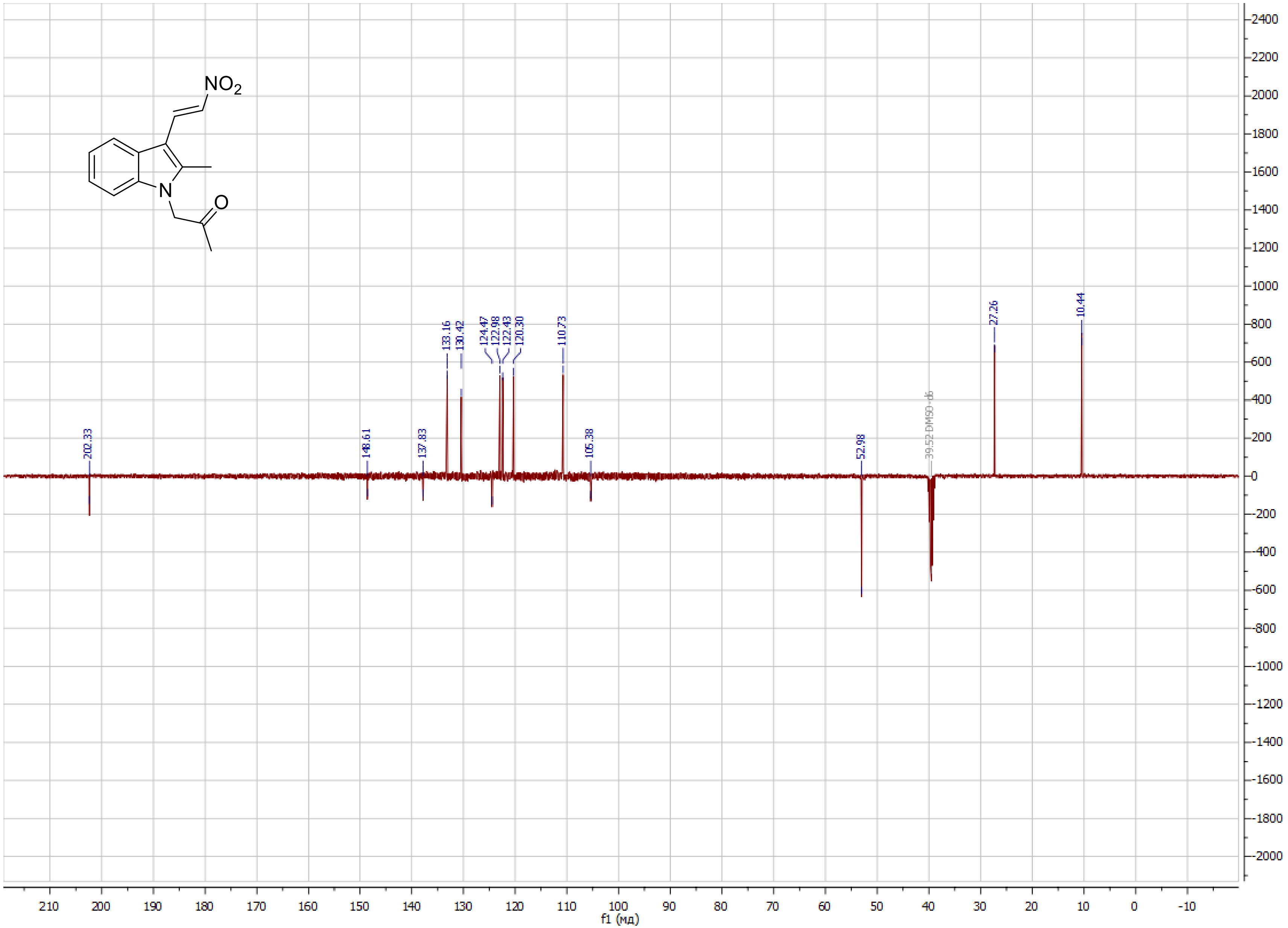

Figure S46. ^{1}H NMR spectrum of acetophenone **7l** in DMSO-d_6 (400 MHz)

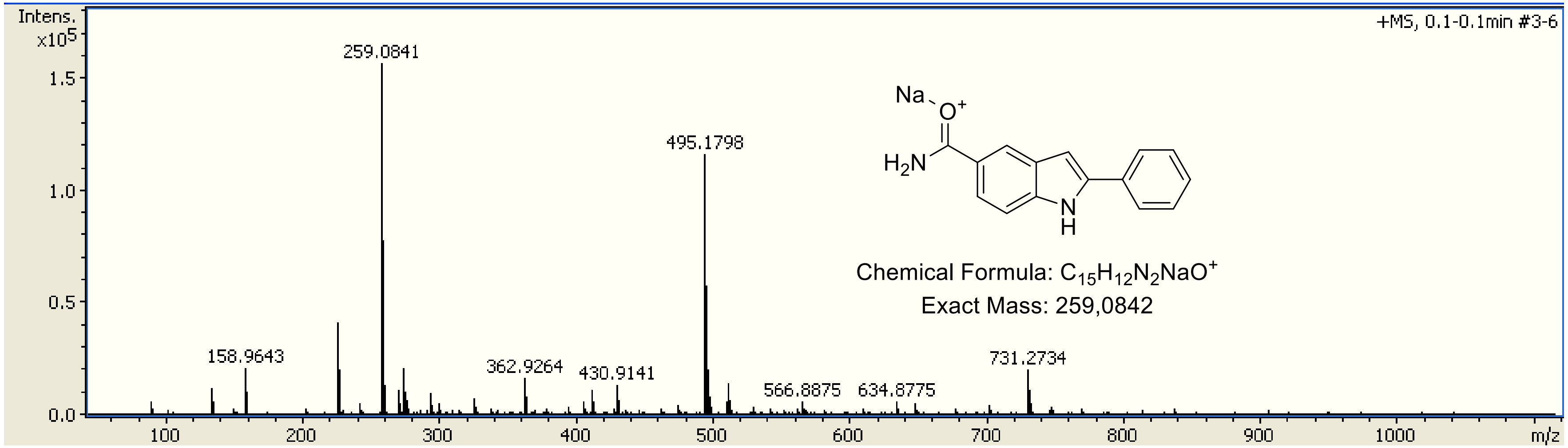

Figure S47. HRMS Chart for 2-Phenyl-1*H*-indole-5-carboxamide **3ai**

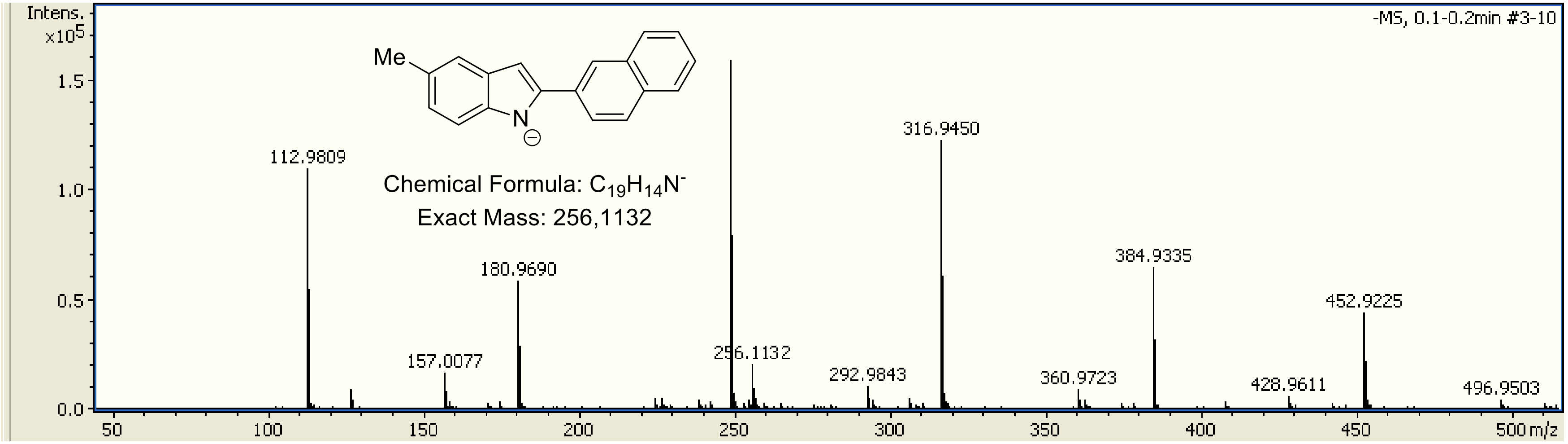

Figure S48. HRMS Chart for 5-Methyl-2-(naphthalen-2-yl)-1*H*-indole **3bc**

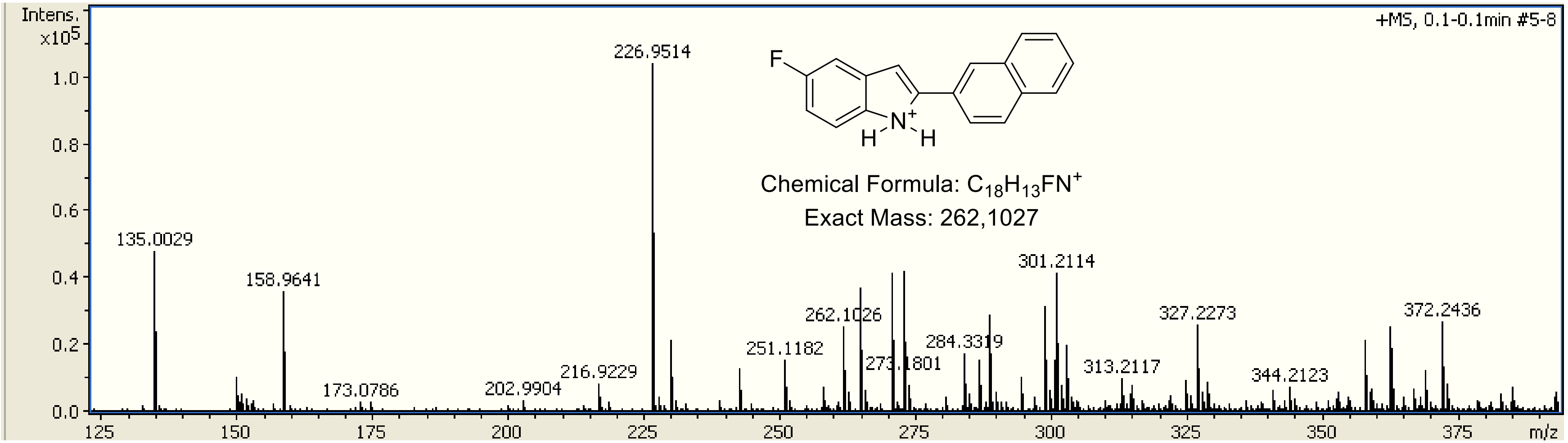

Figure S49. HRMS Chart for 2-(3,4-Dimethylphenyl)-5-fluoro-1*H*-indole **3bf**

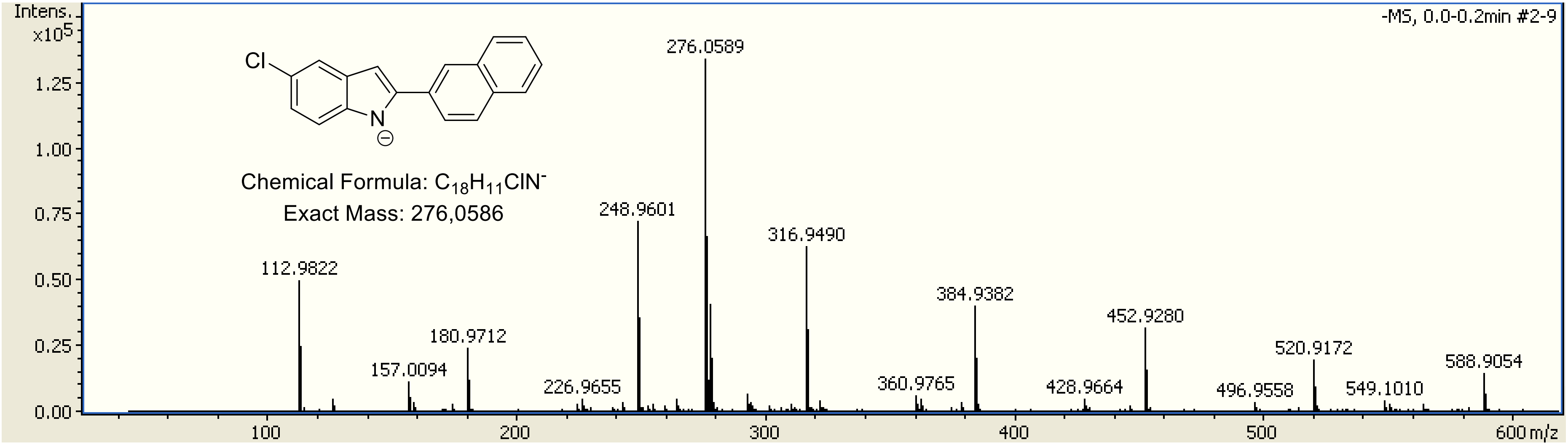

Figure S50. HRMS Chart for 5-Chloro-2-(naphthalen-2-yl)-1*H*-indole **3bg**

HRMS Charts for acetophenones **7**

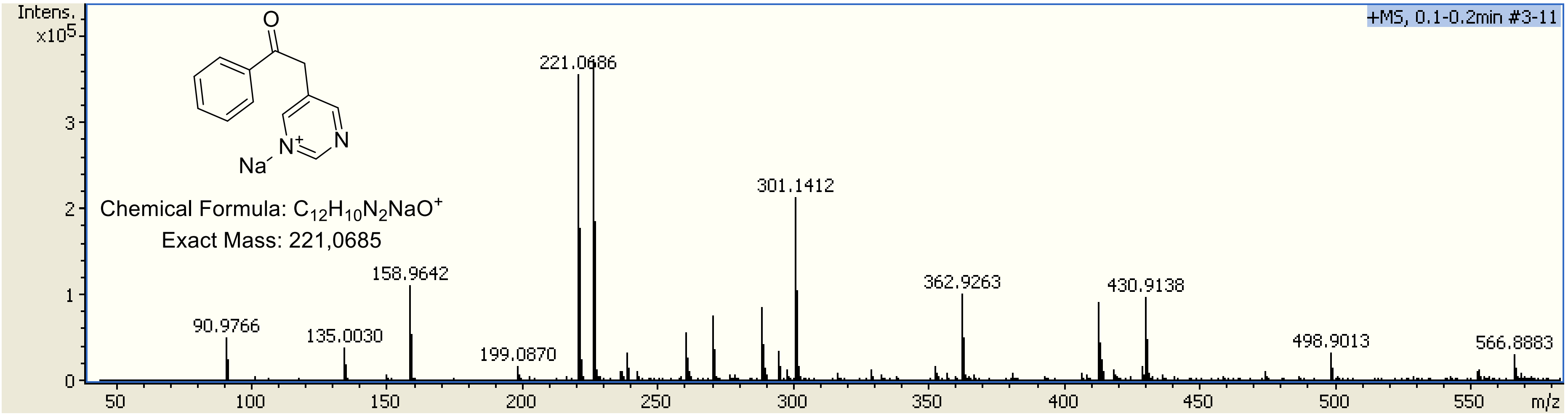

Figure S51. HRMS Chart for 1-Phenyl-2-(pyrimidin-5-yl)ethan-1-one **7f**

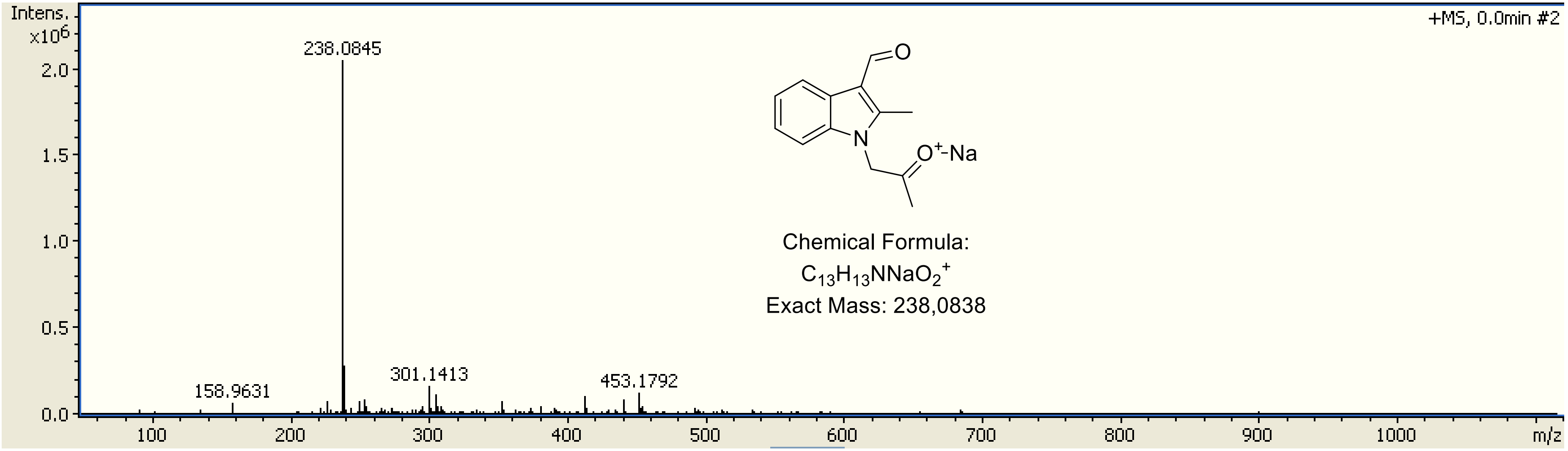

Figure S52. HRMS Chart for 2-Methyl-1-(2-oxopropyl)-1*H*-indole-3-carbaldehyde **7k**

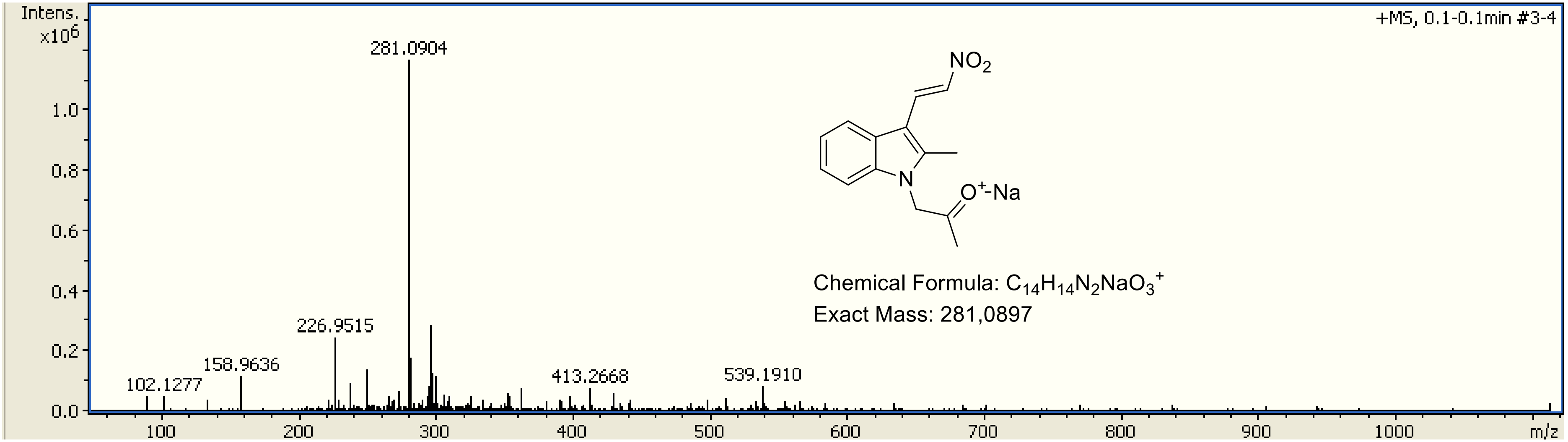

Figure S53. HRMS Chart for (*E*)-1-(2-Methyl-3-(2-nitrovinyl)-1*H*-indol-1-yl)propan-2-one **7l**
